# A hydrophobic Cu/Cu_2_O sheet catalyst for selective electroreduction of CO to ethanol

**DOI:** 10.1038/s41467-023-36261-1

**Published:** 2023-01-31

**Authors:** Guifeng Ma, Olga A. Syzgantseva, Yan Huang, Dragos Stoian, Jie Zhang, Shuliang Yang, Wen Luo, Mengying Jiang, Shumu Li, Chunjun Chen, Maria A. Syzgantseva, Sen Yan, Ningyu Chen, Li Peng, Jun Li, Buxing Han

**Affiliations:** 1 College of Chemistry and Chemical Engineering, Xiamen University, Xiamen, China; 2 Laboratory of Quantum Photodynamics, Department of Chemistry, Lomonosov Moscow State University, Moscow, Russia; 3 Institute of Chemical Sciences and Engineering, École Polytechnique Fédérale de Lausanne (EPFL), Sion, Switzerland; 4 College of Energy, Xiamen University, Xiamen, China; 5 School of Environmental and Chemical Engineering, Shanghai University, Shanghai, China; 6 Institute of Chemistry, Chinese Academy of Sciences, Beijing Mass Spectrum Center, Beijing, China; 7 Beijing National Laboratory for Molecular Sciences, CAS Key Laboratory of Colloid, Interface and Chemical Thermodynamics, CAS Research/Education Center for Excellence in Molecular Sciences, Institute of Chemistry, Chinese Academy of Sciences, Beijing, PR China; 8 University of Chinese Academy of Sciences, Beijing, China; 9 National Engineering Laboratory for Green Chemical Productions of Alcohols, Ethers and Esters, Xiamen University, Xiamen, China; 10 Collaborative Innovation Center of Chemistry for Energy Materials, Xiamen, China

**Keywords:** Electrocatalysis, Materials for energy and catalysis, Energy

## Abstract

Electrocatalytic reduction of carbon monoxide into fuels or chemicals with two or more carbons is very attractive due to their high energy density and economic value. Herein we demonstrate the synthesis of a hydrophobic Cu/Cu_2_O sheet catalyst with hydrophobic n-butylamine layer and its application in CO electroreduction. The CO reduction on this catalyst produces two or more carbon products with a Faradaic efficiency of 93.5% and partial current density of 151 mA cm^−2^ at the potential of −0.70 V versus a reversible hydrogen electrode. A Faradaic efficiency of 68.8% and partial current density of 111 mA cm^−2^ for ethanol were reached, which is very high in comparison to all previous reports of CO_2_/CO electroreduction with a total current density higher than 10 mA cm^−2^. The as-prepared catalyst also showed impressive stability that the activity and selectivity for two or more carbon products could remain even after 100 operating hours. This work opens a way for efficient electrocatalytic conversion of CO_2_/CO to liquid fuels.

## Abstract (web summary)

Tuning electrocatalytic reduction of carbon monoxide to achieve valuable ethanol product with high performance is interesting yet challenging. Here, the authors design a hydrophobic Cu/Cu_2_O sheet catalyst showing a FE of 68.8% and partial current density of 111 mA cm^−2^ for CO reduction to ethanol.

## Introduction

Electrochemical CO_2_ reduction reaction (CO_2_RR) to energy-efficient fuels and chemicals could be a solution to relieve the dependency on fossil fuel and mitigate the greenhouse gas effect^1–4^. Production of single carbon products is relatively simple, e.g., the CO_2_RR to CO is currently being developed for commercial applications^5–8^. Products with two or more carbons (C_2+_ products), such as ethylene, acetic acid and ethanol, are useful chemicals or fuels with obvious economic value. Thus, efficient CO_2_RR to C_2+_ products is of great importance. Cu-based catalysts have been shown to be efficient for converting CO_2_ into C_2+_ products with appreciable selectivity^9–11^. However, research work still needs to focus on reducing the cathodic overpotential and further improving C_2+_ product selectivity^12,13^. CO is known as a key reaction intermediate on the pathway to C_2+_ compounds. The CO reduction reaction (CORR) has received increased attention and showed significant promise since recent progress demonstrated high-rate CORR operation, which raises the attractive prospect of dividing the total conversion of CO_2_ into discrete steps with CO as the intermediate feedstock^14–16^.

Some approaches for CORR have shown improved selectivity to C_2+_^15,17^. For example, CORR to ethylene has been reported with Faradaic efficiency (FE) of up to 52.7% through optimization of cathode structure to facilitate CO diffusion at the surface of the electrode and Cu catalysts^18^. By constraining CO coverage on copper, an ethylene FE of 72% and a partial current density of >800 mA cm^−2^ could be achieved^19^. Recently, ethylene could be formed with FE of 87% ± 3% through the introduction of a polymer that is entrained on the electrode surface^14^. It can be seen that ethylene could be generated with both high FE and high molar production. By contrast, there are few studies on the ethanol formation from CORR. Ethanol is of particular interest as it has high energy density, high market price and consistent global demand^20^. However, for a total current density higher than 10 mA cm^−2^, the FE for ethanol from CORR process still needs to be further improved (Supplementary Table 1)^21^. Also very often the catalysts suffered from poor stability^22^. Exploration of catalysts with high catalytic activity, selectivity and stability for ethanol in CORR process still remains a challenging task^23,24^.

In this work, we construct a stable Cu-based catalyst with high efficiency for ethanol formation in CORR process. Previous reports indicated that surface structure of electrocatalysts has great effect on the performance for CO_2_/CO electroreduction^25,26^. Preoxidation of Cu greatly boosted its intrinsic catalytic properties toward C_2+_ formation in CO_2_RR^27^. Computational studies suggested that Cu^+^ can function synergistically with Cu^0^ to promote C_2_ production because of the easier CO_2_ activation and C-C coupling^28^. Though several approaches were employed to stabilize the Cu^+^ species, the active Cu^+^ species are still very prone to being reduced under CO_2_RR conditions^29^. And for CORR, there are very few studies on whether oxides on the catalyst surface play a crucial role in selectivity improvement. On the other hand, hydrophobicity was proposed as one of the governing factors in CO_2_/CO reduction selectivity^4,18^. The above investigations inspire us to design Cu/Cu_2_O catalyst by adopting a hydrophobic strategy and study the performance of C_2+_ products especially ethanol formation in CORR.

**Fig. 1:**
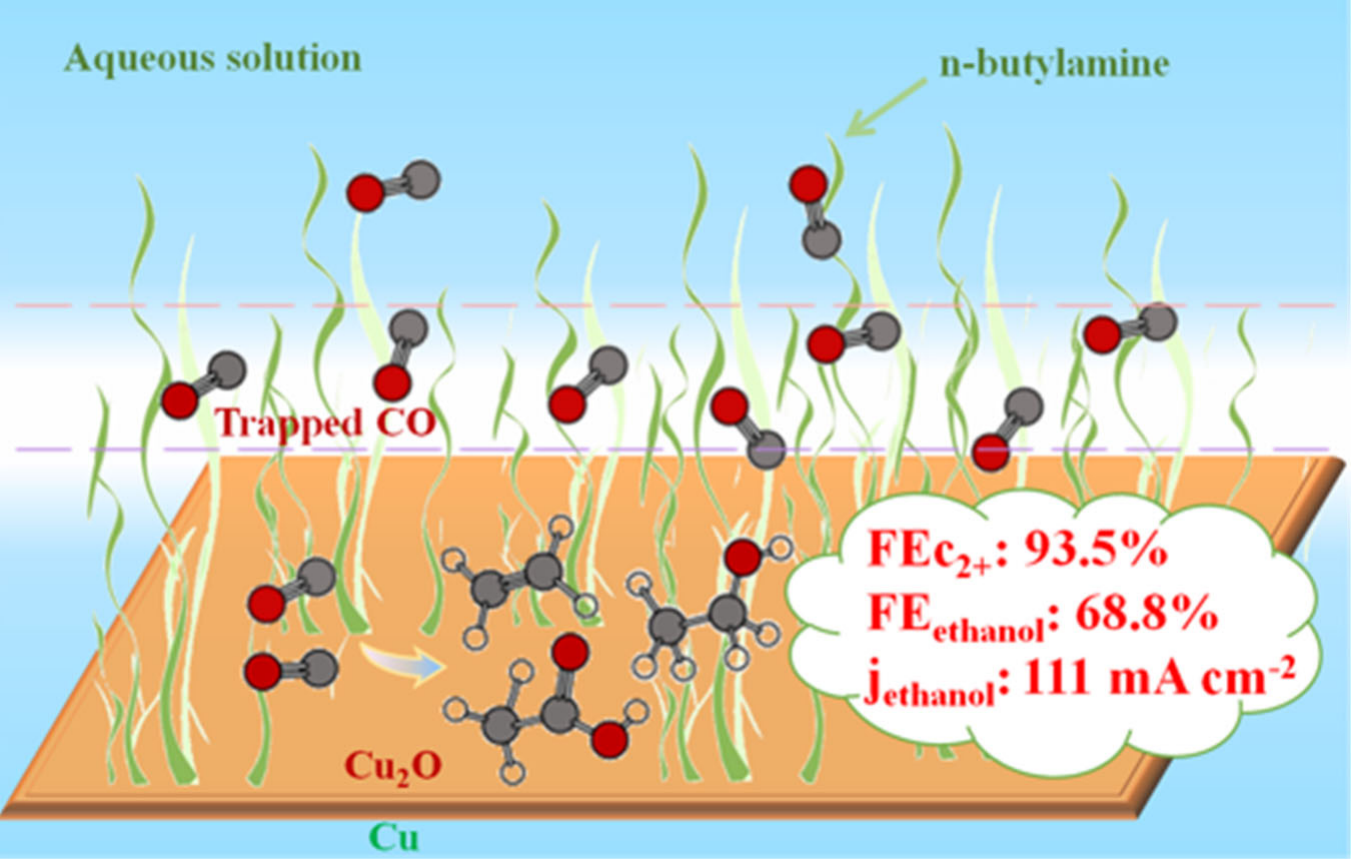
Schematic illustration of the hydrophobic Cu/Cu_2_O catalyst designed for CORR. The n-butylamine on the Cu/Cu_2_O surface is used to trap the CO molecules.

Here we report a facile one-pot synthesis method to synthesize hydrophobic Cu/Cu_2_O catalyst for CO electroreduction. Cu/Cu_2_O coated with n-butylamine was obtained with tunable hydrophobicity. As shown in Fig. 1, the resulted catalyst with appropriate amount of n-butylamine layer offering suitable hydrophobicity would reduce the affinity of water to the electrode and promote the diffusion and affinity of CO to the electrode interface (see Supplementary Tables 2-3 for the diffusion coefficients and solubility of CO in water and n-butylamine), thus H_2_ evolution on the surface could be suppressed to some extent in CO-saturated electrolyte. At −0.7 V vs reversible hydrogen electrode (RHE), the FE of C_2+_ products could reach 93.5% with a current density of 151 mA cm^−2^, including 68.8% of FE for ethanol, 19.6% for ethylene and 5.1% for acetic acid. The FE of ethanol is very high in comparison to all previous reports of CO_2_/CO electroreduction, as can be known from Supplementary Table 1, which could benefit from both the increased CO concentration at the hydrophobic surface and the exposure of Cu_2_O (111) on the surface.

## Results and discussion

### Synthesis and characterization of hydrophobic Cu/Cu_2_O catalyst

**Fig. 2:**
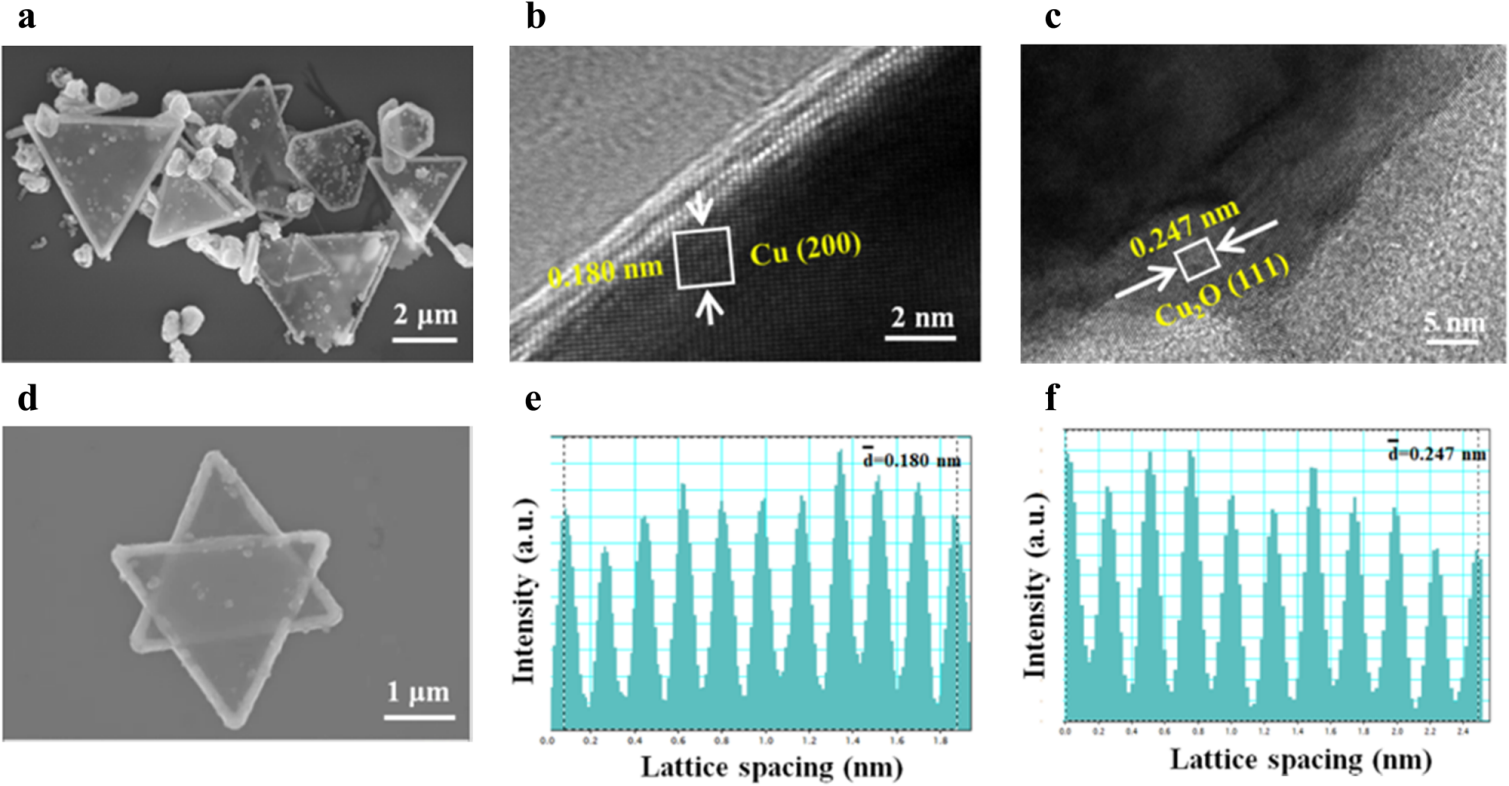
SEM and TEM images for Cu/Cu_2_O-A. **a**, **d** SEM, **b**, **c** TEM and **e**, **f** Intensity profiles measured from the regions marked by the white squares in images b and c, respectively.

The Cu/Cu_2_O catalyst was prepared by one-pot synthesis method through chemical reduction. Glucose aqueous solution with desired amount of polyvinyl pyrrolidone (PVP)/hexadecyl trimethyl ammonium bromide (CTAB) and n-butylamine was added dropwise into the Cu(acac)_2_-N,N-dimethyl formamide (DMF) solution. Then this solution was sealed and heated at 160 °C for 10 h. The Cu/Cu_2_O catalyst (denoted as Cu/Cu_2_O-A) was obtained after cooling down and washing. Scanning electron microscope (SEM) showed the Cu/Cu_2_O mainly exhibited sheet morphology (Fig. 2a, d). The corresponding lattice distances of Cu and Cu_2_O were 0.180 nm and 0.247 nm, respectively, observed by the high-resolution transmission electron microscopy (HRTEM) (Fig. 2b, c, e, f), indicating the co-existence of Cu and Cu_2_O in the as-prepared catalyst. The powder X-ray diffraction measurement (XRD) showed the characteristic peaks of metallic Cu (Supplementary Fig. 1a). The crystal feature of Cu_2_O could hardly be observed by XRD due to the low loading (1.74 wt% according to the Nitrogen-Oxygen analyzer).

**Fig. 3:**
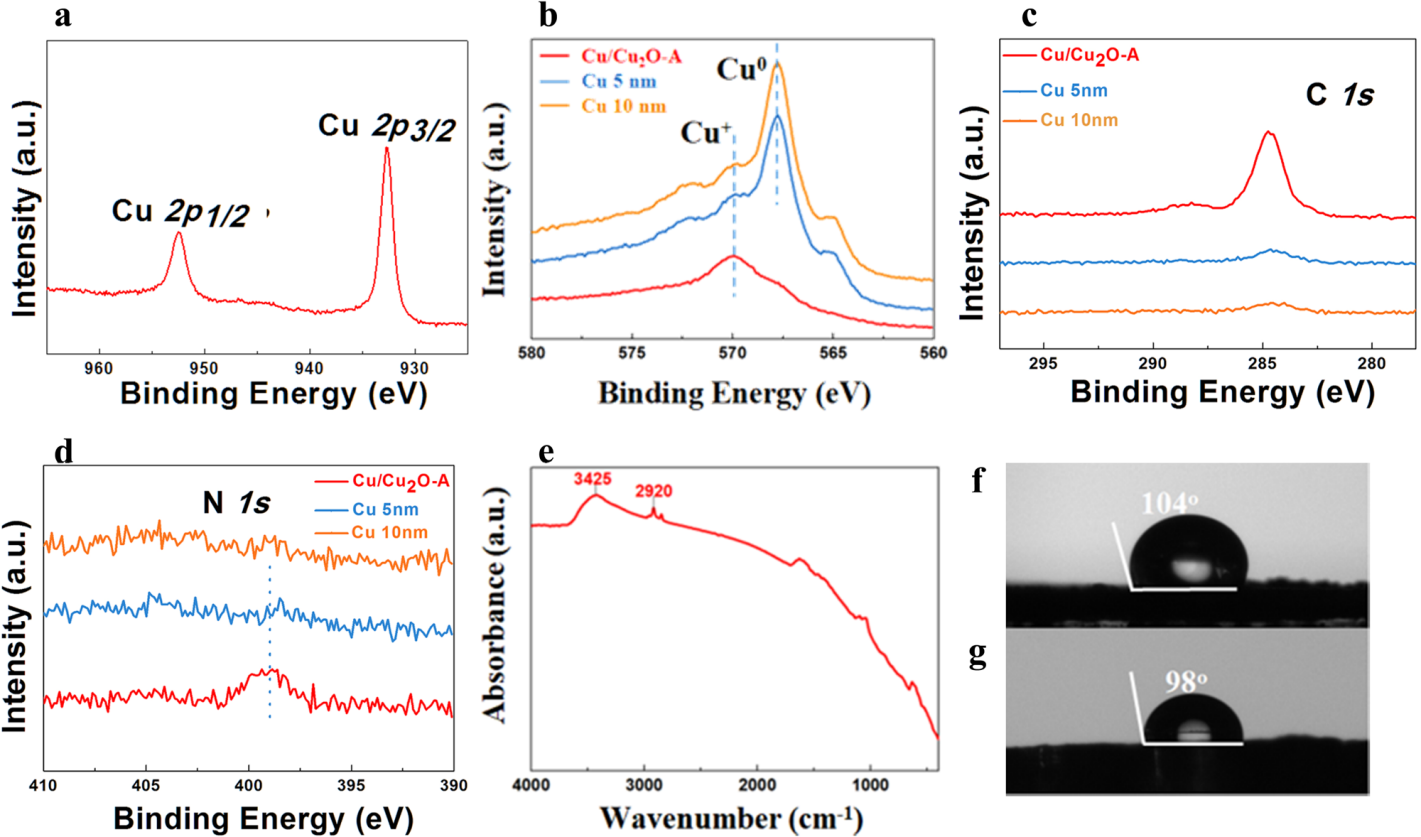
Characterization of the Cu/Cu_2_O-A. **a** Cu *2p* XPS spectrum of the as prepared hydrophobic Cu/Cu_2_O-A catalyst. **b** Cu *LMM*, **c** high-resolution C *1* *s* spectra, and **d** high-resolution N *1* *s* spectra of Cu/Cu_2_O-A (red), Cu 5 nm (blue) and Cu 10 nm (orange) (Cu 5 nm refers to Cu/Cu_2_O-A catalyst etched with 5 nm depth; Cu 10 nm refers to Cu/Cu_2_O-A catalyst etched with 10 nm depth). **e** FT-IR of Cu/Cu_2_O-A. Images of the water contact angle of the as-prepared Cu/Cu_2_O-A catalyst **f** and that after 100 h of CORR at −0.7 V vs RHE **g**.

X-ray photoelectron spectroscopy (XPS) was conducted to investigate the surface chemical composition and elemental valence states of the Cu/Cu_2_O-A catalyst (Fig. 3). The XPS spectrum revealed peaks of Cu species at 952.4 eV (Cu *2p*_*1/2*_) and 932.6 eV (Cu *2p*_*3/2*_) (Fig. 3a). Auger electron spectroscopy (AES) was employed to further evidence that the signal was mainly derived from Cu_2_O^30,31^ (Fig. 3b). As XPS has limitations with regard to probe depth (detection depth often less than 10 nm), we etched the Cu/Cu_2_O-A with 5 nm and 10 nm depths. After etching, it showed the characteristic peaks of Cu^0^, indicating the Cu was covered with Cu_2_O on the surface (Fig. 3b). In addition, the n-butylamine layer on the surface of Cu/Cu_2_O-A catalyst was also confirmed by XPS (Fig. 3c, d). The high-resolution N *1* *s* spectra showed a peak at 398.8 eV (N *1* *s*), which belongs to the n-butylamine coated on the surface. Control spectra had been carried out to prove the N species in N*1s* spectra is from n-butylamine, not from the possible impurities DMF or CTAB (Supplementary Fig. 1b). Moreover, the strong infrared (IR) absorption bands at 3425 and 2920 cm^−1^ (Fig. 3e) further suggested that n-butylamine covered the surface of Cu/Cu_2_O-A^32,33^. A water contact angle (CA) of 104° (Fig. 3f) demonstrates that the n-butylamine-treated Cu/Cu_2_O electrode is impassible to the wetting and this falls into the regime of hydrophobicity of trapping gases^4^. The hydrophobic/hydrophilic property could be easily tuned by the amount of n-butylamine. Modification with larger amount of n-butylamine leads to highly hydrophobic surface of Cu/Cu_2_O catalyst (Supplementary Figs. 2–4 and Supplementary Table 4).

To complement the results from XPS/XRD/HRTEM and the quantification of Cu_2_O phase by the Nitrogen-Oxygen analyzer, we have used X-ray absorption spectroscopy (XAS) to further investigate the Cu species. Unlike XPS (a surface-sensitive technique), XAS is inherently a bulk-sensitive technique and an average phenomenon accounting for all the Cu atoms in the sample. Supplementary Figs. 5a and 5b display the spectra of Cu K-edge X-ray absorption near edge structure (XANES) and extended X-ray absorption fine structure (EXAFS) for the Cu/Cu_2_O-A catalyst and the corresponding Cu standards. They both seem to indicate a high resemblance of the Cu/Cu_2_O-A catalyst with the metallic Cu. Given the nature of the technique, this is well in-line with the XPS Cu_*LMM*_ Auger results showing that a thin Cu_2_O layer (1.74 wt%) covering the metallic Cu surface of the catalyst; under these circumstances, it is reasonable to assume that XAS is hard to detect the low amount of Cu_2_O localized selectively (in a thin layer) over the catalyst surface. This result also explained why it is hard to see the peaks derived from Cu_2_O in the XRD pattern.

All the above results demonstrate that the as-prepared Cu/Cu_2_O-A consists of a metallic copper core with a Cu_2_O shell that is further covered by a layer of n-butylamine. The combination of the reducing agent and stabilizing agent was the key to successful synthesis of Cu/Cu_2_O-A. As the reducing agents, DMF and D- (+) –glucose reduced Cu (II) to Cu (0) or Cu (I). During this process, part of Cu (I) was protected by n-butylamine from further reduction to Cu (0)^31,34^. Meanwhile, n-butylamine was adsorbed on the surface to reduce surface energy and avoid aggregation. What’s more, the layer of n-butylamine acting as the stabilizing agent could restrain oxidation of Cu or Cu_2_O to offer good stability of the oxidation state of the catalyst, which would last 16 months without obvious change under ambient conditions (Supplementary Fig. 6).

### Electrocatalytic CORR performance

**Fig. 4:**
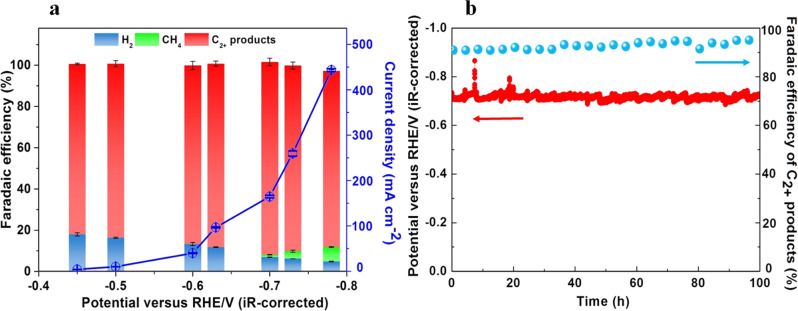
CO electroreduction performance of hydrophobic Cu/Cu_2_O-A. **a** Total current density and Faradaic efficiencies for C_1_ (green), C_2_ products (red) and hydrogen (blue) obtained during CORR in 2.0 M KOH versus applied potential. **b** Stability test over a span of 100 h of electrolysis at 164 mA cm^−2^ in 2.0 M KOH. The loading of Cu/Cu_2_O-A is 2 mg cm^−2^. Error bars represent the standard deviations calculated from three independent measurements.

The CORR performance tests were carried out at various potentials in a flow cell reactor, employing KOH as the electrolyte (Supplementary Fig. 7). Figure 4a shows that the as-prepared hydrophobic Cu/Cu_2_O-A offers good efficiency for CO reduction to C_2+_ products. The maximum FE for C_2+_ products reached as high as 93.5% in 2.0 M KOH at the potential of −0.7 V vs RHE. The FE of ethanol could reach 68.8% with a partial current density of 111 mA cm^−2^ at −0.7 V vs RHE. With the increasing of the applied overpotentials, the rapid growth of current densities indicated that the mass transfer resistance of CO was low.

Moreover, the hydrophobic Cu/Cu_2_O-A showed a stable C_2_ species FE for 100 h (Fig. 4b). Impressively, even after 100 h of electroreduction reaction, the amount of Cu^+^ on the catalyst surface was still the same as that in the virgin sample, reflecting the high stability of hydrophobic Cu/Cu_2_O-A and the protective effects of n-butylamine (Supplementary Figs. 8-10). What’s more, to ensure the repeatability, another two independent stability measurements were carried out under the same conditions and time duration (Supplementary Fig. 11). The water CA (98°) on the catalyst after 100 h of CORR at −0.7 V vs RHE was very close to that of the fresh one, indicating that the hydrophobic surface of Cu/Cu_2_O-A was well maintained during the CORR process (Fig. 3g). XPS analysis and elemental analysis before and after the 100 h of electrolysis exhibited similar contents of C and N on the surface of the hydrophobic Cu/Cu_2_O-A, further confirming the well-maintenance of the hydrophobic butylamine layer on the surface (Supplementary Fig. 8 and Supplementary Table 4). The in-situ Raman spectra of the catalyst during the electroreduction with the laser excitation at 532 nm were obtained. At the applied potential of -1.77 V vs Ag/AgCl for 12 min, the characteristic peaks at 220 and 632 cm^−1^ for Cu_2_O retained, suggesting Cu^+^ is persistent under electrocatalytic conditions (Supplementary Fig. 12). At the same time, due to the low polarity, CO tends to stay close to the hydrophobic tail of n-butylamine. Furthermore, reports have shown that the hydrophobic layer could trap gas to form an electrolyte-electrode-gas triple phase boundary^4,35,36^. Thus in this study we speculated that n-butylamine could act as a stabilizer for Cu^+^ and form voids that trapped CO to offer an electrolyte-electrode-gas triple phase boundary, which might enhance the long-term stability of the Cu/Cu_2_O catalyst. To confirm that CO was the carbon source of C_2_ products, we conducted the blank experiments using N_2_ to replace CO in the electrolysis. The experiments showed that no product was formed in the electrolysis when using N_2_ (Supplementary Fig. 13). To further verify that the product was derived from CO reduction, isotope labeled ^13^CO experiment was conducted using Cu/Cu_2_O-A. From the mass spectra, we can observe ^13^C signal for the main liquid product ethanol (Supplementary Fig. 14) and the main gas product ethylene (Supplementary Fig. 15), revealing that the C_2_ products were derived from CO rather than other C-based chemicals in our reaction system.

**Fig. 5:**
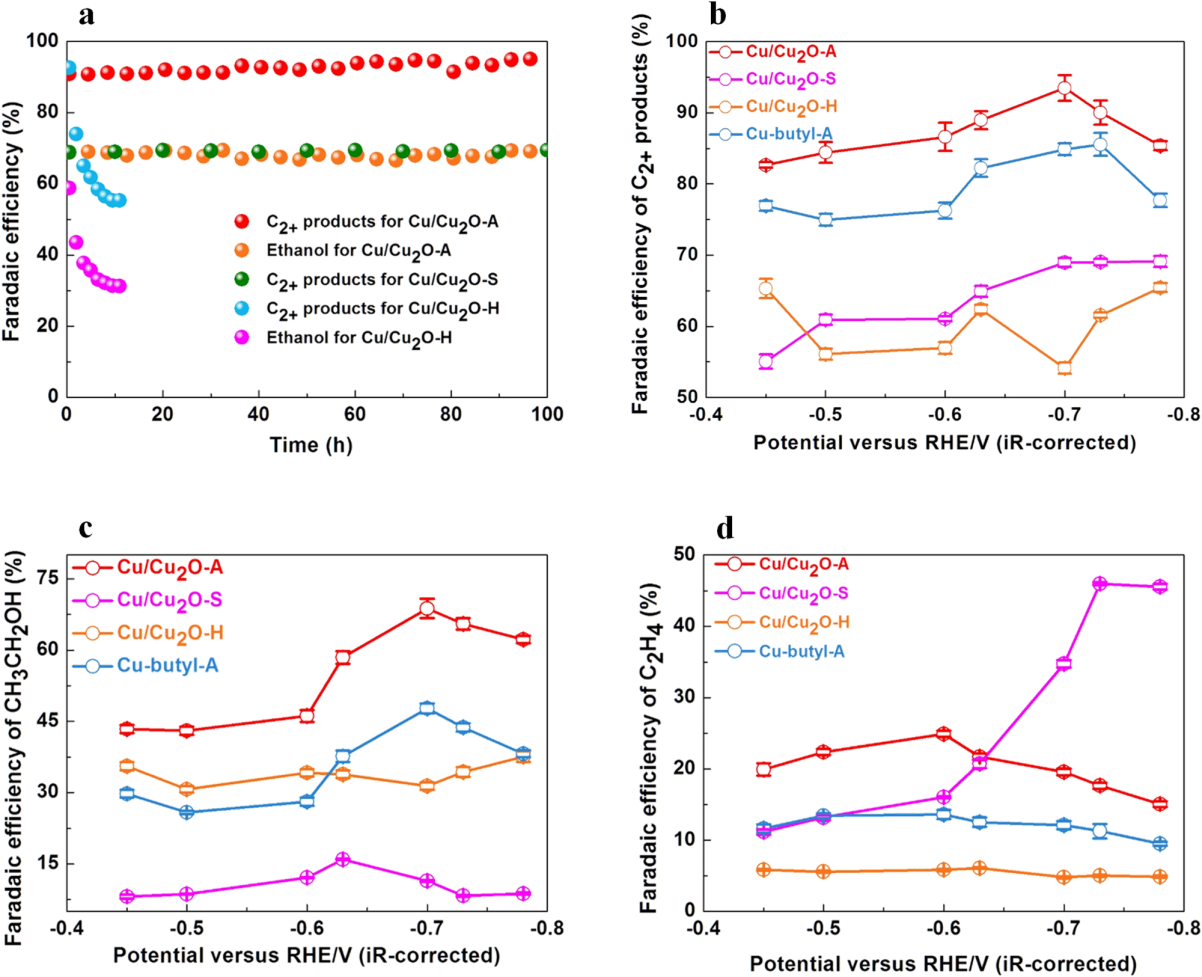
Faradaic efficiency of various products for Cu-based catalysts. **a** Stability test of electrolysis in 2.0 M KOH for Cu/Cu_2_O-A, Cu/Cu_2_O-S and Cu/Cu_2_O-H. **b** FE of C_2+_ products, **c** FE of ethanol, and **d** FE of ethylene for CO electroreduction on Cu/Cu_2_O-A (red), Cu/Cu_2_O-S (purple), Cu/Cu_2_O-H (orange) and Cu-butyl-A (blue). The loading of Cu/Cu_2_O-A is 2 mg cm^−2^. Error bars represent the standard deviations calculated from three independent measurements.

In order to reveal the role of the hydrophobicity on the high C_2+_ FE and good stability of the Cu/Cu_2_O catalyst we further synthesized another two samples with different hydrophilicity and hydrophobicity denoted as Cu/Cu_2_O-S (highly hydrophobic) and Cu/Cu_2_O-H (hydrophilic) by adjusting the added amount of n-butylamine. Cu/Cu_2_O-S and Cu/Cu_2_O-H showed water CA of 130° and 50°, respectively (Supplementary Fig. 4). These two samples showed similar XRD patterns and FT-IR spectra with Cu/Cu_2_O-A but with different amount of n-butylamine on the surface (Supplementary Figs. 2 and 6). Figure 5a shows the stability test of all of the three samples (Cu/Cu_2_O-A, Cu/Cu_2_O-S and Cu/Cu_2_O-H) over 100 h; while the data present in Fig. 5b-d for Cu/Cu_2_O-H were collected after 10 h when the performance was relatively stable. Cu/Cu_2_O-A, Cu/Cu_2_O-S and Cu/Cu_2_O-H with different hydrophobicity showed good FEs and current densities of C_2+_ products, offering 93.5% (at −0.7 V), 69.1% (at −0.78 V) and 65.4% (at −0.78 V) FEs, respectively (Fig. 5b and Supplementary Figs. 16 and 17). While the hydrophobic Cu/Cu_2_O-A and Cu/Cu_2_O-S could give stable FEs of C_2+_ products over 100 hours, hydrophilic Cu/Cu_2_O-H was quite unstable as the gas diffusion layer became flooded and then the activity quickly decreased during operation^35^, indicating the suitable hydrophobicity is vital for good stability (Fig. 5a and Supplementary Fig. 18). Both Cu/Cu_2_O-A and Cu/Cu_2_O-H showed high FEs of ethanol, reaching maximum value of 68.8% (at -0.7 V) and 37.6% (at -0.78 V) (Fig. 5c and Supplementary Fig. 17). Cu/Cu_2_O-S with the highest hydrophobicity, however, favors producing ethylene (46%) and shows quite low FE (8.3%) of ethanol at -0.73 V vs RHE (Fig. 5c, d and Supplementary Fig. 17). At the same time, Cu/Cu_2_O-H showed much higher H_2_ FE because its high hydrophilicity increases the affinity of water to the catalyst and thus decreases the number of the active sites for CORR (Supplementary Figs. 18 and 19). Therefore, a proper hydrophobicity can reduce the affinity of water to the electrode, enhance the stability and at the same time promote the diffusion of CO to the water-electrode interface, but too high hydrophobicity favors producing ethylene over ethanol, and too high hydrophilicity decreases the stability of the electrode.

After addressing the role of hydrophobicity, hydrophobic Cu-butylamine (denoted as Cu-butyl-A) without Cu_2_O was synthesized to identify the role of the Cu_2_O. Cu-butyl-A showed similar hydrophobicity (CA = 97°) with Cu/Cu_2_O-A. The XPS spectra confirmed there was no Cu_2_O in this Cu-butyl-A sample (Supplementary Figs. 20 and 21). At -0.7 V vs RHE, compared with that of Cu/Cu_2_O-A, Cu-butyl-A exhibited a lower FE of 84.9% for C_2_ species and lower ethanol FE of 47.7% (Fig. 5b, c and Supplementary Fig. 22). These results indicated the importance of Cu_2_O on promoting the C_2_ products and especially on increasing the selectivity of ethanol during the CORR. This urged us to study the possible mechanism of the Cu_2_O enhancing the selectivity of ethanol by theoretic calculation.

### Density functional theory calculations

It was reported that different facets of Cu_2_O could greatly affect the electrocatalysis performances^37,38^. Since the Cu_2_O (111) was clearly observed in hydrophobic Cu/Cu_2_O sample (no other facets were evidenced), we investigated the feasibility of ethanol formation on the stoichiometric oxygen-terminated (111) surface of Cu_2_O by using the density functional theory (DFT) calculations. The Gibbs free energy profile for the proposed mechanism was modeled within DFT, employing computational hydrogen electrode (CHE) approach^39^ to account for the electrode potential -0.70 V vs RHE. The considered (111) surface comprises under-coordinated Cu_cus_ atoms, which is important for the adsorption of carbon-containing intermediate species. The energy of adsorption on the (111) Cu_2_O surface is particularly favorable for the hydrogen-rich intermediates, such as *C_2_OH, *C_2_H_2_O, *C_2_H_3_O and *C_2_H_5_O. In particular, Cu_cus_ sites are important for stabilizing these species through the interaction with non-polar carbon C, to which H atoms are progressively attached. The detailed DFT calculation information and discussion is shown in supplementary information (Supplementary Figs. 23, 24). It should be noted that except the experimental observations, the DFT calculations also suggest the partial reduction of Cu_2_O surface is hindered by the presence of nBu-NH_2_, while the total reduction is completely unfavorable even at the low chemical potential of water (Supplementary Table 5 and Supplementary Figs. 25, 26).

In summary, hydrophobic Cu/Cu_2_O prepared through a facile one-pot synthesis method showed good selectivity for CO electroreduction into C_2_ species (93.5% FE) in 2.0 M KOH electrolyte. The FE of ethanol (68.8%) ranked this material among the most efficient catalysts for electroreduction of CO_2_/CO. This hydrophobic catalyst exhibited good stability in C_2+_ FE for operating over 100 hours. On the basis of the experimental results and the DFT calculation, it is concluded that suitable hydrophobicity and exposure of Cu_2_O (111) were the governing factors of CO electroreduction selectivity and stability. We anticipate that the route of this work has promising potential for converting CO into ethanol in a more selective and energy-efficient way. We also believe that the protocol to modify surface hydrophobicity can be used to prepare other efficient electrocatalysts.

## Methods

### Synthesis of hydrophobic Cu/Cu_2_O (denoted as Cu/Cu_2_O-A)

In a typical synthesis of hydrophobic Cu/Cu_2_O, 500 mg Cu(acac)_2_ was added into a solution of 100 mL dimethylformamide (DMF). After vigorous stirring for 5 min, 20 mL H_2_O containing 50 mg glucose, 30 mg hexadecyl trimethyl ammonium bromide (CTAB), 110 mg polyvinyl pyrrolidone (PVP) and 5 mL n-butylamine was added by dropwise with vigorous stirring. After half an hour, the mixture was transferred into a 200 mL Teflon-lined autoclave, sealed and heated at 160 °C for 10 h. Then the system was allowed to cool down to room temperature naturally, and the mixture was collected by centrifuging, washing with ethanol and cyclohexane, and finally dried in vacuum for future characterization.

### Synthesis of highly hydrophobic and hydrophilic Cu/Cu_2_O (denoted as Cu/Cu_2_O-S and Cu/Cu_2_O-H, respectively)

As for the synthesis of highly hydrophobic and hydrophilic Cu/Cu_2_O, the experimental process is similar to that hydrophobic Cu/Cu_2_O, except only 15 mL n-butylamine and 1 mL n-butylamine were used, respectively.

### Synthesis of hydrophobic Cu-butyl-A

20 mg hydrophobic Cu/Cu_2_O was placed in a quartz tube and heated to 120 °C at a flow of 5 °C min^−1^ under H_2_/Ar flow (V/V = 5:95) and kept at this temperature for 2 hours, then cooled down to room temperature. The powder was transferred into a solution containing 100 mL DMF, 20 mL H_2_O and 5 mL n-butylamine and stirred for half an hour. Lastly, the mixture was transferred into a 200 mL Teflon-lined autoclave, sealed and heated at 160 °C for 10 h. Then the system was allowed to cool down to room temperature naturally, and the mixture was collected by centrifuging, washing with cyclohexane, and finally dried in vacuum for future characterization.

### Preparation of electrode

To construct the electrode, the catalyst ink was prepared by dispersing 20 mg of Cu/Cu_2_O and 80 μL of 5 wt% Nafion solution into 1 mL of water/ethanol (V/V = 4:1) solution with 3 h of sonication, and 0.1 mL of the catalyst ink was loaded on a carbon fiber paper (the gas diffusion layer, YSL-30T, 1 cm × 2 cm).

### Electrocatalytic analysis

The CO electroreduction performances were implemented with a three-compartment electrochemical flow cell (Supplementary Fig. 7) connected to an electrochemical workstation (CHI760e). The as-prepared electrode, Ag/AgCl (saturated KCl) and nickel foam were used as the working electrode, reference electrode and anode, respectively. A total of 30 mL of KOH solution (0.5 M, 1.0 M, or 2.0 M) was applied as the electrolyte at both the cathode and anode sides and the cathode and anode chambers were separated by an anion exchange membrane (FFA-3, Fumatech) and the electrolytes were circulated by peristaltic pumps at the flows of 5 mL min^−1^. The CO gas was introduced into the cathode with flow rate was 20 mL min^−1^ controlled using a mass flow controller (SevenStar D07-7). CO could diffuse into the interface between the cathode and electrolyte. All potentials were measured against the Ag/AgCl reference electrode (saturated KCl). The gas-phase products were analyzed from the outlet of the CO chamber connecting to a gas chromatography (GC) during electrolysis, and the liquid- phase products were analyzed from the electrolyte post-reaction using ^1^H NMR. The Faradaic efficiency (FE) was calculated based on the Eq. (1)^4^.

1 $${FE}\left(\%\right)=\frac{{n}_{{{{{{\rm{product}}}}}}}\times {n}_{{{{{{\rm{electron}}}}}}}\times F}{\triangle Q}\times 100\%$$

where *n*_product_ is the amount of the product (mol), *n*_electron_ is the number of electrons transferred from CO/H_2_O into products, *F* is the Faradaic constant (C mol^−1^). *∆Q* is the total amount of the charge that changes during the reaction.

Potentials were converted to the RHE using $E\left({{{{{\rm{RHE}}}}}}\right)=E\left({{{{{\rm{Ag}}}}}}|{{{{{\rm{AgCl}}}}}}\right)+0.197+{pH}\times 0.0591$. The ohmic-drop correction of the potentials applied was carried out manually using the resistance measured by the electrochemical impedance spectroscopy under open circuit potentials once the electrolysis was completed. All of the electrocatalytic reactions were implemented at ambient pressure and temperature and 85% ohmic resistance correction was applied in all the measurements. The gas products were collected and analysed every 10 or 30 min during the reaction.

### DFT calculation

All calculations have been performed within the Density Functional theory (DFT) applying PBE functional^40^. The absorption studies on the Cu_2_O surface were carried out using Quantum Espresso *ab* initio simulation package^41^. The non-polar oxygen-terminated stoichiometric (111) surface of Cu_2_O was cut out from a cubic bulk Cu_2_O structure with 4.301 Å lattice parameter obtained by full geometry relaxation with PBE functional. The constructed 3-layer 1×1 surface, comprising 18 atoms, with *a* = *b* = 6.082 Å lattice parameters and more than 20 Å of vacuum in z-direction was relaxed at DFT-PBE level. The structures of all surfaces with intermediate adsorbates were also fully relaxed. All adsorbates are assumed to be neutral species. All these calculations employed a plane wave basis set with 80 Ry and 640 Ry kinetic energy and charge density cutoffs, respectively. Core electrons were described with Vanderbuilt ultrasoft pseudopotentials^42^. A 2×2×1 Γ-centered k-point mesh was employed. The temperature contribution into ΔG was accounted by calculating vibrational contributions (ZPE, H, S) for adsorbates and vibrational, translational and rotational contributions for desorbed species within the ideal gas model, using CP2K code^43^. The consecutive electro-reduction of CO was modelled using Computational Hydrogen Electrode (CHE) approach^39^, employing reversible hydrogen electrode (RHE) as a reference.

### Materials characterization

Fourier transform infrared (FT-IR) spectra were recorded on an Advatar 380 Thermo Nicolet (America) in transmission mode at a resolution of 4 cm^−1^. Elemental analysis was carried out with a CE instruments EA 1110 elemental analyzer (PerkinElmer, America). Thermogravimetric analysis (TGA) was operated on a TA Q500 instrument (America) under nitrogen atmosphere. Powder X-ray diffraction (XRD) patterns were recorded on a Rigaku Ultima IV powder X-ray diffractometer (Japan) (Cu Kα, *λ* = 1.54184 Å) at room temperature. X-ray photoelectron spectroscopy (XPS) spectra were recorded on a Thermo Escalab 250Xi spectrometer (America) using a photon energy of 461 eV with an energy resolution of 0.1 eV. Transmission electron microscopy (TEM) was carried out on a JEM 1400 under 200 KV. The X-ray absorption spectroscopy (XAS) measurements were performed using the QEXAFS configuration on the SuperXAS beamline (at the SLS, Villigen)^44^. The storage ring operated at 2.4 GeV in top-up mode with a ring current of 400 mA, and a focal spot size on the sample position was of 500 × 200 μm(*H*×*V*). The measurements were performed in the traditional transmission geometry using ion-chambers; the data extraction and processing were performed by using ProQEXAFS and the beamline dedicated XAS analysis software^45^. For increased S/N, the data was subsequently averaged over a 600 s total acquisition per sample. Finally, Demeter package (Athena/Artemis) was used for further post-processing and fitting^46^.

### GC analysis

GC was carried out on a FuLi instruments GC9790II with the Ar carrier gas. The thermal conductivity detector (TCD) was used to quantify H_2_ concentration and all the carbon-based products were detected using the flame ionization detector (FID) with a methanizer. The detectors are calibrated by two independent standard gases.

### ^1^H-NMR spectroscopy

^1^H-nuclear magnetic resonance spectroscopy (^1^H-NMR) was performed on a Bruker AC-600 MHz instrument (Switzerland). Typically, 0.5 mL of electrolyte after electrolysis was mixed with 0.1 mL of D_2_O (Sigma-Aldrich, purity: 99.9%) containing 100 ppm dimethyl sulfoxide (DMSO, Sigma-Aldrich, 99.9%) as the internal standard. To ensure the accuracy, both the standard method and external method were employed to calculate the liquid products and the results were exhibited in Supplementary Figs. 27, 28 and Supplementary Table 6.

### Carbon balance

To elucidate the carbon balance path, flow meters were used to monitor the inlet and outlet flow out of the reactor and the results were showed in Supplementary Fig. 29 and Supplementary Table 7.

### In-situ Raman studies

In-situ Raman spectra acquisition was performed using a Renishaw confocal Raman system with the laser excitation at 532 nm. The laser power is kept at 1.5 mW to protect the sample from laser damage. Cu/Cu_2_O-A catalyst was spread on the glassy carbon connecting an external metal bar, which is used as the working electrode that contacts only CO-saturated electrolyte. The other metal bar connecting with a platinum wire extending in the cell was used as the counter electrode. An Ag/AgCl electrode was used as the reference electrode. 2.0 M KOH electrolyte was poured into the cell to immerse these electrodes. The collection time was 10 s and repeated for 3 times for the Cu/Cu_2_O-A sample. The CORR test was performed at the potential of -1.77 V vs Ag/AgCl and a computer synchronously collected the Raman signals.

### ^13^CO electrolysis

The experiment was performed with labeled ^13^CO gas (99.0%, Sigma Aldrich) for electrolysis. The ^13^CO gas flow rate was controlled using a mass flow controller (SevenStar D07-7) at a rate of 20 mL min^−1^. ^13^CO electroreduction was conducted at a constant current of 160 mA cm^−2^ for 10 min and the gas products and the catholyte were collected for analysis by gas chromatography-mass spectrometry (GC-MS).

## Supplementary information

Supplementary Information

## Data Availability

The authors declare that all the relevant data within this paper and its Supplementary Information file are available from the corresponding authors upon a reasonable request. The source data for stability tests of Cu/Cu_2_O-A (Fig. 4b and Supplementary Fig. 11) are provided with this paper. Source data are provided in this paper.

## Source data

Source Data

## References

[1] Chu S, Cui Y, Liu N (2016). The path towards sustainable energy. Nat. Mater..

[2] Lewis NS (2016). Research opportunities to advance solar energy utilization. Science.

[3] Obama B (2017). The irreversible momentum of clean energy. Science.

[4] Wakerley D (2019). Bio-inspired hydrophobicity promotes CO_2_ reduction on a Cu surface. Nat. Mater..

[5] Gao S (2016). Partially oxidized atomic cobalt layers for carbon dioxide electroreduction to liquid fuel. Nature.

[6] Yang HB (2018). Atomically dispersed Ni(i) as the active site for electrochemical CO_2_ reduction. Nat. Energy.

[7] Sutherland BR (2019). Charging up stationary energy storage. Joule.

[8] Zhang X (2020). Molecular engineering of dispersed nickel phthalocyanines on carbon nanotubes for selective CO_2_ reduction. Nat. Energy.

[9] Ma WC (2020). Electrocatalytic reduction of CO_2_ to ethylene and ethanol through hydrogen-assisted C–C coupling over fluorine-modified copper. Nat. Catal..

[10] Chen C (2020). Highly efficient electroreduction of CO_2_ to C_2+_ alcohols on heterogeneous dual active sites. Angew. Chem. Int. Ed..

[11] Huang JNE (2021). CO_2_ electrolysis to multicarbon products in strong acid. Science.

[12] Gao DF, Arán-Ais RM, Jeon HS (2019). & Roldan Cuenya, B. Rational catalyst and electrolyte design for CO_2_ electroreduction towards multicarbon products. Nat. Catal..

[13] Nitopi S (2019). Progress and perspectives of electrochemical CO_2_ reduction on copper in aqueous electrolyte. Chem. Rev..

[14] Chen XY (2021). Electrochemical CO_2_-to-ethylene conversion on polyamine-incorporated Cu electrodes. Nat. Catal..

[15] Ripatti DS, Veltman TR, Kanan MW (2019). Carbon monoxide gas diffusion electrolysis that produces concentrated C_2_ products with high single-pass conversion. Joule.

[16] Pang Y (2019). Efficient electrocatalytic conversion of carbon monoxide to propanol using fragmented copper. Nat. Catal..

[17] Zhuang T-T (2018). Copper nanocavities confine intermediates for efficient electrosynthesis of C_3_ alcohol fuels from carbon monoxide. Nat. Catal..

[18] Chen RX (2020). Highly selective production of ethylene by the electroreduction of carbon monoxide. Angew. Chem. Int. Ed..

[19] Li J (2019). Constraining CO coverage on copper promotes high-efficiency ethylene electroproduction. Nat. Catal..

[20] Jouny M, Luc W, Jiao F (2018). General techno-economic analysis of CO_2_ electrolysis systems. Ind. Eng. Chem. Res..

[21] Li J (2020). Enhanced multi-carbon alcohol electroproduction from CO via modulated hydrogen adsorption. Nat. Commun..

[22] Li CW, Ciston J, Kanan MW (2014). Electroreduction of carbon monoxide to liquid fuel on oxide-derived nanocrystalline copper. Nature.

[23] Han L, Zhou W, Xiang CX (2018). High-rate electrochemical reduction of carbon monoxide to ethylene using Cu-nanoparticle-based gas diffusion electrodes. ACS Energy Lett..

[24] Li J (2019). Effectively increased efficiency for electroreduction of carbon monoxide using supported polycrystalline copper powder electrocatalysts. ACS Catal..

[25] De Luna P (2018). Catalyst electro-redeposition controls morphology and oxidation state for selective carbon dioxide reduction. Nat. Catal..

[26] Lee SY (2018). Mixed copper states in anodized Cu electrocatalyst for stable and selective ethylene production from CO_2_ reduction. J. Am. Chem. Soc..

[27] Li CW, Kanan MW (2012). CO_2_ reduction at low overpotential on Cu electrodes resulting from the reduction of thick Cu_2_O films. J. Am. Chem. Soc..

[28] Xiao H, Goddard WA, Cheng T, Liu YY (2017). Cu metal embedded in oxidized matrix catalyst to promote CO_2_ activation and CO dimerization for electrochemical reduction of CO_2_. Proc. Natl Acad. Sci. USA.

[29] Ren D (2015). Selective Electrochemical reduction of carbon dioxide to ethylene and ethanol on copper(I) oxide catalysts. ACS Catal..

[30] Ling PH, Zhang Q, Cao TT, Gao F (2018). Versatile three-dimensional porous Cu@Cu_2_O aerogel networks as electrocatalysts and mimicking peroxidases. Angew. Chem. Int. Ed..

[31] Dai L (2017). Ultrastable atomic copper nanosheets for selective electrochemical reduction of carbon dioxide. Sci. Adv..

[32] Liu M (2019). Aliphatic amines modified CoO nanoparticles for catalytic oxidation of aromatic hydrocarbon with molecular oxygen. Chin. J. Catal..

[33] Cha MG (2019). Effect of alkylamines on morphology control of silver nanoshells for highly enhanced raman scattering. ACS Appl. Mater. Interfaces.

[34] Wu YA (2019). Facet-dependent active sites of a single Cu_2_O particle photocatalyst for CO_2_ reduction to methanol. Nat. Energy.

[35] Dinh C-Y (2018). CO_2_ electroreduction to ethylene via hydroxide-mediated copper catalysis at an abrupt interface. Science.

[36] Higgins D, Hahn C, C XX, Jaramillo TF, Weber AZ (2019). Gas-diffusion electrodes for carbon dioxide reduction: a new paradigm. ACS Energy Lett..

[37] Gao YG (2020). Cu_2_O nanoparticles with both {100} and {111} facets for enhancing the selectivity and activity of CO_2_ electroreduction to ethylene. Adv. Sci..

[38] Aran-Ais RM (2020). Imaging electrochemically synthesized Cu_2_O cubes and their morphological evolution under conditions relevant to CO_2_ electroreduction. Nat. Commun..

[39] Peterson AA, Abild-Pedersen F, Studt F, Rossmeisl J, Norskov JK (2010). How copper catalyzes the electroreduction of carbon dioxide into hydrocarbon fuels. Energy Environ. Sci..

[40] Perdew JP, Burke K, Ernzerhof M (1996). Generalized gradient approximation made simple. Phys. Rev. Lett..

[41] Giannozzi P (2017). Advanced capabilities for materials modelling with QUANTUM ESPRESSO. J. Phys. Condens. Matter.

[42] Garrity KF, Bennett JW, Rabe KM, Vanderbilt D (2014). Pseudopotentials for high-throughput DFT calculations. Comput. Mater. Sci..

[43] Kuhne TD (2020). CP2K: An electronic structure and molecular dynamics software package - Quickstep: efficient and accurate electronic structure calculations. J. Chem. Phys..

[44] Muller O, Nachtegaal M, Just J, Lutzenkirchen-Hecht D, Frahm R (2016). J. Synchrotron Radiat..

[45] Clark H, Imbao J, Frahm R, Nachtegaal M (2020). J. Synchrotron Radiat..

[46] Ravel B, Newville M (2005). J. Synchrotron Radiat..

