# Supplementary material for: A hydrophobic Cu/Cu2O sheet catalyst for selective electroreduction of CO to ethanol
Source: Nat Commun. 2023 Jan 31;14:501. doi: 10.1038/s41467-023-36261-1 (PMC9889799; doi:10.1038/s41467-023-36261-1)
Supplement: Supplementary file 1 — Supplementary Information [file 41467_2023_36261_MOESM1_ESM.pdf]

Supplementary Information

A hydrophobic Cu/Cu_2O sheet catalyst for selective electroreduction of CO to ethanol

Guifeng Ma,[1] Olga A. Syzgantseva,[2] Yan Huang,[1] Dragos Stoian,[3] Jie Zhang,[3] Shuliang Yang,[4] Wen Luo,[5] Mengying Jiang,[1] Shumu Li,[6] Chunjun Chen,[7,8] Maria A. Syzgantseva,[2] Sen Yan,[1] Ningyu Chen,[1] Li Peng,[1]* Jun Li,[1,9,10]* Buxing Han[7,8]

[1]College of Chemistry and Chemical Engineering, Xiamen University, Xiamen 361005, China. [2]Laboratory of Quantum Photodynamics, Department of Chemistry, Lomonosov Moscow State University, Leninskie gory 1-3, 119991, Moscow, Russia. [3]Institute of Chemical Sciences and Engineering, École Polytechnique Fédérale de Lausanne (EPFL), CH-1951, Sion, Switzerland. [4]College of Energy, Xiamen University, Xiamen, 361102, China. [5]School of Environmental and Chemical Engineering, Shanghai University, 99 Shangda Road, Shanghai 200444, China. [6]Institute of Chemistry, Chinese Academy of Sciences, Beijing Mass Spectrum Center, Beijing 100190, China. [7]Beijing National Laboratory for Molecular Sciences, CAS Key Laboratory of Colloid, Interface and Chemical Thermodynamics, CAS Research/Education Center for Excellence in Molecular Sciences, Institute of Chemistry, Chinese Academy of Sciences, Beijing 100190, P. R. China. [8]University of Chinese Academy of Sciences, Beijing 100049, P. R. China. [9]National Engineering Laboratory for Green Chemical Productions of Alcohols, Ethers and Esters, Xiamen University, Xiamen 361005, China. [10]Collaborative Innovation Center of Chemistry for Energy Materials, Xiamen 361005, China. *E-mail: li.peng@xmu.edu.cn; junnyxm@xmu.edu.cn

Table of contents

Supplementary Figures

Supplementary Fig.1 | XRD and N *1s* patterns of Cu/Cu_2O-A

Supplementary Fig.2 | FTIR spectra for Cu-based catalysts

Supplementary Fig.3 | TGA curves of Cu-based catalysts

Supplementary Fig.4 | Contact angle results for Cu-based catalysts

Supplementary Fig.5 | Normalized XANES and Cu K-edge EXAFS curves for Cu/Cu_2O-A

Supplementary Fig.6 | XRD patterns of Cu-based catalysts

Supplementary Fig.7| Schematic illustration of the flow cell electrolyzer

Supplementary Fig.8 | XPS results for Cu/Cu_2O-A before and after reaction

Supplementary Fig.9 | TEM images for Cu/Cu_2O-A after reaction

Supplementary Fig.10 | SEM images of Cu/Cu_2O-A after test

Supplementary Fig.11| Repeating experiment of stability test for Cu/Cu_2O-A at 164 mA cm^{-2} in 2.0 M KOH

Supplementary Fig.12| In-situ Raman spectra of Cu/Cu_2O-A

Supplementary Fig.13| ^{1}H-NMR spectra of the electrolyte

Supplementary Fig.14| Mass spectrum of ethanol from labeled ^{13}CO electroreduction

Supplementary Fig.15| Mass spectrum of ethylene from labeled ^{13}CO electroreduction

Supplementary Fig.16| Current density of various products for Cu-based catalysts

Supplementary Fig.17| CORR performances for Cu/Cu_2O-X catalysts (X=H, A, S)

Supplementary Fig.18| Faradaic efficiency of the products from CORR on Cu/Cu_2O-H at -0.7 V vs RHE

Supplementary Fig.19| Faradaic efficiency and current density of H_2 for Cu-based catalysts

Supplementary Fig.20| XPS results for Cu-butyl-A before reaction

Supplementary Fig.21| XPS data of Cu *LMM* for the fresh Cu-butyl –A and Cu-butyl-A after test

Supplementary Fig.22| CORR performances for Cu-butyl-X catalysts (X=H, A, S)

Supplementary Fig.23| DFT calculation

Supplementary Fig.24| Simplified pathways for CORR to ethanol

Supplementary Fig.25| Evolution of Gibbs free energy of the oxygen vacancy formation

Supplementary Fig.26| Competitive isoelectronic pathways of CO reduction towards C_2O on the one hand and the oxygen vacancy and water formation on another hand

Supplementary Fig.27| ^{1}H-NMR spectra of standard sample and electrolyte

Supplementary Fig.28| Calibration results for ethanol and acetic acid

Supplementary Fig.29| Carbon balance in KOH electrolyte

Supplementary Tables

Supplementary Table 1| Summary of aqueous CO_2/CO performance on Cu-based electrodes

Supplementary Table 2| CO solubility

Supplementary Table 3| Diffusion coefficients of CO

Supplementary Table 4| Elemental analysis of Cu catalysts

Supplementary Table 5| Energetics of formation of oxygen vacancies on pristine and n-butylamine coated surfaces at U=-0.70 eV

Supplementary Table 6| Summary for the standard samples

Supplementary Table 7| Carbon balance in KOH solution

Supplementarty References

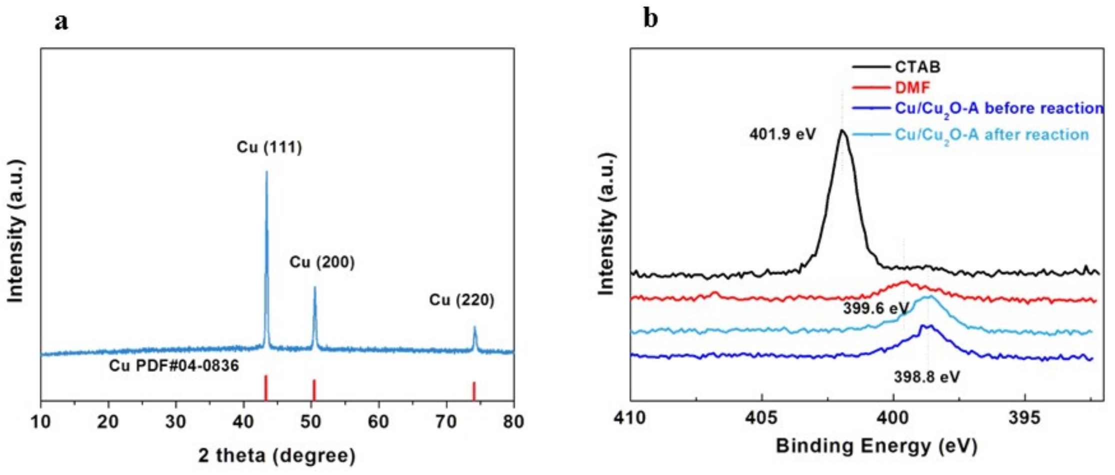

Supplementary Figure 1| XRD and N *1s* patterns of Cu/Cu_2O-A. (a) XRD pattern of the as prepared Cu/Cu_2O-A sample. (b) high-resolution N *1s* spectra.

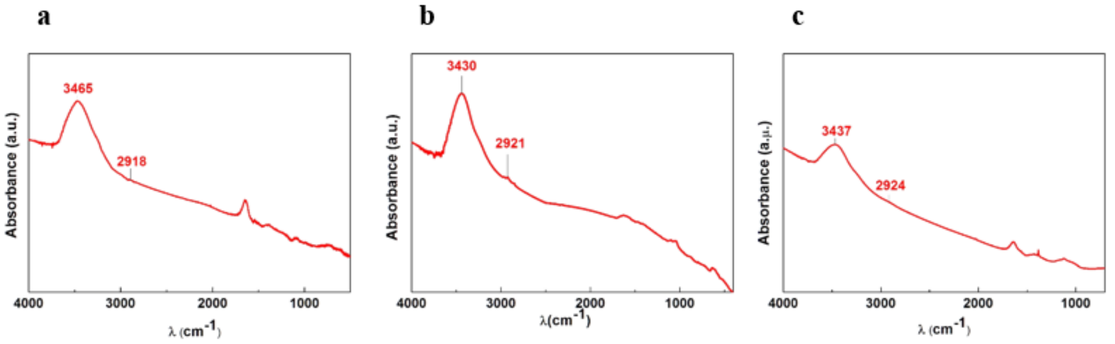

Supplementary Figure 2| FTIR spectra for Cu-based catalysts. (a) Cu/Cu_2O-H, (b) Cu-butyl-A, (c) Cu/Cu_2O-S.

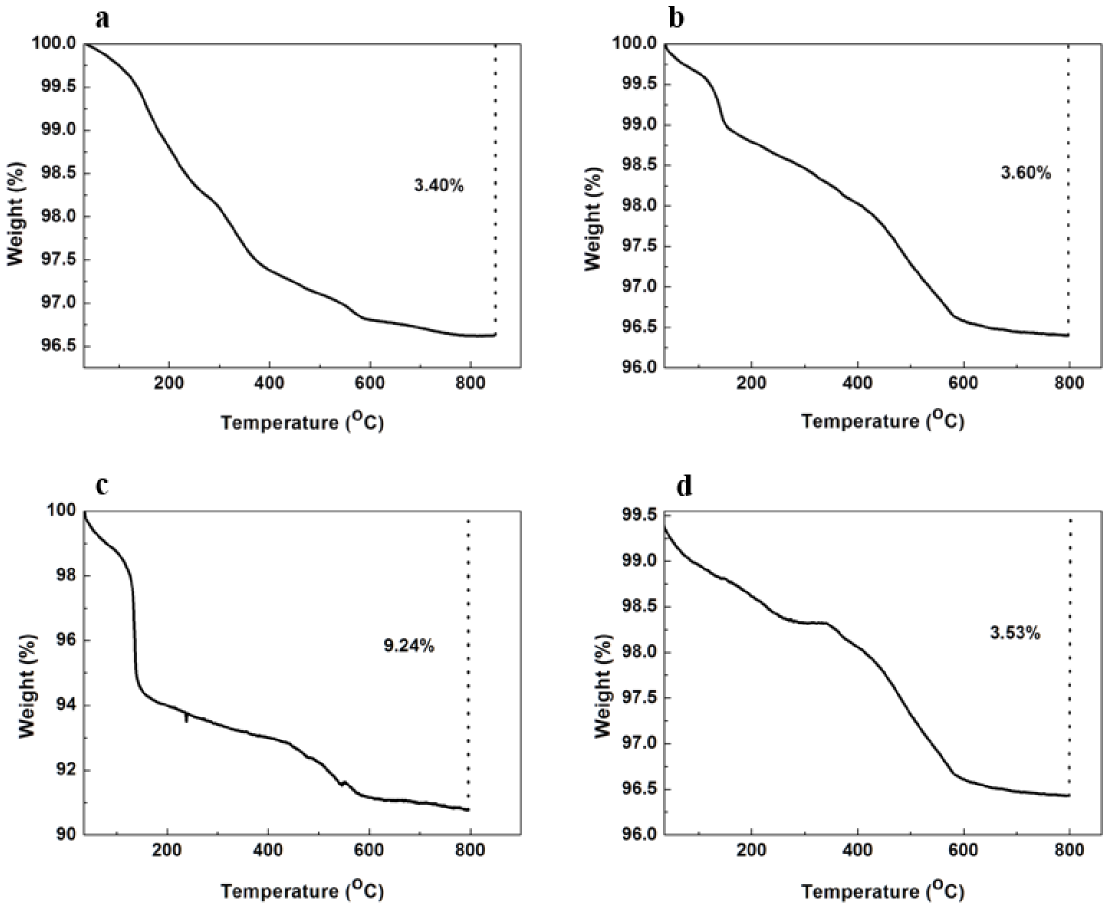

Supplementary Figure 3| TGA curves of Cu-based catalysts. (a) Cu/Cu_2O-H, (b) Cu/Cu_2O-A, (c) Cu/Cu_2O-S, (d) Cu-butyl-A.

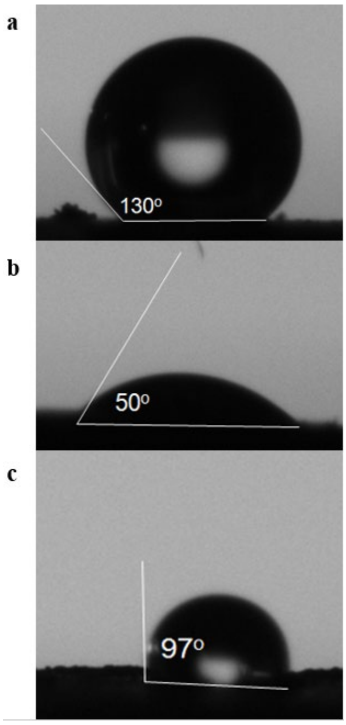

Supplementary Figure 4| Contact angle results for Cu-based catalysts. Contact angle measurements of the Cu/Cu_2O-S (a), Cu/Cu_2O-H (b) and Cu-butyl-A (c).

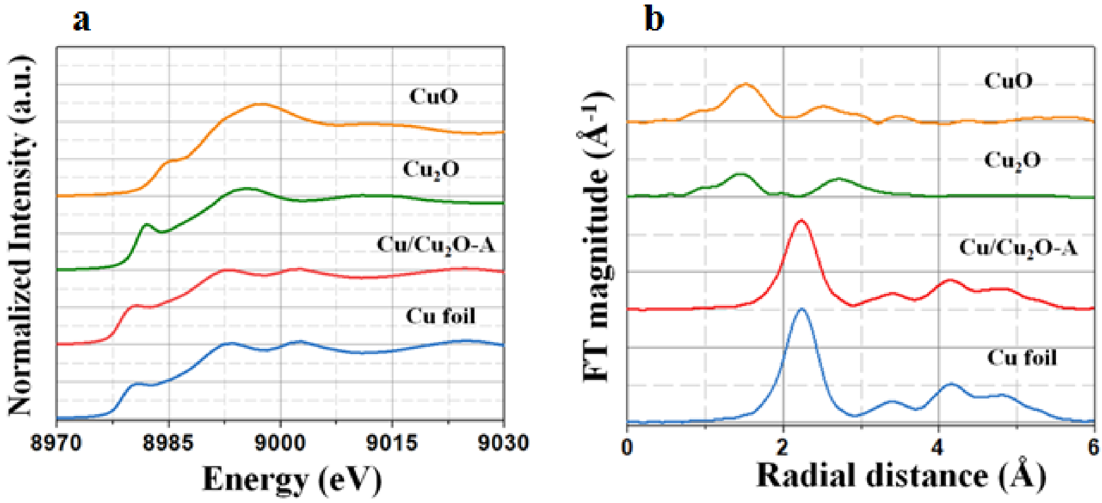

Supplementary Figure 5| Normalized XANES and Cu K-edge EXAFS curves for Cu/Cu_2O-A. Normalized XANES spectra (a) and Fourier transform (FT) of K^2-weighted EXAFS (b) for Cu foil (black), Cu_2O standard (blue), CuO standard (purple) and the as prapared Cu/Cu_2O-A sample (red).

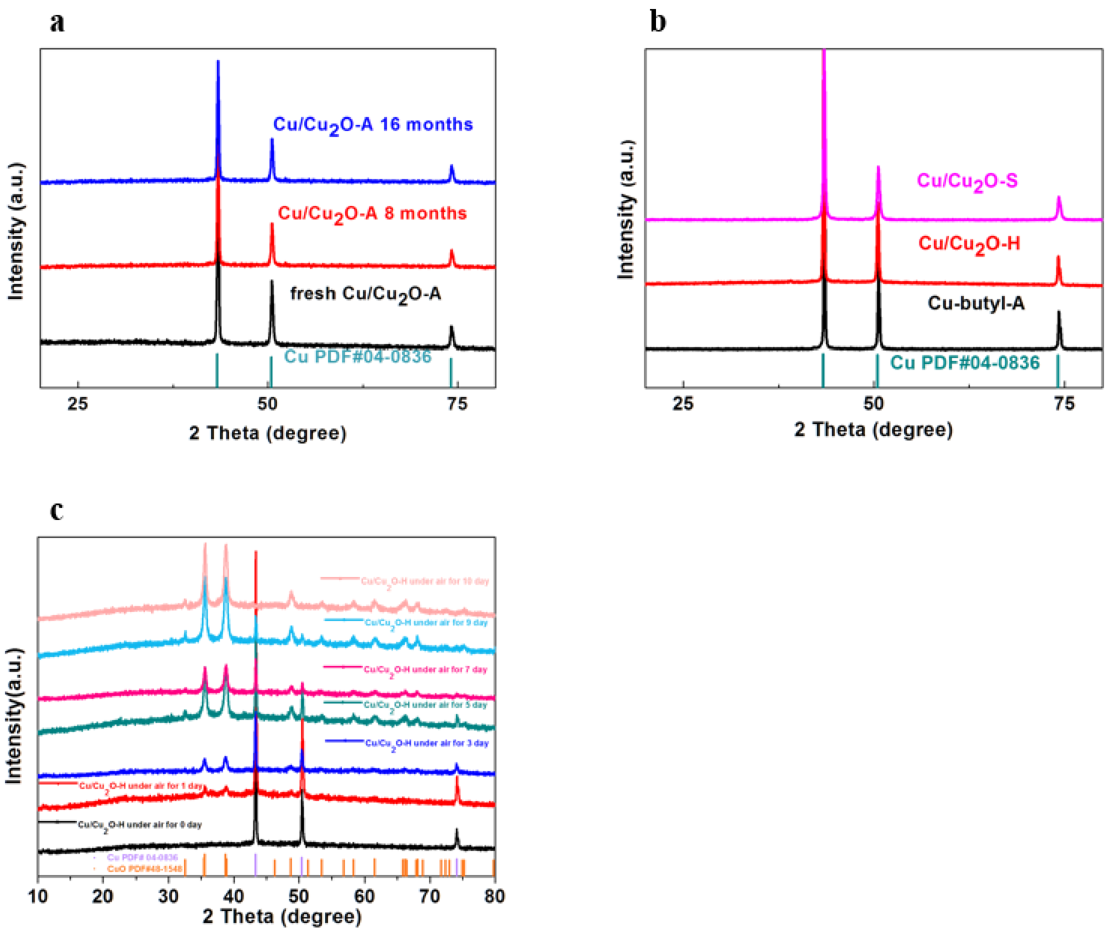

Supplementary Figure 6| XRD patterns of Cu-based catalysts. (a) XRD patterns of Cu/Cu_2O-A after exposed in air at room temperature from fresh to 16 months, indicating the stability of Cu/Cu_2O-A; (b) XRD patterns of Cu/Cu_2O-S, Cu/Cu_2O-H and Cu-butyl-A, manifesting that the modification did not change the structure of the Cu/Cu_2O. The three major peaks in a and b represent (111), (200) and (220), respectively; (c) XRD plots for the Cu/Cu_2O with less n-butylamine for modification under ambient conditions.

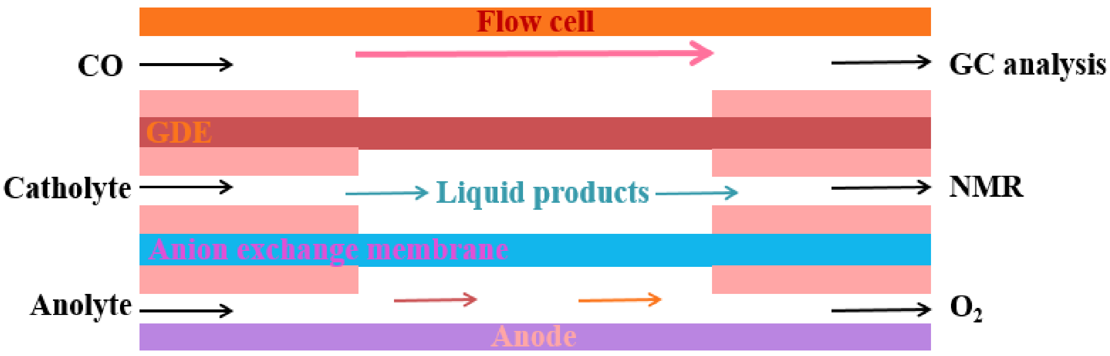

Supplementary Figure 7| Schematic illustration of the flow cell electrolyzer. The orange and blue dotted lines represent the cathode and anode catalysts, respectively.

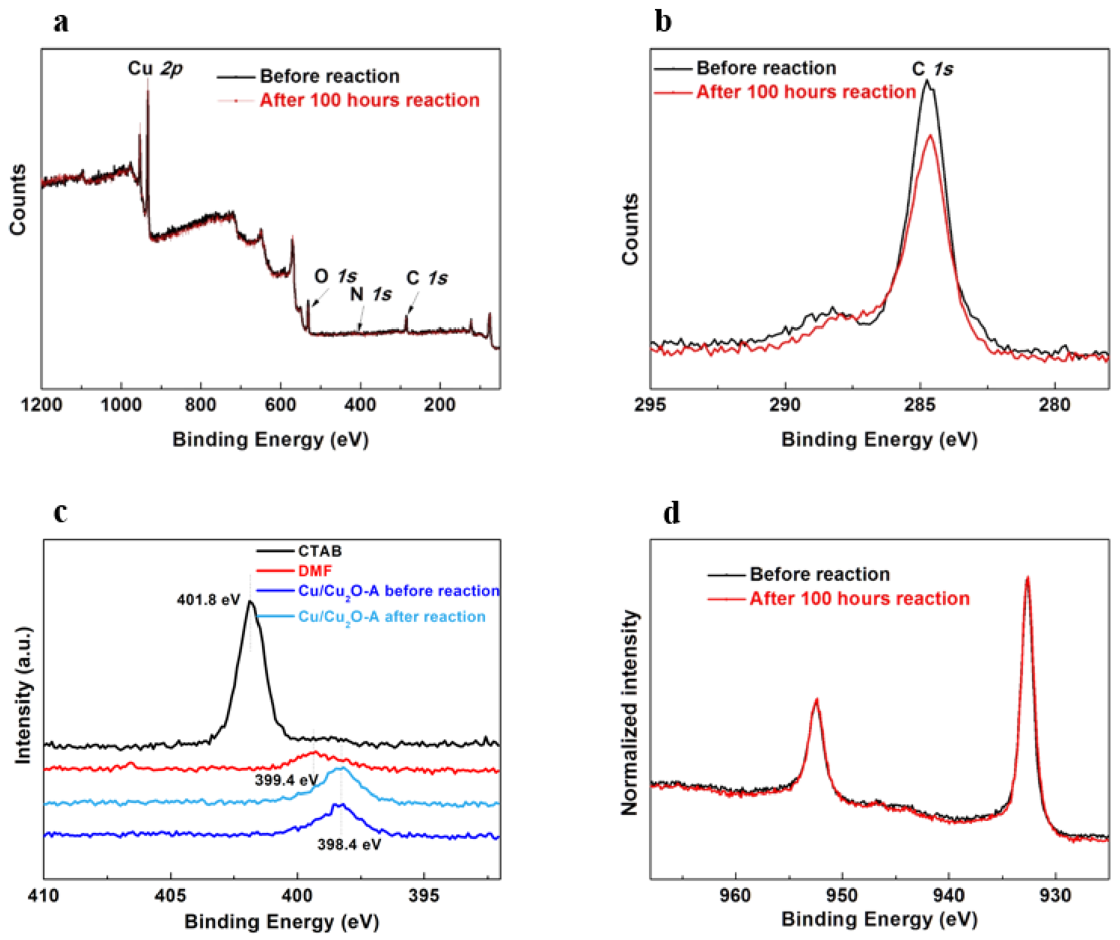

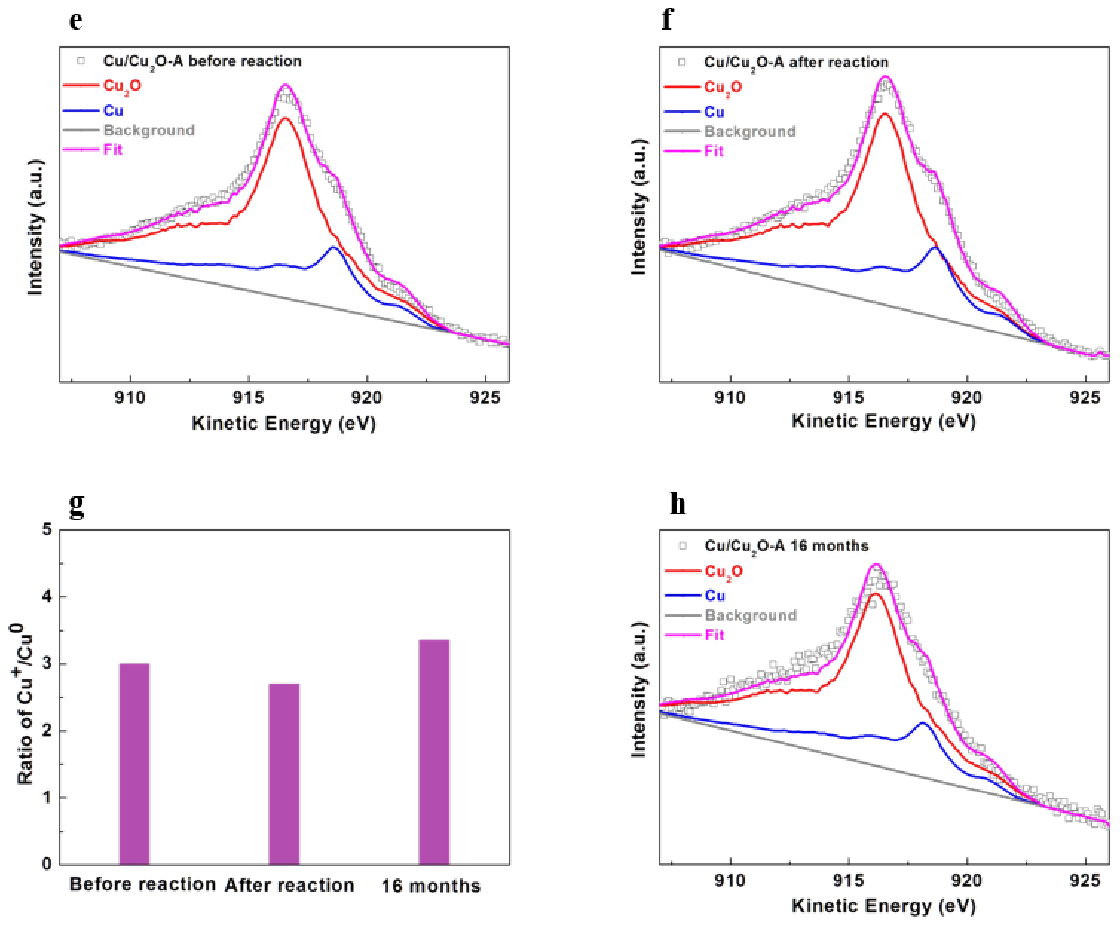

Supplementary Figure 8| XPS results for Cu/Cu_2O-A before and after reaction. XPS spectra of Cu/Cu_2O-A before and after reaction (a-e): (a) survey, (b) high-resolution of C *1s* spectra, (c) high-resolution of N *1s* spectra, (d) Cu *2p*, (e-f) Cu *LMM* of Cu/Cu_2O-A before and after reaction, (g) ratio of Cu^+/Cu^0 over Cu/Cu_2O before and after reaction and Cu/Cu_2O-A after exposed in air at room temperature for 16 months, (h) Cu *LMM* of Cu/Cu_2O-A after exposed in air at room temperature for 16 months.

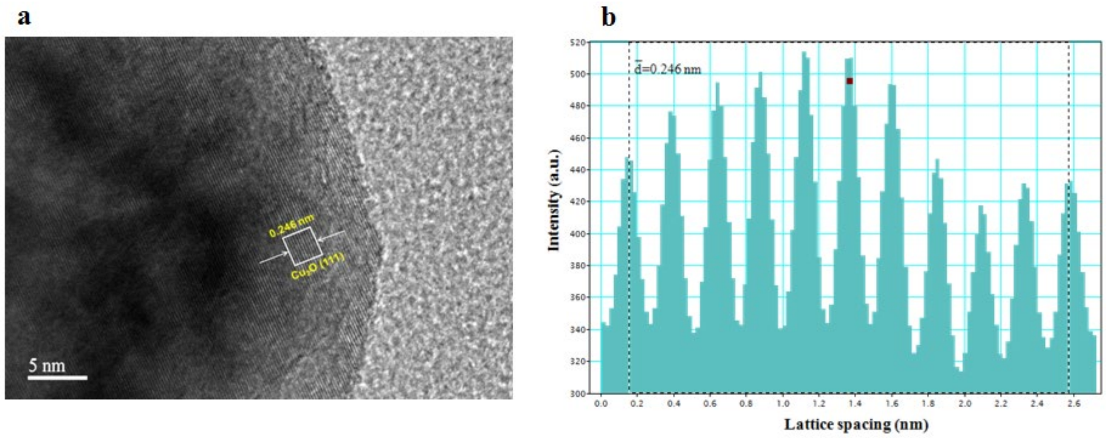

Supplementary Figure 9| TEM images for Cu/Cu_2O-A after reaction. (a) High-resolution transmission electron microscope image of Cu/Cu_2O-A after 100 hours electroreduction performance; (b) Intensity profiles measured from the regions marked by the white squares in image a.

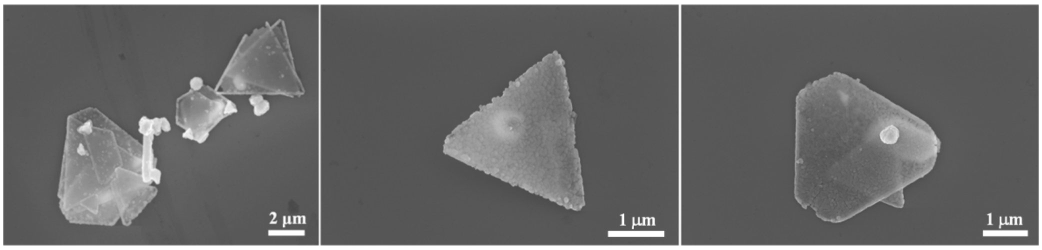

Supplementary Figure 10| SEM images of Cu/Cu_2O-A after test.

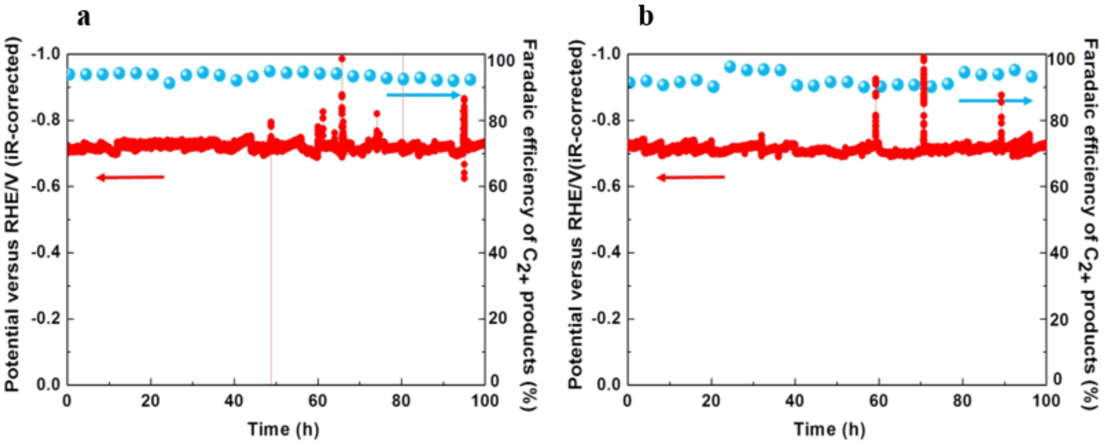

Supplementary Figure 11| Repeating experiment of stability test for Cu/Cu_2O-A at 164 mA cm^{-2} in 2.0 M KOH.

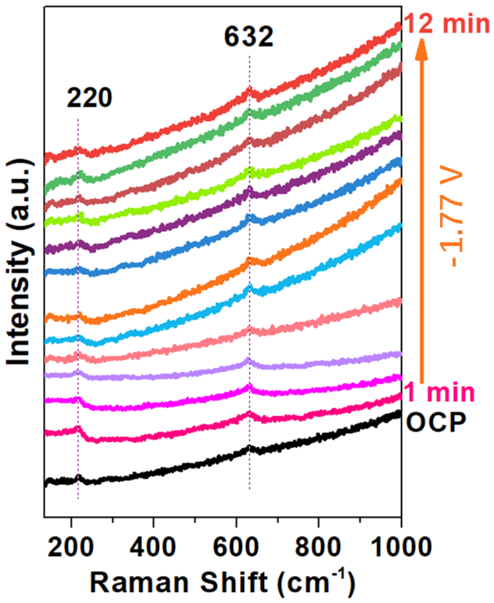

Supplementary Figure 12| In-situ Raman spectra of Cu/Cu_2O-A. In-situ Raman spectra of Cu/Cu_2O-A at open circuit potential (OCP) and -1.77 V vs Ag/AgCl in 2.0 M KOH electrolyte. The in-situ Raman spectra of the catalyst during the reduction with laser excitation at 532 nm were collected in the CO-saturated 2.0 M KOH electrolyte at an applied potential of -1.77 V vs Ag/AgCl. And the Cu/Cu_2O-A catalyst exhibited Raman peaks at 220 and 632 cm^{-1}, which correspond to the 2Γ^{-}_{12} and Γ^{-}_{12} + Γ^{+}_{25} phonon modes, respectively. At the applied potential of -1.77 V vs Ag/AgCl for 12 min, the characteristic peaks for Cu_2O retained, suggesting Cu^{+} is persistent under electrocatalytic conditions.

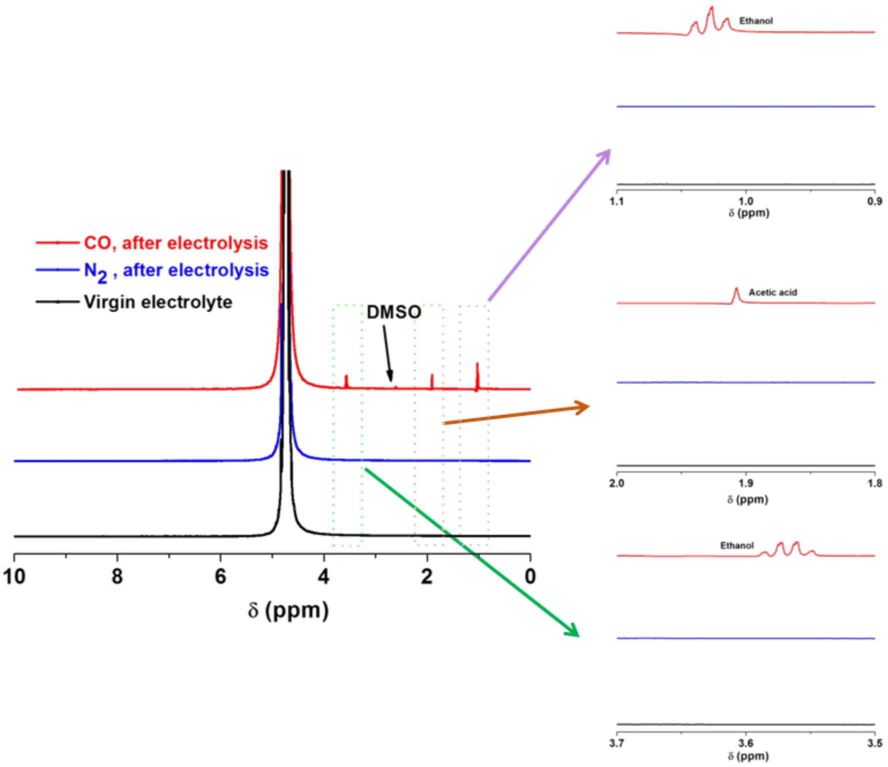

Supplementary Figure 13| ^{1}H-NMR spectra of the electrolyte. Representative ^{1}H NMR spectra of virgin electrolyte (2.0 M KOH aqueous solution, black), N_2-saturated electrolyte (blue) and CO saturated electrolyte after electrolysis for 1 h at -0.7 V vs RHE (red).

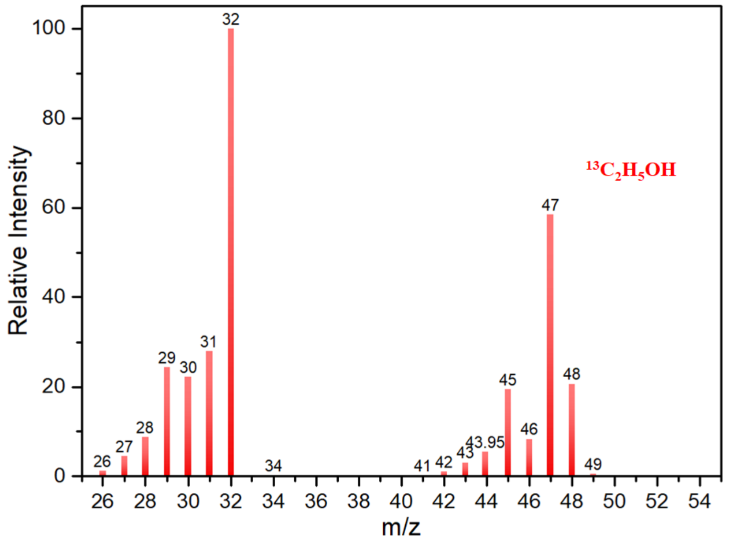

Supplementary Figure 14| Mass spectrum of ethanol from labeled ^{13}CO electroreduction.

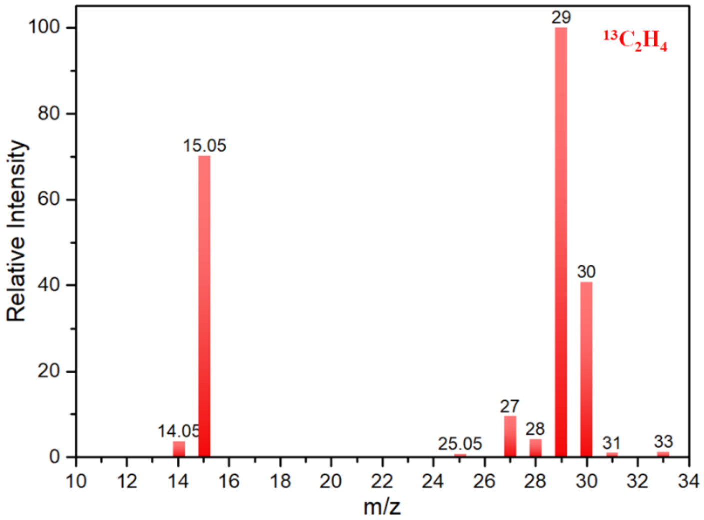

Supplementary Figure 15| Mass spectrum of ethylene from labeled ^{13}CO electroreduction.

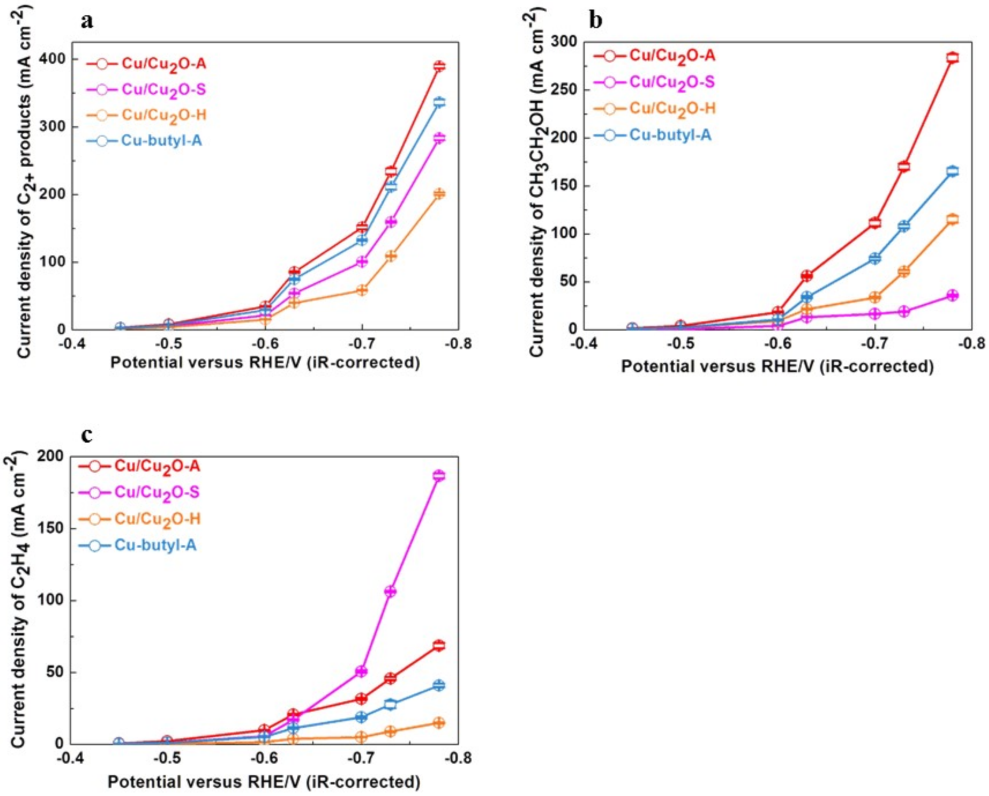

Supplementary Figure 16| Current density of various products for Cu-based catalysts. (a) Current density of C_{2+} products from CORR on Cu/Cu_2O-A, Cu/Cu_2O-S, Cu/Cu_2O-H and Cu-butyl-A. (b) Current density of ethanol for CORR on Cu/Cu_2O-A, Cu/Cu_2O-S, Cu/Cu_2O-H and Cu-butyl-A. (c) Current density of ethylene for CORR on Cu/Cu_2O-A, Cu/Cu_2O-S, Cu/Cu_2O-H and Cu-butyl-A. Error bars represent the standard deviations calculated from three independent measurements.

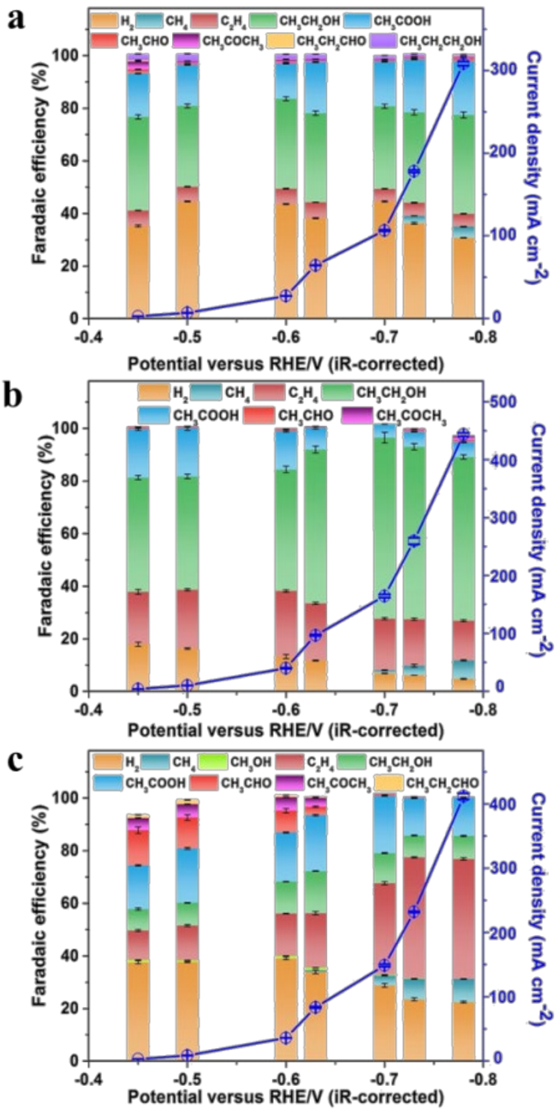

Supplementary Figure 17| CORR performances for Cu/Cu_2O-X catalysts (X=H, A, S). Total current density and Faradaic efficiencies for C_1, C_{2+} products and hydrogen obtained during CORR in 2.0 M KOH versus applied potential for Cu/Cu_2O-H (a), Cu/Cu_2O-A (b), and Cu/Cu_2O-S (c). Error bars represent the standard deviations calculated from three independent measurements.

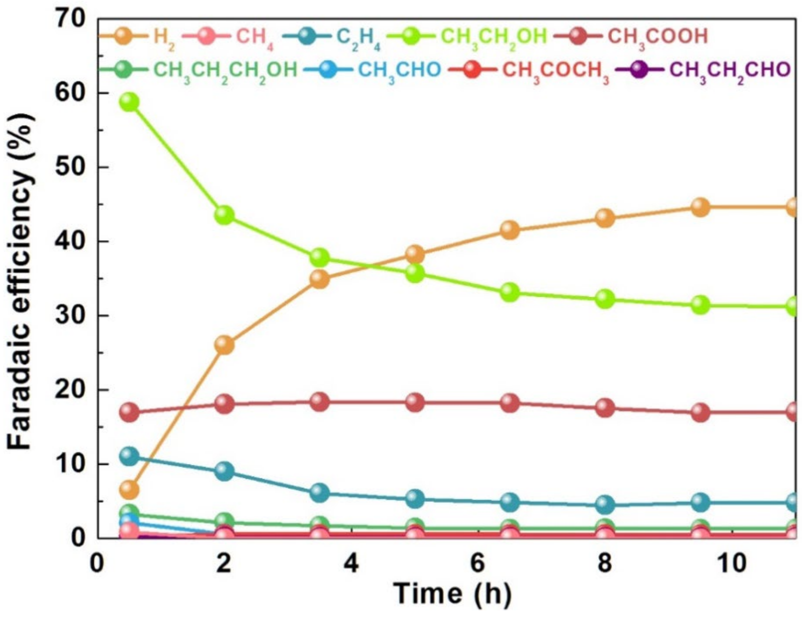

Supplementary Figure 18| Faradaic efficiency of the products from CORR on Cu/Cu_2O-H at -0.7 V vs RHE.

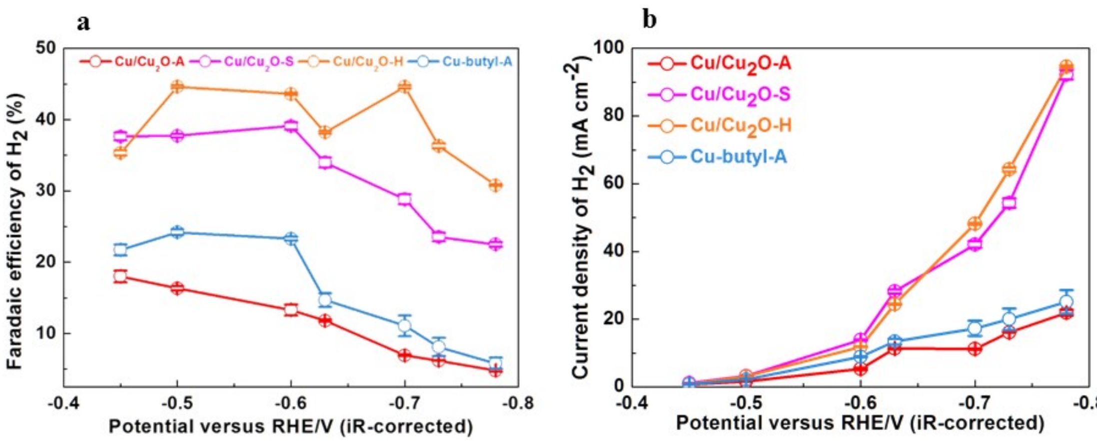

Supplementary Figure 19| Faradaic efficiency and current density of H_2 for Cu-based catalysts. (a) Faradaic efficiency of hydrogen from CORR on Cu/Cu_2O-A, Cu/Cu_2O-S, Cu/Cu_2O-H and Cu-butyl-A. (b) Current density of hydrogen for CO electroreduction on Cu/Cu_2O-A, Cu/Cu_2O-S, Cu/Cu_2O-H and Cu-butyl-A. Error bars represent the standard deviations calculated from three independent measurements.

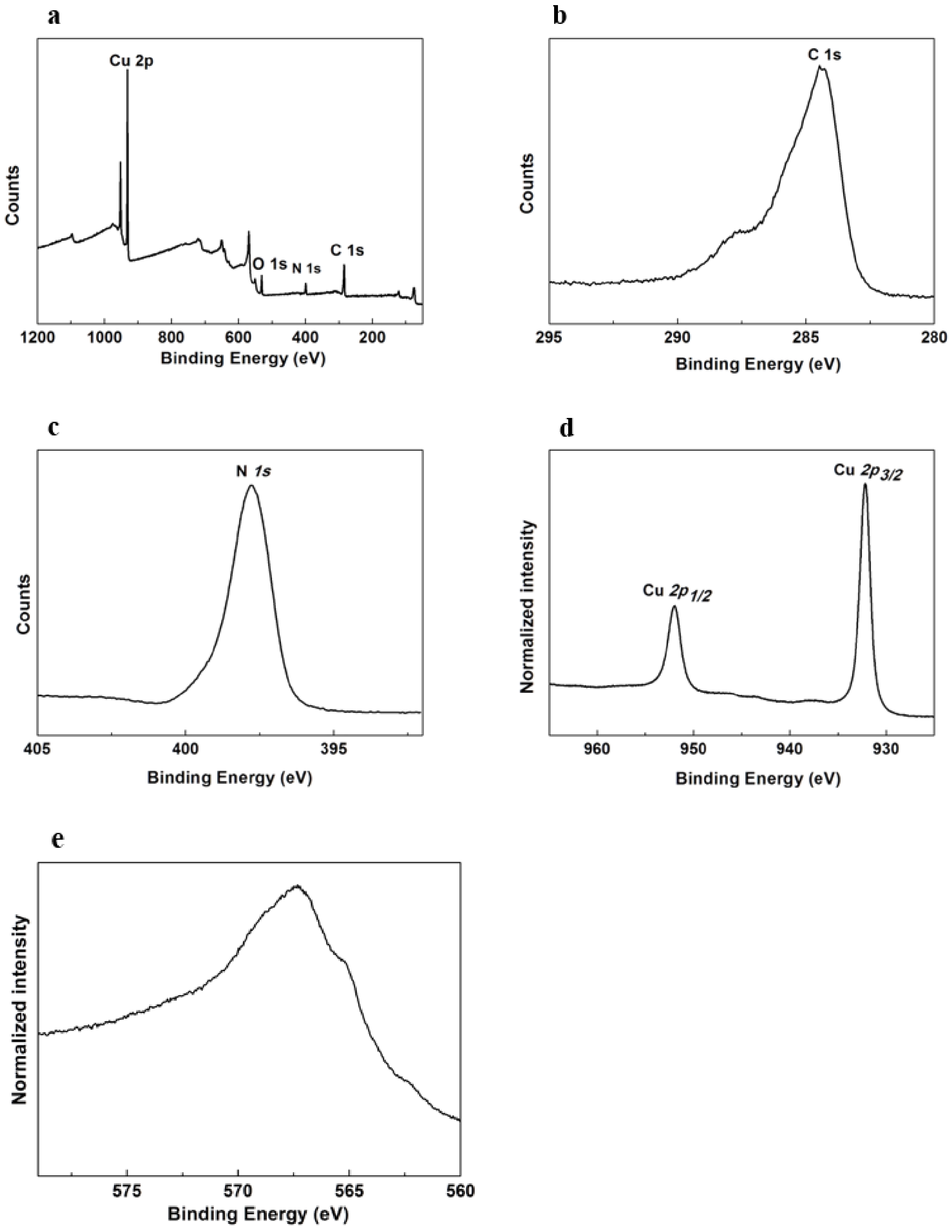

Supplementary Figure 20| XPS results for Cu-butyl-A before reaction. Cu-butyl-A before reaction (a-e): (a) survey, (b) high-resolution of C 1*s* spectra, (c) high-resolution of N 1*s* spectra, (d) Cu 2*p*, (e) Cu *LMM*.

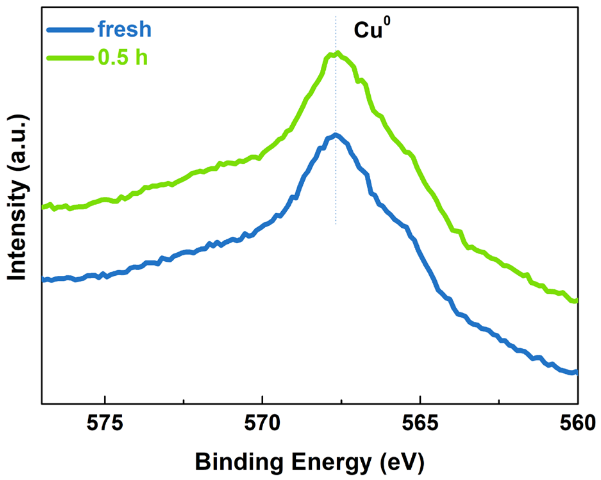

Supplementary Figure 21| XPS data of Cu *LMM* for the fresh Cu-butyl-A and Cu-butyl-A after test.

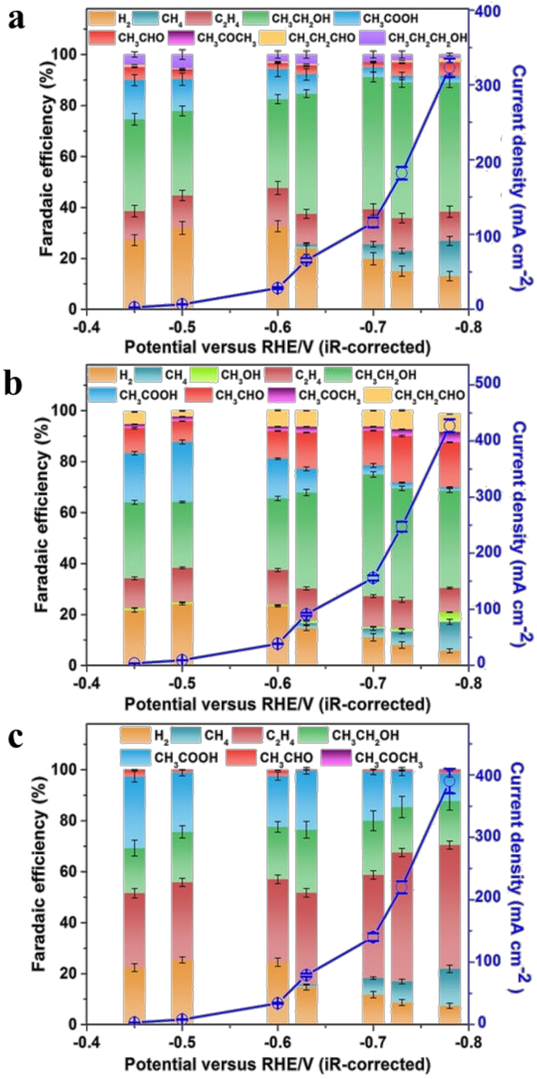

Supplementary Figure 22| CORR performances for Cu-butyl-X catalysts (X=H, A, S). Total current density and Faradaic efficiencies for C_1, C_{2+} products and hydrogen obtained during CORR in 2.0 M KOH versus applied potential for Cu-butyl-H (a), Cu-butyl-A (b), and Cu-butyl-S (c). Error bars represent the standard deviations calculated from three independent measurements.

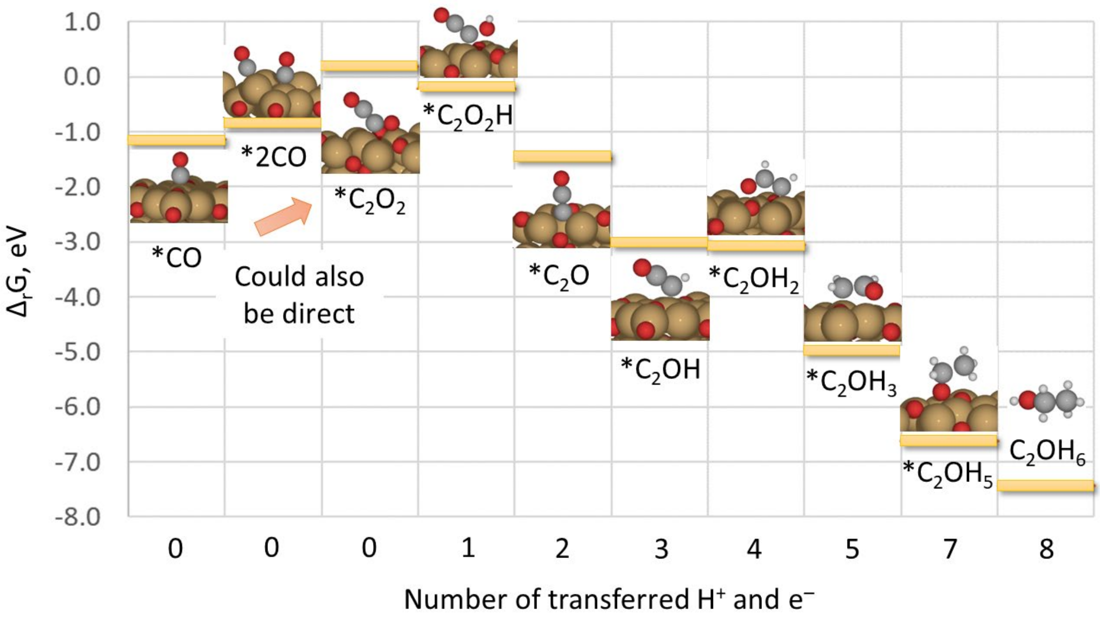

Supplementary Figure 23| DFT calculation. Proposed mechanism for the electroreduction of CO to ethanol and free energy evolution for ethanol formation on Cu_2O (111) at -0.70 V (versus RHE).

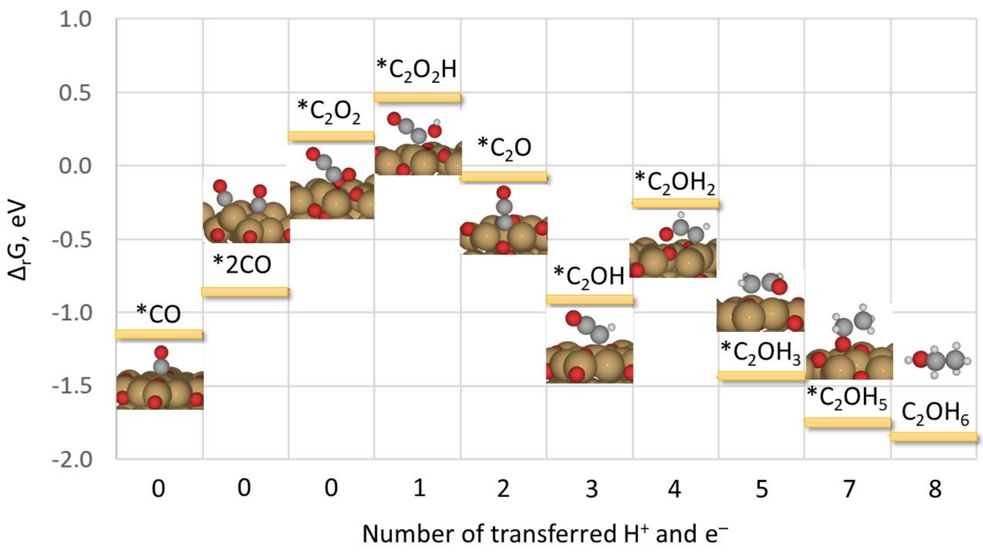

Supplementary Figure 24| Simplified pathways for CORR to ethanol. CO reduction reaction free energy profiles at the potential of 0 V vs RHE, calculated using computational hydrogen electrode model.

The formation of ethanol starts by the C-C coupling step leading to $^*C_2O_2$ species, whose energy is close to the reference (pristine surface and gas phase CO) (Supplementary Figs. 23-24). This is a limiting step of the reaction on (111) surface. For several initial adsorption configurations, a spontaneous dissociation of C_2O_2 into two CO molecules is observed during the simulation. However, if the electron transfer, decoupled from the proton transfer, occurs already at this step, it can be stabilized. Besides, this species can be stabilized by solvent molecules. Therefore, the applied electrode potential of -0.70 V vs RHE ensures the feasibility of ethanol formation with Cu_2O catalyst. All consecutive reaction steps are exergonic ($\Delta_r G < 0$) with gradually decreasing Gibbs free energy. The energy of adsorption on the (111) Cu_2O surface is particularly favorable for the hydrogen-rich intermediates, such as $^*C_2OH$, $^*C_2H_2O$, $^*C_2H_3O$ and $^*C_2H_5O$. In particular, Cu_{cus} sites are important for stabilizing these species through the interaction with non-polar carbon C, to which H atoms are progressively attached. The Cu_{cus}–C distance in these intermediates is relatively short (1.8-1.9 Å), suggesting a strong interaction between the adsorbate and the surface. Besides, the Cu_{cus} site participates in the stabilization of $^*C_2H_5O$ adsorption via O atom. Therefore, the appropriate surface structure of the employed electrocatalyst predetermines its efficiency in the ethanol formation reaction.

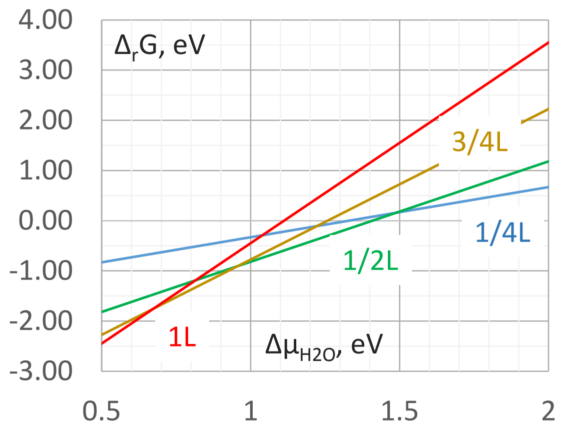

Supplementary Figure 25| Evolution of Gibbs free energy of the oxygen vacancy formation. Evolution of Gibbs free energy of the oxygen vacancy formation reaction as a function of water chemical potential variation within the n-butylamine layer.

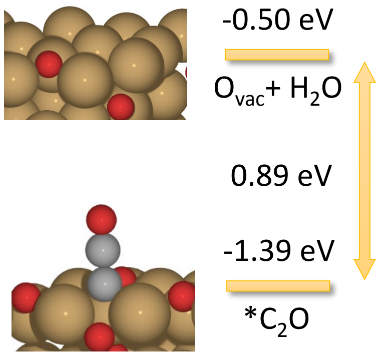

Supplementary Figure 26| Competitive isoelectronic pathways of CO reduction towards C_2O on the one hand and the oxygen vacancy and water formation on another hand.

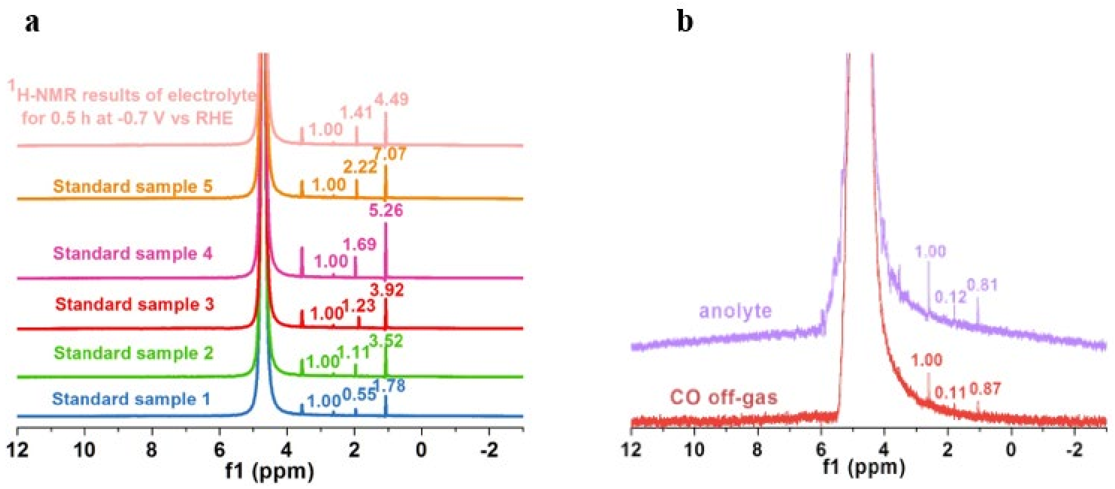

Supplementary Figure 27| ^{1}H-NMR spectra of standard sample and electrolyte. (a) ^{1}H-NMR spectra of standard sample 1-5 and the electrolyte after CORR on Cu/Cu_2O-A cathodes for 0.5 h at -0.7 V versus RHE; (b) ^{1}H-NMR spectra of products in CO off-gas and anolyte for 0.5 h at -0.7 V versus RHE.

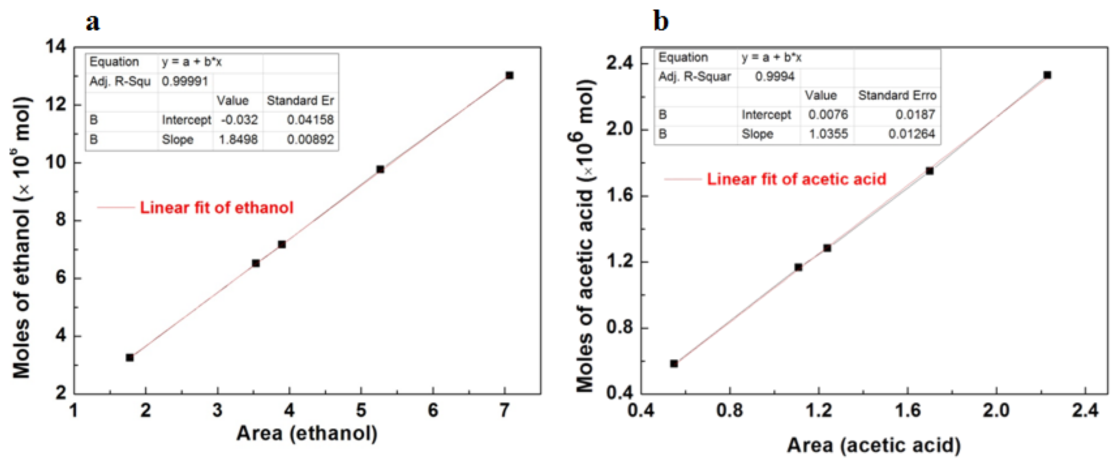

Supplementary Figure 28| Calibration results for ethanol and acetic acid. Calibration curve for ethanol concentration (a) and acetic acid concentration (b) vs ^{1}H-NMR.

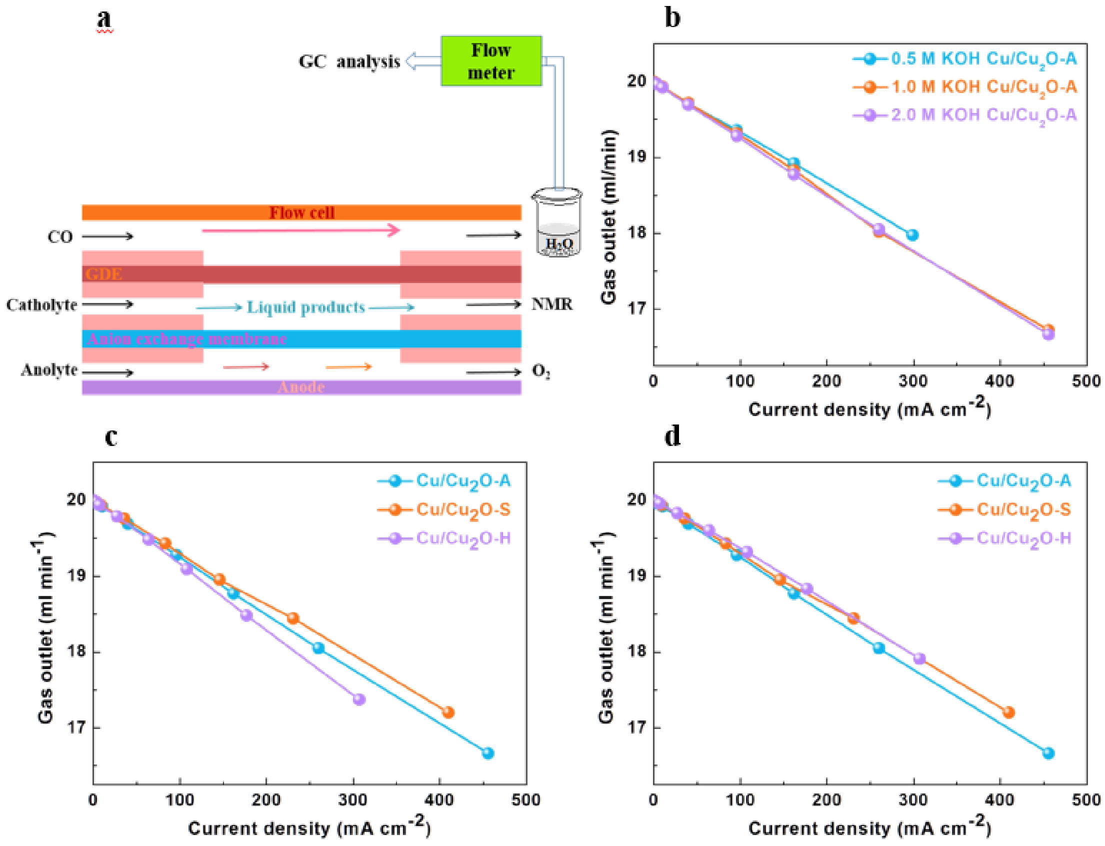

Supplementary Figure 29| Carbon balance in KOH electrolyte. (a) Schematic illustration of the flow cell setup for CORR, (b) Gas outlet flowrates from flow meter after CORR for Cu/Cu2O-A in 0.5-2.0 M KOH electrolyte, (c) Gas outlet flowrates from flow meter after CORR for Cu/Cu2O-A, Cu/Cu2O-S and Cu/Cu2O-H in 2.0 M KOH electrolyte, (d) Gas outlet flowrates from flow meter after CORR for Cu-butyl-A, Cu-butyl-S and Cu-butyl-H in 2.0 M KOH.

Supplementary Table 1| Summary of aqueous CO_2/CO performance on Cu-based electrodes.

Electrolyte	Potential (V versus RHE)	C_{2+} product current density ($mAcm^{-2}$)	C_{2+} product Faradaic Efficiency (%)	Ethanol Faradaic Efficiency (%)	References
			CO RR		

2.0 M KOH	-0.67	635	90.7	26.7	[1]
1.0 M KOH	-0.72	829	79.0	19.9	
0.1 M KOH	-0.30	0.16	56.5	42.9	[2]
1.0 M KOH	-0.70	15.96	72.5	7.4	[3]
0.1 M KOH	-0.30	N/A	72	35.0	[4]
0.1 M KOH	-0.59	1.19	56.2	11.2	[5]
0.1 M KOH	-0.30	0.144	65.43	49.79	[6]
2.0 M KOH	-0.736	137.4	68.7	2.4	[7]
0.1 M KOH	-0.23	0.21	98.85	15.38	[8]
1.0 M KOH	-0.56	NA	62.3	12.5	[9]
1.0 M KOH	-0.66	NA	82.0	16.0	[10]
1.0 M KOH	-0.52	113.6	94.0	20.0	[11]
1.0 M KOH	-0.46	10.8	79.2	13.4	[12]
1.0 M KOH	-0.47	39.0	80.8	17.4	[13]
0.1 M KOH	-0.40	0.40	48.9	26.0	[14]
1.0 M KOH	-0.62	641	91.4	33.2	[15]
**2.0 M KOH**	**-0.70**	**151**	**93.5**	**68.8**	**This work**
**1.0 M KOH**	**-0.76**	**136**	**84.2**	**55.3**	
			**CO_2 RR**		
1.0 M $KHCO_3$	-0.52	NA	64.4	20.6	[16]
1.0 M KOH	-0.68	264	85.1	25.9	[17]
3.5 M KOH	-0.66	607	82	~10	[18]
0.1 M $KHCO_3$	-1.1	NA	46.4	3.7	[19]
0.1 M $KHCO_3$	-1.0	24.8	73.0	24.0	[20]
0.1 M $KHCO_3$	-0.9	12.0	60.0	0	[21]
0.1 M $KHCO_3$	-1.2	2.4	63	63.0	[22]
0.1 M $KHCO_3$	-1.2	31	52.5	7.8	[23]
0.1 M $KHCO_3$	-1.03	18.7	59.8	11.8	[24]
0.25 M $KHCO_3$	-0.96	41.2	60.5	11	[25]
0.1 M $KHCO_3$	-1.0	2.9	57.8	9.7	[26]
0.1 M $KHCO_3$	-0.99	17.8	50.8	17.22	[27]
0.5 M $KHCO_3$	-0.8	11	55.0	0	[28]
0.1 M $CsHCO_3$	-1.6	22.2	74.0	17.0	[29]
0.1 M $KHCO_3$	-1.00	NA	52.3	11.1	[30]
0.1 M KCl	-0.40	9.28	80.7	32.2	[31]
0.5 M $KHCO_3$	-1.10	22.0	59.0	0	[32]
2.0 M KOH	-0.60	267.0	75.2	26.9	[33]

0.5 M $KHCO_3$	-0.50	NA	64.4	28.9	[34]
1.0 M KOH	-0.80	225.7	84.3	22.9	[35]
1.0 M KOH	-0.70	240.9	80.3	42.6	[36]
1.0 M $KHCO_3$	-0.46	264.9	88.0	41.2	[37]
7.0 M KOH	-0.67	280.0	90.0	10.0	[38]
10.0 M KOH	-0.47	46.0	60.0	0	[39]
0.1 M $KHCO_3$	-1.60	180	60.0	0	[40]
0.1 M $KHCO_3$	-1.10	35.9	78.0	16.0	[41]
1.0 M KOH	-0.58	97.4	48.7	11.2	[42]
1.0 M KOH	-0.67	411.0	62.0	16.6	[43]
0.1 M $KHCO_3$	-1.0	NA	86.0	8.0	[44]
0.1 M $KHCO_3$	-0.86	11.3	55.2	16.6	[45]
0.1 M $KHCO_3$	-1.0	NA	76.0	32.0	[46]
1.0 M KOH	-0.68	156	93.0	52.0	[47]
0.75 M KOH	-0.89	1044	80	12.0	[48]
1.0M KOH	-0.75	325	81.3	43.0	[49]
0.1M $CsHCO_3$	-1.15	10.4	80	46	[50]
0.1M $KHCO_3$	-1.1	20.6	73	51	[51]
1.0 M KOH	-0.87	640	80	40.8	[52]
1.0M KOH	-1.2	5.9	65	32.5	[53]

COSMO-RS calculation

In this work, BP functional combined with def2-TZVPD basis set was employed to carry out the quantum chemical COSMO calculations for CO, water and n-butylamine molecules. After obtaining their COSMO files, COSMO-RS calculations were subsequently performed using COSMOtherm C30_1601 program [54, 55] to evaluate the macroscopic solubility of CO in water and n-butylamine respectively.

Supplementary Table 2| CO solubility. CO solubility (mole fraction) in water and n-butylamine at 298.15 K and 101.325 kPa calculated by COSMO-RS theory

Temperature (K)	Water	n-Butylamine

298.15	1.72×10^{-5}	2.31×10^{-3}
303.15	1.79×10^{-5}	2.26×10^{-3}
308.15	1.87×10^{-5}	2.20×10^{-3}

Molecular dynamics simulations

Molecular dynamics simulations were performed to obtain the diffusion coefficients of CO in water and n-butylamine with Gromacs 2019.6 program package [56]. 10 CO molecules and 560 water or n-butylamine molecules were packed into the simulation boxes using Packmol program [57]; it is worth mentioning that the system size has been reported to be enough to gain the reliable CO diffusion coefficients in the molecular solvents [58]. The water molecule adopted the SPC/E (Extended Simple Point Charge) model and the n-butylamine molecule used the classical GAFF force field [59], whereas the parameters of CO were taken from the literature [58]. The restrained electrostatic potential (RESP) method was employed to get partial charges of the systems. The initial systems were energetically minimized with the convergence criteria of 100 kJ·mol^{-1}/nm. Following that, NPT ensembles were used to perform 80 ns simulations to make the systems verge to equilibrium. The temperature was set at 298.15 K with the velocity-rescale heat bath [60], while the pressure was controlled by Berendsen algorithm for the former 30 ns and Parrinello-Rahman scheme for the last 50 ns [61, 62]. The long-range coulomb interactions were calculated by particle mesh Ewald (PME) method [63]. The LINCS algorithm [64] was used to constrain all the bonds connecting with hydrogen. The equations of motion were integrated by the leap-frog algorithm and the time step was set to 2 fs. After the simulation boxes equilibrium reached, the last frame of simulation trajectory was chosen as the initial configuration to carry out another 50 ns NVT production simulation for the diffusion coefficient calculations.

Supplementary Table 3| Diffusion coefficients of CO. Diffusion coefficients of CO in water and n-butylamine at 298.15 K calculated by molecular dynamics simulations

**Systems**	***D*** **(m^2/s)**

Water	2.33×10^{-9}
n-Butylamine	2.54×10^{-9}

Supplementary Table 4| Elemental analysis of Cu catalysts.

Samples	C (%)	N (%)
Cu/Cu_2O-H	2.012	0.532
Cu/Cu_2O-A (before reaction)	2.136	0.680
Cu/Cu_2O-A (after 100 hours reaction)	2.108	0.671
Cu/Cu_2O-S	5.776	1.672
Cu-butyl-A	2.098	0.668

Supplementary Table 5. Energetics of formation of oxygen vacancies on pristine and n-butylamine coated surfaces at U=-0.70 eV (1/4 layer coverage).

Pristine		n-butylamine coated	
Wrt O_2 (1)	Wrt H_2O (2)	Wrt O_2 (1)	Wrt H_2O (2)
1.92	-0.50	2.37	-0.05

The survival of Cu_2O implies that the departure of oxygen atoms from the Cu_2O surface is energetically hindered. Our DFT calculations demonstrate that, in fact, the formation of oxygen vacancies is energetically impeded on the n-butylamine-coated Cu_2O surface, as compared to the pristine one (Supplementary Table 5), according to both reactions:

$$Cu_2O = Cu_2O_{vac} + {}^{1}/_{2}\, O_2 \qquad (1)$$

$$Cu_2O + 2(H^+ + e^-) = Cu_2O_{vac} + H_2O \qquad (2)$$

Besides, the presence of hydrophobic n-butylamine on the surface increases the chemical potential of water. This happens both due to the increase of the internal energy of water (*E*) within the hydrophobic layer originating from the unfavorable hydrophobic interactions between the water molecules and hydrocarbon chains of n-butylamine and due to the restriction of the phase space (*Q*), accessible for water molecules within the hydrophobic environment of n-butylamine coating (hydrophobic n-butylamine repels water molecules):

$$\mu_{H_2O} = E_{H_2O} - RTlnQ \qquad (3)$$

where μ, E, Q stand for chemical potential, electronic energy and sum over states,

respectively.

As a consequence, the formation of oxygen vacancies, and thus departure of O atoms and reduction of the Cu_2O towards the pristine Cu becomes even more unfavorable, since water molecules being able to approach the Cu_2O surface is a prerequisite of the oxygen vacancy formation reaction taking place (in fact, direct reaction $Cu_2O = Cu_2O_{vac} + {}^1/_2\,O_2$ is much more unfavorable than the water-mediated one). The corresponding Gibbs free energy diagrams are demonstrated in the Supplementary Fig.25.

Another factor that plays a role here is that the oxygen vacancy formation process is less energetically favorable than the isoelectronic process of CO reduction at the C2O formation stage, when 2 electrons are transferred from the surface (Supplementary Fig.26). Thus, under the reaction conditions oxygen vacancy formation is not competitive with the CO coupling and reduction process.

All these factors make the departure of oxygen atoms and destruction of Cu_2O phase unfavorable in the presence of n-butylamine.

The detailed calculation of Faradaic efficiency is as below:

Double standard method: Firstly, a series of standard ethanol and acetic acid solutions were prepared as shown in Supplementary Table 6. The ^{1}H-NMR peak areas of ethanol, acetic acid and DMSO are proportional to their moles, so we defined the correction factor $f = \frac{A_S/n_s}{A_r/n_r}$, where A_s and A_r are the areas of the internal standard (DMSO) and standard product (ethanol or acetic acid), respectively; n_s and n_r are the amounts (in mol) of the internal standard (DMSO) and standard product (ethanol or acetic acid), respectively. As shown in Table 6, the average values of $f_{ethanol}$ and $f_{acetic\ acid}$ are 14.383 and 8.183, respectively. For the electrolyte reacted for 0.5 h at the potential of -0.7 V vs RHE (Supplementary Figure 27 and Supplementary Table 6), the areas of ethanol and acetic acid are 4.49 and 1.41, respectively. Thus the calculated amounts corresponding to ethanol and acetic acid in 0.5 mL electrolyte are 8.266×10^{-6} mol and 1.477×10^{-6} mol, respectively. The total amount of ethanol in the catholyte (30 mL) is 4.960×10^{-4} mol, and the total amount of acetic acid in the catholyte is 8.862×10^{-5} mol. All the samples were measured at least three times and

the amount of ethanol is calculated to be 4.946 × 10^{-4} mol, 4.911 × 10^{-4} mol, 4.960 × 10^{-4} mol and the amount of acetic acid is calculated to be 6.125 × 10^{-5} mol, 7.198 × 10^{-5} mol, 8.862 × 10^{-5} mol at -0.7 V versus RHE and 2.0 M KOH electrolyte, respectively. So the average of the amount of ethanol and acetic acid in catholyte are 4.939 × 10^{-4} mol and 7.395 × 10^{-5} mol, respectively.

External method can also be employed to calculate the FEs of the liquid products. As shown in Supplementary Table 6 and Supplementary Figure 28, the NMR peak areas of standard samples are proportional to their mole numbers. According to the linear fit of ethanol and acetic acid, the mole numbers of ethanol and acetic acid in 0.5 mL electrolyte are 8.280×10^{-6} mol and 1.474×10^{-6} mol, respectively. The total amount of ethanol in the catholyte (30 mL) is 4.968×10^{-4} mol, and the total amount of acetic acid in the catholyte is 8.844×10^{-5} mol. These results are consistent with that calculated by double standard method.

For the liquid products in the CO off-gas, the NMR peak areas of the liquid products also are proportional to their mole numbers. According to the areas ratio of the internal standard (DMSO) to ethanol and acetic acid, the mole ratio of ethanol and acetic acid to DMSO in 0.5 mL washing solution are 1.74 and 0.22, respectively ($\frac{S_{ethanol}}{S_{DMSO}} \times \frac{6}{3} = \frac{0.87}{1.00} \times \frac{6}{3} = \frac{n_{ethanol}}{n_{DMSO}} = 1.74$, $\frac{S_{acetic\ acid}}{S_{DMSO}} \times \frac{6}{3} = \frac{0.11}{1.00} \times \frac{6}{3} = \frac{n_{acetic\ acid}}{n_{DMSO}} = 0.22$). As the mole numbers of DMSO in 0.1 mL D_2O is 1.28 × 10^{-7} mol, so the mole numbers of ethanol and acetic acid in 0.5 mL washing solution are 2.23 × 10^{-7} and 2.82 × 10^{-8} mol, respectively. So the total ethanol and acetic acid in 30 mL washing solution are 1.338 × 10^{-5} and 1.692 × 10^{-6} mol, respectively. All the samples were measured at least three times and the average amount of ethanol is calculated to be 1.337 × 10^{-5} mol (based on three measurements: 1.338 × 10^{-5} mol, 1.178 × 10^{-5} mol, 1.496 × 10^{-5} mol) and the average amount of acetic acid is calculated to be 1.623 × 10^{-6} mol (based on three measurements: 1.692 × 10^{-6} mol, 1.361 × 10^{-6} mol, 1.815 × 10^{-6} mol) obtain from the the CO off-gas liquid at -0.7 V versus RHE and 2.0 M KOH electrolyte, respectively.

The same calculation methods as above can also be applied for the calculation of amount of ethanol and acetic acid in the anolyte, thus accoding to Figure S27b the mole number of ethanol in anolyte is calculated to be 1.246 × 10^{-5} mol, the mole number of acetic acid in anolyte is calculated to be 1.815 × 10^{-6} mol. All the samples

were measured at least three times and the average amount of ethanol is calculated to be 1.239×10^{-5} mol (based on three measurements: 1.246×10^{-5} mol, 1.209×10^{-5} mol, 1.262×10^{-5} mol) and the average amount of acetic acid is calculated to be 1.815×10^{-6} mol (based on three measurements: 1.815×10^{-6} mol, 1.512×10^{-6} mol , 2.117×10^{-6} mol) obtain from the anolyte at -0.7 V versus RHE and 2.0 M KOH electrolyte, respectively.

Based on the above results, the total mole number of ethanol at -0.7 V versus RHE and 2.0 M KOH electrolyte is 5.197×10^{-4} mol (4.939×10^{-4} mol + 1.337×10^{-5} mol + 1.239×10^{-5} mol = 5.197×10^{-4} mol) and the total mole number of acetic acid at -0.7 V versus RHE and 2.0 M KOH electrolyte is 7.739×10^{-5} mol (7.395×10^{-5} mol + 1.623×10^{-6} mol + 1.815×10^{-6} mol =7.739×10^{-5} mol). The overall charge for the reaction is 2×0.162×3600×0.5=583.2 C in 0.5 h, where the charge is $5.197\times10^{-4}\times8\times96485=401.1$ C for the production of ethanol and it is $7.739\times10^{-5}\times4\times96485=29.9$ C for acetic acid. Therefore at -0.7 V versus RHE and 2.0 M KOH electrolyte, the FE of ethanol is 68.78% ($FE = \frac{401.1}{583.2} \times 100\% = 68.78\%$) and the FE of acetic acid is 5.13% ($FE = \frac{29.9}{583.2} \times 100\% = 5.13\%$).

Supplementary Table 6| Summary for the standard samples

Standard Samples	Sample 1	Sample 2	Sample 3	Sample 4	Sample 5
Mass (ethanol)	0.3 g	0.6 g	0.66 g	0.9 g	1.2 g
Mass (acetic acid)	0.07 g	0.14 g	0.154 g	0.21 g	0.28 g
Volume (m L)	1000	1000	1000	1000	1000
Amount of ethanol in 0.5 mL standard solution ($n_{ethanol}$)	3.256×10^{-6} mol	6.512×10^{-6} mol	7.163×10^{-6} mol	9.768×10^{-6} mol	1.302×10^{-5} mol
Amount of acetic acid in 0.5 mL standard solution ($n_{acetic\ acid}$)	5.828×10^{-7} mol	1.166×10^{-6} mol	1.282×10^{-6} mol	1.749×10^{-6} mol	2.331×10^{-6} mol
Peak area of ethanol ($A_{ethanol}$)	1.78	3.52	3.92	5.26	7.07
Peak area of acetic acid ($A_{acetic\ acid}$)	0.55	1.11	1.23	1.69	2.22
Peak area of DMSO (A_r)	1.00	1.00	1.00	1.00	1.00
Correction factor of ethanol ($f_{ethanol}$)	14.291	14.453	14.276	14.508	14.387
Correction factor of acetic acid ($f_{acetic\ acid}$)	8.278	8.207	8.143	8.085	8.203

In a full collection protocol, a flask of water for washing the CO off-gas was added to collect the liquid products before CO exhausted and the flow rate of the outlet was monitored with a flow meter (Fig. S29a). To identify the crossover of the liquid-phase products formed during the CORR, the catholyte, anolyte and water in the flask were collected and analyzed using ^{1}H-NMR. The results for CORR were exhibited in Supplementary Figs. 16-17 and 22. And the results showed that liquid products indeed migrated across GDE and AEM. By adding up all of these detected products, the total FE reached 100±3%, confirming that the liquid products in the CO off-gas and anolyte were the "missing" products. Acetone, acetaldehyde and propionaldehyde were only detected in the CO off-gas but not in the catholyte and anolyte. This should be attributed to the low production rates and high volatility.

To elucidate the carbon balance path, flow meters were used to monitor the inlet and outlet flow out of the reactor (Fig. S29a). Figs. S29b-d show the outlet flow rate as a function of current density. When J=0 mA cm^{-2}, there's no obvious discrepancy in the flow rate between gas inlet and outlet. As current densities increased, the outlet rate gradually decreased in all the electrolytes, which corresponds to a gradual enhancement in the consumption rate of CO. In addition, as OH^- cannot react with CO, the outlet flows are approximately equivalent to inlet flows at low current densities in basic solution. And the basic electrolytes cannot change the outlet flow rates, which is different from the CO_2RR in basic electrolyte (Energy Environ. Sci., 2020, 13, 977). For the final carbon balance (Supplementary Table 7), the unreacted CO flow rate after the reactor and consumed CO flow rate for the conversion into products added up to a total of ~20 mL min^{-1} at various current densities, which was equal to CO inlet flow rate used in the experiment.

Supplementary Table 7| Carbon balance in KOH solution (J is the current density; $\emptyset_{CO\ to\ gas}$ is consumption rate of CO to gas products; $\emptyset_{CO\ to\ liquid}$ is consumption rate of CO to liquid products;$\emptyset_{residual\ CO}$ is outlet flow rate of CO).

Cu/Cu_2O-A 2.0 M KOH			
J (mA cm^{-2})	$\emptyset_{CO\ to\ gas}$ (mL/min)	$\emptyset_{CO\ to\ liquid}$ (mL/min)	$\emptyset_{residual\ CO}$ (mL/min)
4	0.006	0.025	19.955

10	0.017	0.061	19.916
40	0.076	0.233	19.694
96	0.159	0.552	19.275
162	0.251	0.974	18.766
260	0.397	1.551	18.054
456	0.685	2.655	16.664
Cu/Cu_2O-A 1.0 M KOH			
J (mA cm^{-2})	$\emptyset_{CO\ to\ gas}$ (mL/min)	$\emptyset_{CO\ to\ liquid}$(mL/min)	$\emptyset_{residual\ CO}$ (mL/min)
1	0.001	0.006	19.985
2.5	0.004	0.013	19.977
10	0.020	0.046	19.926
40	0.086	0.185	19.717
96	0.185	0.497	19.323
162	0.281	0.886	18.828
260	0.453	1.502	18.016
456	0.746	2.539	16.722
Cu/Cu_2O-A 0.5 M KOH			
J (mA cm^{-2})	$\emptyset_{CO\ to\ gas}$ (mL/min)	$\emptyset_{CO\ to\ liquid}$ (mL/min)	$\emptyset_{residual\ CO}$ (mL/min)
1	0.001	0.005	19.989
2.5	0.004	0.013	19.978
10	0.017	0.049	19.915
40	0.092	0.166	19.707
96	0.217	0.413	19.364
162	0.361	0.690	18.916
299	0.706	1.320	17.966
Cu/Cu_2O-S 2.0 M KOH			
J (mA cm^{-2})	$\emptyset_{CO\ to\ gas}$ (mL/min)	$\emptyset_{CO\ to\ liquid}$ (mL/min)	$\emptyset_{residual\ CO}$(mL/min)
3.2	0.003	0.016	19.984

8.6	0.009	0.049	19.936
35.5	0.043	0.185	19.755
83	0.134	0.430	19.426
146	0.413	0.628	18.949
231	0.899	0.659	18.444
410	1.603	1.193	17.162
Cu/Cu_2O-H 2.0 M KOH			
J (mA cm^{-2})	$\emptyset_{CO\ to\ gas}$ (mL/min)	$\emptyset_{CO\ to\ liquid}$ (mL/min)	$\emptyset_{residual\ CO}$(mL/min)
2.7	0.001	0.016	19.982
6.9	0.003	0.035	19.962
27.2	0.012	0.134	19.828
64	0.030	0.370	19.612
108	0.039	0.557	19.316
177	0.093	1.035	18.833
307	0.180	1.900	17.912
Cu-butyl-A 2.0 M KOH			
J (mA cm^{-2})	$\emptyset_{CO\ to\ gas}$ (mL/min)	$\emptyset_{CO\ to\ liquid}$(mL/min)	$\emptyset_{residual\ CO}$ (mL/min)
3.6	0.003	0.025	19.974
9.2	0.009	0.063	19.915
38.4	0.04	0.245	19.722
91	0.096	0.599	19.276
156	0.171	0.985	18.836
247	0.278	1.611	18.107
433	0.562	2.681	16.762
Cu-butyl-S 2.0 M KOH			
J (mA cm^{-2})	$\emptyset_{CO\ to\ gas}$ (mL/min)	$\emptyset_{CO\ to\ liquid}$ (mL/min)	$\emptyset_{residual\ CO}$ (mL/min)
3	0.007	0.017	19.981
7.9	0.018	0.041	19.935
34	0.084	0.164	19.746

79	0.220	0.430	19.354
140	0.477	0.646	18.883
220	0.938	0.780	18.276
390	1.728	1.210	17.055
Cu-butyl-H 2.0 M KOH			
J (mA cm^{-2})	$\emptyset_{CO\ to\ gas}$ (mL/min)	$\emptyset_{CO\ to\ liquid}$ (mL/min)	$\emptyset_{residual\ CO}$ (mL/min)
2.5	0.002	0.015	19.984
6.6	0.006	0.035	19.955
28.2	0.032	0.139	19.832
65.5	0.065	0.355	19.582
116	0.154	0.576	19.255
182	0.252	0.949	18.821
323	0.508	1.631	17.855

References

[1] Jouny, M., Luc, W. & Jiao, F. High-rate electroreduction of carbon monoxide to multi-carbon products. *Nat. Catal.* **1**, 748-755 (2018).

[2] Christina W. Li, Jim Ciston & Matthew W. Kanan. Electroreduction of carbon monoxide to liquid fuel on oxide-derived nanocrystalline copper. *Nature* **508**, 504-507 (2014).

[3] Chen, R. X. *et al*. Highly selective production of Ethylene by the electroreduction of carbon monoxide. *Angew. Chem. Int. Ed.* **59**, 154-160 (2020).

[4] Feng, X. F., Jiang, K. L., Fan, S. S. & Kanan, M. W. A direct grain-boundary-Activity correlation for CO electroreduction on Cu nanoparticles. *ACS Central. Sci.* **2**, 169-174 (2016).

[5] Bertheussen, E., Hogg, T. V., Abghoui, Y., Engstfeld, A. K., Chorkendorff, I. & Stephens, I. E. L. Electroreduction of CO on polycrystalline copper at low overpotentials. *ACS Energy Lett.* **3**, 634-640 (2018).

[6] Raciti, D. *et al* . Low-Overpotential electroreduction of carbon monoxide using copper nanowires. *ACS Catal.* **7**, 4467-4472 (2017).

[7] Luc, W. *et al.* Two-dimensional copper nanosheets for electrochemical reduction of carbon monoxide to acetate. *Nat. Catal.* **2**, 423-430 (2019).

[8] Wang, L. *et al.* Electrochemically converting carbon monoxide to liquid fuels by directing selectivity with electrode surface area. *Nat. Catal.* **2**, 702-708 (2019).

[9] Zhuang, T. T. *et al.* Copper nanocavities confine intermediates for efficient electrosynthesis of C_3 alcohol fuels from carbon monoxide. *Nat. Catal.* **1**, 946-951 (2018).

[10] Pang, Y. J. *et al.* Efficient electrocatalytic conversion of carbon monoxide to propanol using fragmented copper. *Nat. Catal.* **2**, 251-258 (2019).

[11] Li, J. *et al.* Constraining CO coverage on copper promotes high-efficiency ethylene electroproduction. *Nat. Catal.* **2**, 1124-1131(2019).

[12] Wang, X. *et al.* Efficient upgrading of CO to C_3 fuel using asymmetric C-C coupling active sites. *Nat. Commun.* **10**, 5186 (2019).

[13] Li, J. *et al.*. Copper adparticle enabled selective electrosynthesis of n-propanol. *Nat. Commun.* **9**, 4614 (2018).

[14] Verdaguer-Casadevall, A. *et al.* Probing the active surface sites for CO reduction on oxide-derived copper electrocatalysts. *J. Am. Chem. Soc.* **137**, 9808-9811 (2015).

[15] Li, J. *et al.* Enhanced multi-carbon alcohol electroproduction from CO via modulated hydrogen adsorption. *Nat. Commun.* **11**, 3685 (2020).

[16] Hoang, T. T. H., Ma, S. C., Gold, J. I., Kenis, P. J. A. & Gewirth, A. A. Nanoporous Copper Films by additive-controlled electrodeposition: CO_2 reduction catalysis. *ACS Catal.* **7**, 3313-3321 (2017).

[17] Hoang, T. T. H. *et al*. Nanoporous Copper-Silver alloys by additive-controlled electrodeposition for the selective electroreduction of CO_2 to ethylene and ethanol. *J. Am. Chem. Soc*. **140**, 5791-5797 (2018).

[18] Dinh, C.-T. *et al*. CO_2 electroreduction to ethylene via hydroxide-mediated copper catalysis at an abrupt interface. *Science* **360**, 783-787 (2018).

[19] Loiudice, A. *et al*. Tailoring copper nanocrystals towards C_2 products in electrochemical CO_2 reduction. *Angew. Chem .Int. Ed*. **55**, 5789-5792 (2016).

[20] Gao, D. F. *et al*. Palsma-Ativated copper nanocube catalysts for efficient carbon dioxide electroreduction to hydrocarbons and alcohols. *ACS Nano* **11**, 4825-4831(2017).

[21] Mistry, H. *et al*. Highly selective plasma-activated copper catalysts for carbon dioxide reduction to ethylene. *Nat.Commun*. **7**, 12123 (2016).

[22] Song, Y. *et al*. Highly-Selectivity electrochemical conversion of CO_2 to ethanol using a copper nanoparticles/N-doped graphene electrode. *ChemistrySelect*. **1**, 1-8 (2016).

[23] De Luna, P. *et al*. Catalyst electro-redeposition controls morphology and oxidation state for selevtive carbon dioxide reduction. *Nat. Catal*. **1**, 103-110 (2018).

[24] Handoko, A.D. *et al*. Mechanistic insights into the selective electroreduction of carbon dioxide to ethylene on Cu_2O-derived copper catalysts. *J. phys. Chem. C*. **120**, 20058-20067 (2016).

[25] Jiang, K. *et al*. Metal ion cycling of Cu foil for selective C-C coupling in electrochemical CO_2 reduction. *Nat. Catal*. **1**, 111-119 (2018).

[26] Hori, Y., Takahashi, I., Koga,O & Hoshi, N. Elevtrochemical reduction of carbon dioxide at various series of copper single crystal electrodes. *J. Mol. Catal. A. Chem*. **199**, 39-47 (2003).

[27] Ren, D., Deng, Y. L., Handoko, A. D., Chen, C. S., Malkhandi, S. & Yeo, B. S. Selective electrochemical reduction of carbon dioxide to ethylene and ethanol on copper (I) oxide catalysts. *ACS Catal*. **5**, 2814-2821 (2015).

[28] Dutta, A., Rahaman, M., Luedi, N.C., Mohos, M & Broekmann, P. Morphology Matters: tuning the product distribution of CO_2 electroreduction on oxide-derived Cu foam catalysts. *ACS Catal.* **6**, 3804-3814 (2016).

[29] Wakerley, D. *et al.* Bio-inspired hydrophobicity promotes CO_2 reduction on a Cu surface. *Nat. Mater.* **18**, 1222-1227 (2019).

[30] Chen, C. S., Wan, J. H. & Yeo, B. S. Electrochemical reduction of carbon dioxide to ethane using nanostructured Cu_2O-derived copper catalyst and palladium (II) chloride. *J. Phys. Chem. C.* 119, 26875-26882 (2015).

[31] Zhu, Q. G. *et al.* Carbon dioxide electroreduction to C_2 products over copper-cuprous oxide derived from electrosynthesized copper complex. *Nat. Commun.* **10**, 3851 (2019).

[32] Gao, Y. G. *et al.* Cu_2O nanoparticles with both {100} and {111} facets for enhancing the selectivity and activity of CO_2 electroreduction to ethylene. *Adv. Sci.* **7**, 1902820 (2020).

[33] Yang, P.-P. *et al.* Protecting copper oxidation state via intermediate confinement for selective CO_2 electroreduction to C_{2+} fuels. *J. Am. Chem. Soc.* **142**, 6400-6408 (2020).

[34] Wang, H. X. *et al.* Synergistic enhancement of electrocatalytic CO_2 reduction to C_2 oxygenates at nitrogen-doped nanodiamonds/Cu interface. *Nat. Nanotechnol* **15**, 131-137 (2020).

[35] Chen, X. Y. *et al.* Controlling speciation during CO_2 reduction on Cu-alloy electrodes. *ACS Catal.* **10**, 672-682 (2020).

[36] Luo, M. C. *et al.* Hydroxide promotes carbon dioxide electroreduction to ethanol on copper via tuning of adsorbed hydrogen. *Nat. Commun.* **10**, 5814 (2019).

[37] Li, F. W. *et al.* Cooperative CO_2-to-ethanol conversion via enriched intermediates at molecule-metal catalyst interfaces. *Nat. Catal.* **3**, 75-82 (2020).

[38] Wang, Y. H. *et al.* Catalyst synthesis under CO_2 electroreduction favours faceting and promotes renewable fuels electrosynthesis. *Nat. Catal.* **3**, 98-106 (2020).

[39] Wang, Y. X. *et al.* Copper nanocubes for CO_2 reduction in gas diffusion electrodes. *Nano Lett.* **19**, 8461-9468 (2019).

[40] Yin, Z. Y. *et al.* Cu_3N nanocubes for selective electrochemical reduction of CO_2 to ethylene. *Nano Lett.* **19**, 8658-8663 (2019).

[41] Zou, C. Q. *et al.* Porous copper microspheres for selective production of multicarbon fuels via CO_2 electroreduction. *Small* **15**, 1902582 (2019).

[42] Ma, S. C., Sadakiyo, M., Luo, R., Heima, M., Yamauchi, M. & kenis, P. J. A. One-step electrosynthesis of ethylene and ethanol from CO_2 in an alkaline electrolyzer. *J. Power Sources* **301**, 219-228 (2016).

[43] Lv, J.-J., Jouny, M., Luc, W., Zhu, W. L., Zhu, J.-J. & Jiao, F. A highly porous copper electrocatalyst for carbon dioxide reduction. *Adv. Mater.* **30**, e1803111 (2018).

[44] Mi, Y. Y., Shen, S. B., Peng, X. Y., Bao, H. H., Liu, X. J. & Luo, J. Selective electroreduction of CO_2 to C2 products over Cu_3N-derived Cu nanowires. *ChemElectroChem* **6**, 2393-2397 (2019).

[45] Kim, D., Kley, C. S., Li, Y. F. & Yang, P. D. Copper nanoparticle ensembles for selective electroreduction of CO_2 to C_2-C_3 products. *Proc. Natl Acad. Sci. USA* **114**, 10560-10565 (2017).

[46] Arán-Ais, R. M., Scholten, F., Kunze, S., Rizo, R. & Cuenya, B. R. The role of in site generated morphological motifs and Cu(I) species in C_{2+} product selectivity during CO_2 pulsed electroreduction. *Nat. Energy* **5**, 317-325 (2020).

[47] Wang, X. *et al.* Efficient electrically powered CO_2-to-ethanol via suppression of deoxygenation. *Nat. Energy* **5**, 478-486 (2020).

[48] Ma, W. C. *et al.* Electrocatalytic reduction of CO_2 to ethylene and ethanol through hydrogen-assisted C-C coupling over fluorine-modified copper. *Nat. Catal.* **3**, 478-487 (2020).

[49] Xu, A. N. *et al.* Copper/alkaline earth metal oxide interfaces for electrochemical CO_2 to alcohol conversion by selective hydrogenation. *Nat. Catal.* **5**, 1081-1088 (2022).

[50] kim, C. *et al*. Tailored catalyst microenvironments for CO_2 electroreduction to multicarbon products on copper using bilayer ionomer coatings. *Nat. Energy* **6**, 1026-1034 (2021).

[51] Su, X. Z. *et al*. Complementary operando spectroscopy identification of in-situ generated metastable charge asymmetry Cu_2-CuN_3 clusters for CO_2 reduction to ethanol. *Nat. Commun.* **13**, 1322 (2022).

[52] Wang, P. T. *et al*. Boosting electrocatalytic CO_2 to ethanol production via asymmetric C-C coupling. *Nat. Commun.* **13**, 3754 (2022).

[53] Yang, B. Y. *et al*. Electrocatalytic CO_2 reduction to alcohols by modulating the molecular geometry and Cu coordination in bicentric copper complexes. *Nat. Commun.* **13**, 5122 (2022).

[54] Klamt, A., Jonas, V., Burge, T. & Lohrenz, J.C.W. Refinement and parametrization of COSMO-RS. *J. Phys. Chem. A*. **102**, 5074–5085 (1998).

[55] Klamt, A. & Eckert, F. COSMO-RS: A novel and efficient method for the a priori prediction of thermophysical data of liquids. *Fluid Phase Equilib*. **172**, 43-72 (2000).

[56] Abraham, M. J., Murtola, T., Schulz, R., Pall, S., Smith, J. C., Hess, B. & Lindahl, E. GROMACS: High performance molecular simulations through multi-level parallelism from laptops to supercomputers. *SoftwareX* **1**, 19-25 (2015).

[57] Martinez, L., Andrade, R. E., Birgin, G. & Martinez, J. M. PACKMOL: a package for building initial configurations for molecular dynamics simulations. *J. Comput. Chem*. **30**, 2157-2164 (2009).

[58] Poudyal, I., & Adhikari, N. P. Temperature dependence of diffusion coefficient of car bon monoxide in water: A molecular dynamics study. *J. Mol. Liq*. **194**, 77–84 (2014).

[59] Wang, J., Wolf, R. M., Caldwell, J. W., Kollman, P. A. & Case, D. A. Development and testing of a general amber force field. *J. Comput. Chem*. **25**, 1157-1174 (2004).

[60] Bussi, G., Donadio, D. & Parrinello, M. Canonical sampling through velocity rescaling. *J. Chem. Phys*. **126**, 014101 (2007).

[61] Berendsen, H. J., Postma, J. V., van Gunsteren, W. F., DiNola, A. & Haak, J. Molecular dynamics with coupling to an external bath. *J. Chem. Phys*. **81**, 3684-3690 (1984).

[62] Parrinell, M. & Rahman, A. Polymorphic transitions in single crystals: A new molecular dynamics method *J. Appl. Phys*. **52**, 7182-7190 (1981).

[63] Darden, T., York, D. & Pedersen, L. Particle mesh Ewald: an N*log (N) method for Ewald sums in large systems. *J. Chem. Phys*. **98**, 10089-10092 (1993).

[64] Hess, B., Bekker, H., Berendsen, H. J. & Fraaije, J. G. LINCS: A linear constraint solver for molecular simulations. *J. Comput. Chem*. **18**, 1463-1472 (1997).
