# Supplementary material for: A hydrophobic Cu/Cu2O sheet catalyst for selective electroreduction of CO to ethanol
Source: Nat Commun. 2023 Jan 31;14:501. doi: 10.1038/s41467-023-36261-1 (PMC9889799; doi:10.1038/s41467-023-36261-1)
Supplement: Supplementary file 2 — Source Data [file 41467_2023_36261_MOESM2_ESM.zip › Source data for Figure 4b and Supplementary Figure 11/GC data of calibrating gas/BF1-1212-1924-50ppm-1mL.pdf]

批次:　　　　1mL
实验单位:
计算方法:　　外标法
采样开始:　　2022-12-12 19:24:59
分析周期:　　19.00　min　　　　　　斜率/峰宽:　100.0/1.0
谱图文件名:　BF1-1212-1924-50ppm-1mL.src

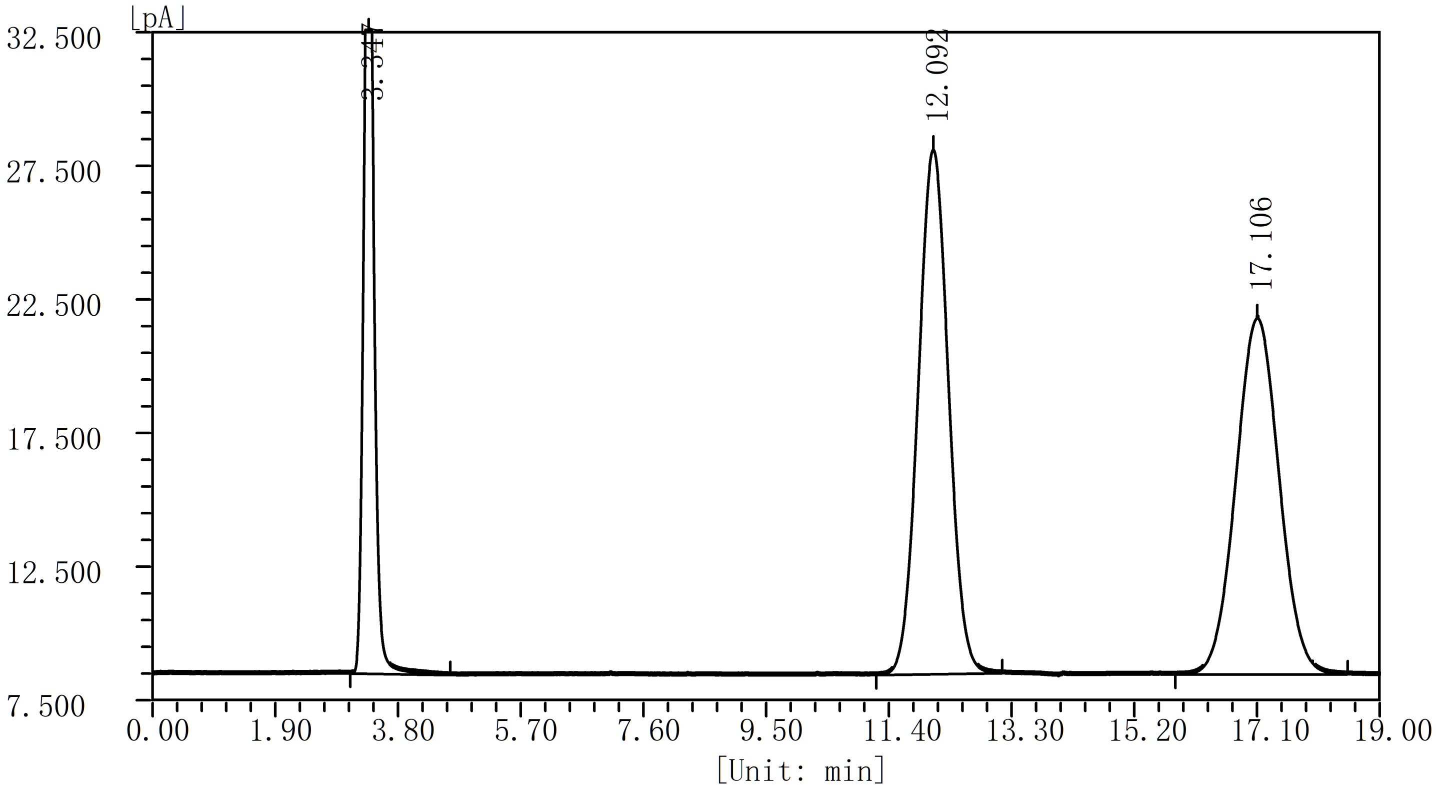

分析结果

峰序	组分名	保留时间 [min]	半峰宽 [min]	峰高 [uV]	峰面积 [uV*s]	峰面积 [%]	含量 [%]	峰类型
1	CH4	3.347	0.161	32255.1	345536.0	0.0000	49.9000	BB
2	C2H4	12.092	0.507	19637.4	640092.5	0.0000	51.2000	BB
3	C2H6	17.106	0.724	13338.2	620616.2	0.0000	50.5000	BB
			总计:	65230.7	[illegible]606244.[illegible]	0.0000	151.6000	
