# Supplementary material for: A hydrophobic Cu/Cu2O sheet catalyst for selective electroreduction of CO to ethanol
Source: Nat Commun. 2023 Jan 31;14:501. doi: 10.1038/s41467-023-36261-1 (PMC9889799; doi:10.1038/s41467-023-36261-1)
Supplement: Supplementary file 2 — Source Data [file 41467_2023_36261_MOESM2_ESM.zip › Source data for Figure 4b and Supplementary Figure 11/GC data of calibrating gas/BF1-1213-0933-1000ppm-1mL.pdf]

批次:　　　　1mL
实验单位:
计算方法:　　外标法
采样开始:　　2022-12-13 09:33:10
分析周期:　　19.00　min　　　　　斜率/峰宽:　100.0/1.0
谱图文件名:　BF1-1213-0933-1000ppm-1mL.src

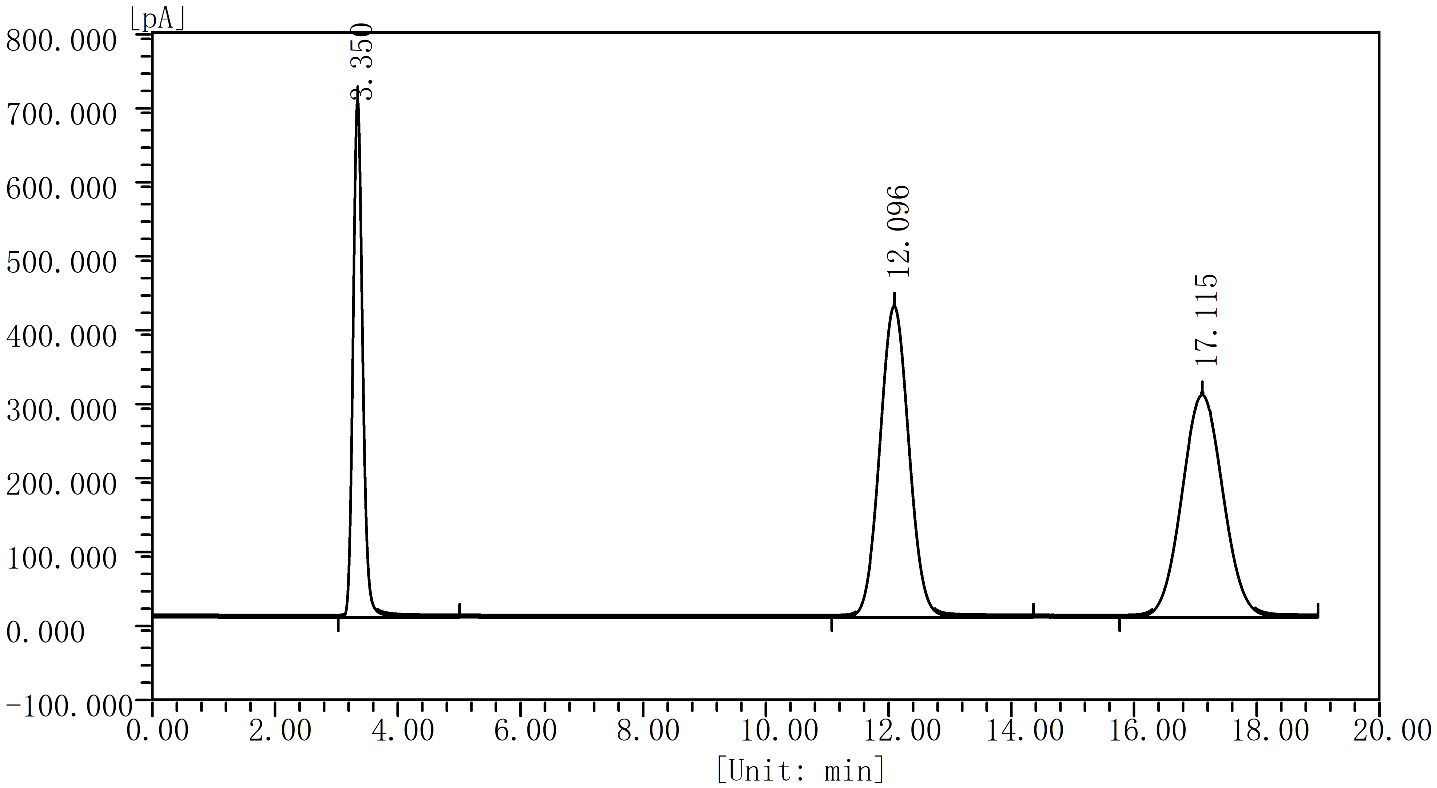

分析结果

峰序	组分名	保留时间 [min]	半峰宽 [min]	峰高 [uV]	峰面积 [uV*s]	峰面积 [%]	含量 [%]	峰类型
1	CH4	3.350	0.164	699659.7	7574599.5	0.0000	1003.0000	BB
2	C2H4	12.096	0.513	420975.6	3889456.	0.0000	991.0000	BB
3	C2H6	17.115	0.731	300909.5	4101661.	0.0000	1021.0000	BB
			总计:	1421544.8	5565716.	0.0000	3015.0000	
