# Supplementary material for: A hydrophobic Cu/Cu2O sheet catalyst for selective electroreduction of CO to ethanol
Source: Nat Commun. 2023 Jan 31;14:501. doi: 10.1038/s41467-023-36261-1 (PMC9889799; doi:10.1038/s41467-023-36261-1)
Supplement: Supplementary file 2 — Source Data [file 41467_2023_36261_MOESM2_ESM.zip › Source data for Figure 4b and Supplementary Figure 11/GC data of calibrating gas/BF1-1213-1253-700ppm.pdf]

批次:　　　　0.7

实验单位:

计算方法:　　外标法

采样开始:　　2022-12-13 12:53:03

分析周期:　　19.00 min　　　　斜率/峰宽: 100.0/1.0

谱图文件名:　BF1-1213-1253-1000ppm-0.7.src

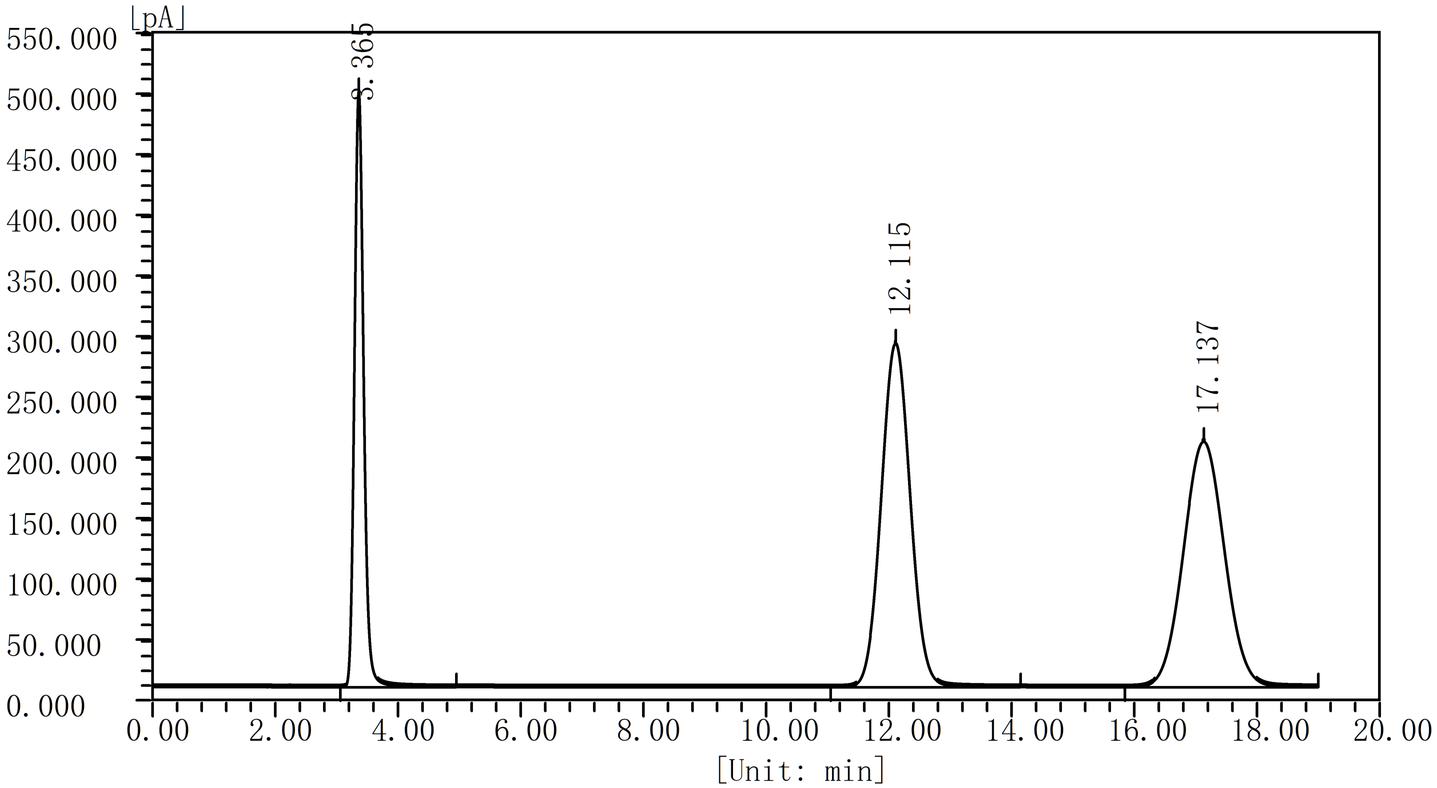

分析结果

峰序	组分名	保留时间 [min]	半峰宽 [min]	峰高 [uV]	峰面积 [uV*s]	峰面积 [%]	含量 [%]	峰类型
1	CH4	3.365	0.157	490917.2	5120240.9	0.0000	702.1000	BB
2	C2H4	12.115	0.511	283706.1	[illegible]326795.7	0.0000	693.7000	BB
3	C2H6	17.137	0.728	202583.0	[illegible]461652.8	0.0000	714.7000	BB
			总计:	977206.3	3908688.	0.0000	2110.5000	
