# Supplementary material for: A hydrophobic Cu/Cu2O sheet catalyst for selective electroreduction of CO to ethanol
Source: Nat Commun. 2023 Jan 31;14:501. doi: 10.1038/s41467-023-36261-1 (PMC9889799; doi:10.1038/s41467-023-36261-1)
Supplement: Supplementary file 2 — Source Data [file 41467_2023_36261_MOESM2_ESM.zip › Source data for Figure 4b and Supplementary Figure 11/GC data of calibrating gas/BF1-1213-1707-500ppm.pdf]

批次:　　　　0.5

实验单位:

计算方法:　　外标法

采样开始:　　2022-12-13 17:07:04

分析周期:　　19.00 min　　　　斜率/峰宽:　100.0/1.0

谱图文件名:　BF1-1213-1707-1000ppm-0.5.src

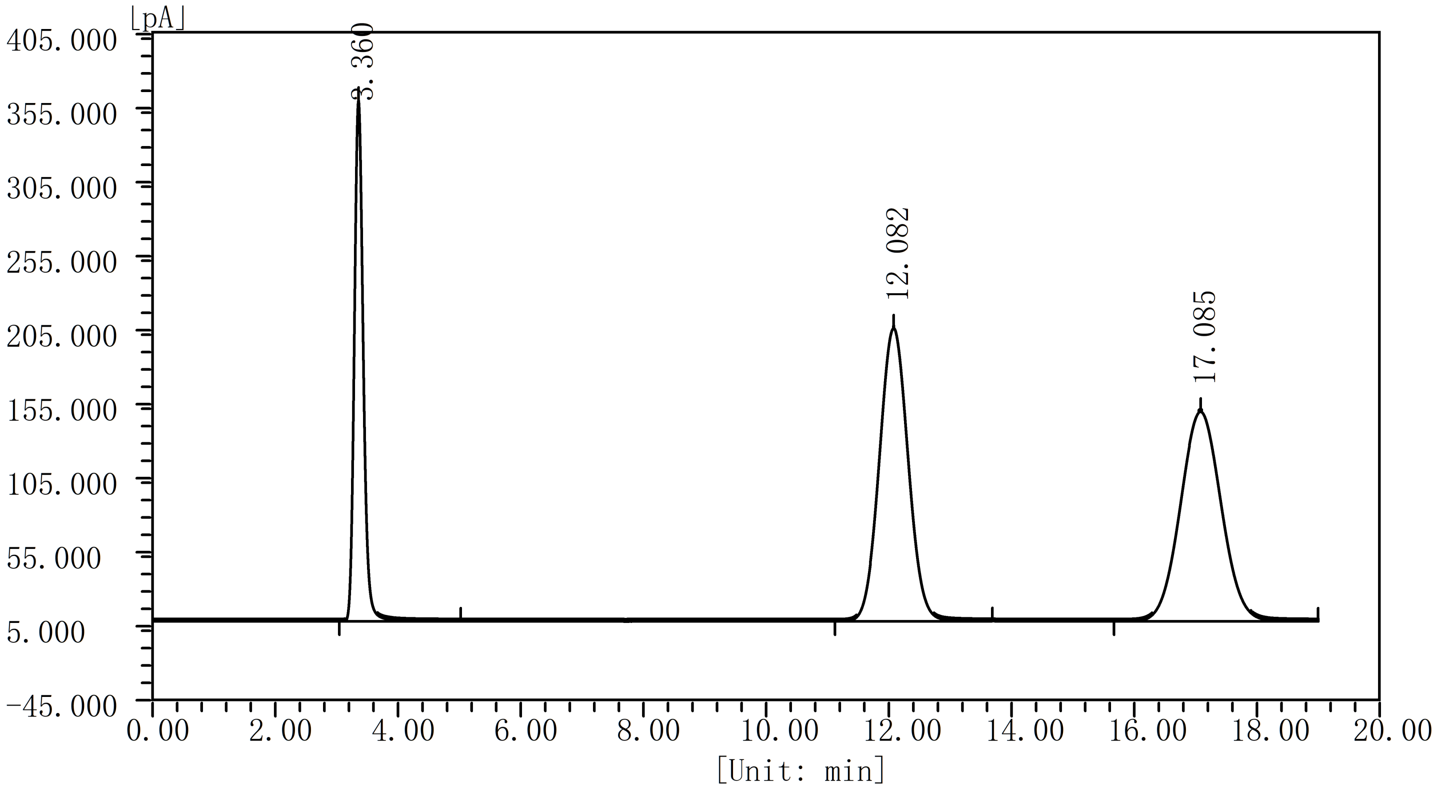

分析结果

峰序	组分名	保留时间 [min]	半峰宽 [min]	峰高 [uV]	峰面积 [uV*s]	峰面积 [%]	含量 [%]	峰类型
1	CH4	3.360	0.153	351742.8	[illegible]575685.4	0.0000	501.5000	BB
2	C2H4	12.082	0.508	198116.6	[illegible]473348.2	0.0000	495.5000	BB
3	C2H6	17.085	0.724	141702.3	[illegible]584793.5	0.0000	510.5000	BB
			总计:	691561.7	6633826.	0.0000	[illegible]507.500[illegible]	
