# Supplementary material for: A hydrophobic Cu/Cu2O sheet catalyst for selective electroreduction of CO to ethanol
Source: Nat Commun. 2023 Jan 31;14:501. doi: 10.1038/s41467-023-36261-1 (PMC9889799; doi:10.1038/s41467-023-36261-1)
Supplement: Supplementary file 2 — Source Data [file 41467_2023_36261_MOESM2_ESM.zip › Source data for Figure 4b and Supplementary Figure 11/GC data of calibrating gas/BF1-1213-1817-300ppm.pdf]

批次:　　　　0.3
实验单位:
计算方法:　　外标法
采样开始:　　2022-12-13 18:17:42
分析周期:　　19.00　min　　　　　斜率/峰宽:　100.0/1.0
谱图文件名:　BF1-1213-1817-1000ppm-0.3.src

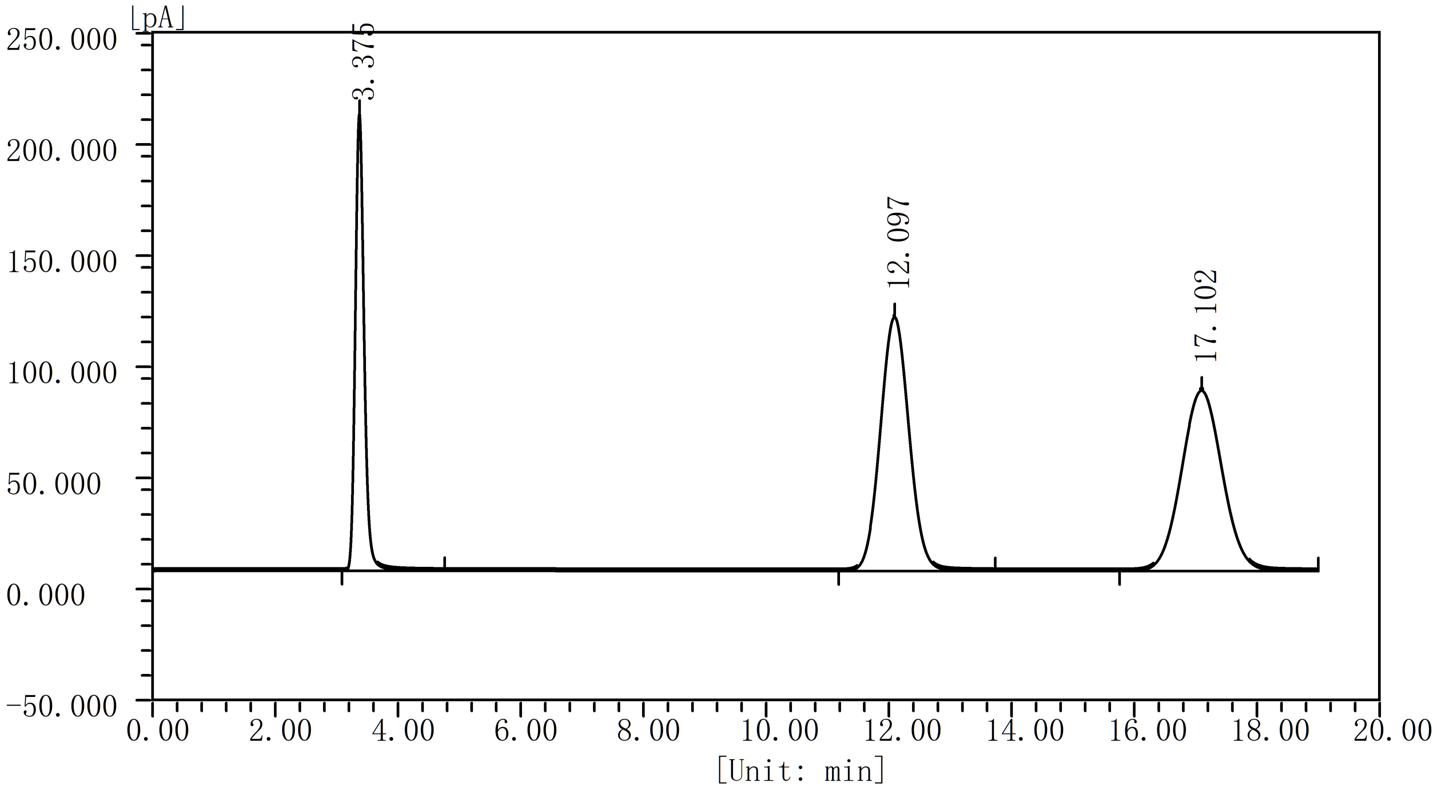

分析结果

峰序	组分名	保留时间 [min]	半峰宽 [min]	峰高 [uV]	峰面积 [uV*s]	峰面积 [%]	含量 [%]	峰类型
1	CH4	3.375	0.150	205660.2	[illegible]062487.[illegible]	0.0000	300.9000	BB
2	C2H4	12.097	0.506	114303.2	[illegible]728638.[illegible]	0.0000	297.3000	BB
3	C2H6	17.102	0.723	81265.4	[illegible]776854.[illegible]	0.0000	306.3000	BB
			总计:	401228.9	[illegible]567980.[illegible]	0.0000	904.5000	
