# Supplementary material for: A hydrophobic Cu/Cu2O sheet catalyst for selective electroreduction of CO to ethanol
Source: Nat Commun. 2023 Jan 31;14:501. doi: 10.1038/s41467-023-36261-1 (PMC9889799; doi:10.1038/s41467-023-36261-1)
Supplement: Supplementary file 2 — Source Data [file 41467_2023_36261_MOESM2_ESM.zip › Source data for Figure 4b and Supplementary Figure 11/Gas Products (Supplementary Figure 11a)/BF1-1-2.pdf]

批次:　　　2
实验单位:
计算方法:　外标法
采样开始:　2022-11-14 17:19:31
分析周期:　18.00 min　　　斜率/峰宽: 100.0/1.0
谱图文件名: BF1-1-2.src

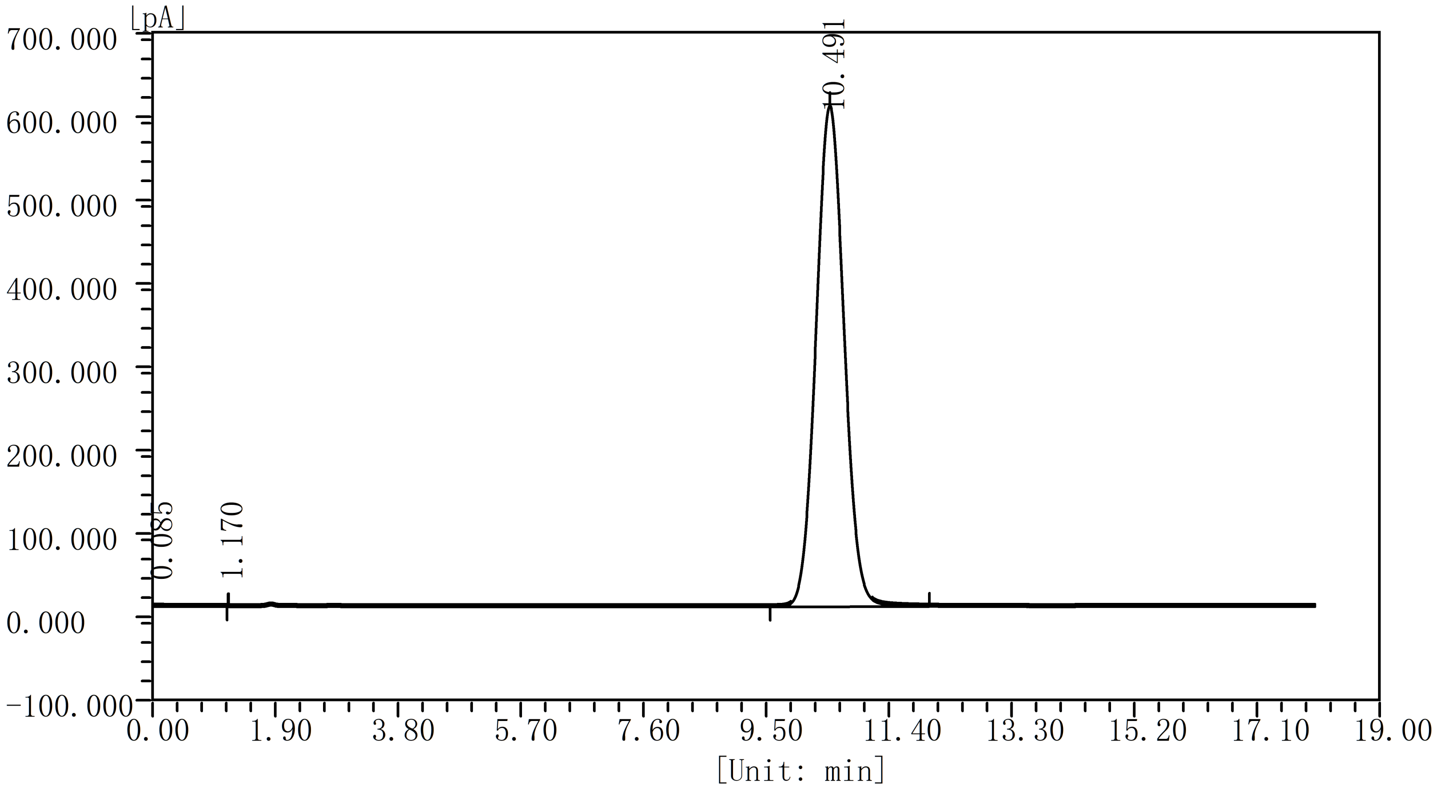

分析结果

峰序	组分名	保留时间 [min]	半峰宽 [min]	峰高 [uV]	峰面积 [uV*s]	峰面积 [%]	含量 [%]	峰类型
1		0.085	0.134	72.1	835.0	0.0000	0.0000	BB
2		1.170	0.017	1.9	1.6	0.0000	0.0000	BB
3		10.491	0.469	600941.2	8143225.	0.0000	0.0000	BB
			总计:	601015.2	8144062.	0.0000	0.0000	
