# Supplementary material for: A hydrophobic Cu/Cu2O sheet catalyst for selective electroreduction of CO to ethanol
Source: Nat Commun. 2023 Jan 31;14:501. doi: 10.1038/s41467-023-36261-1 (PMC9889799; doi:10.1038/s41467-023-36261-1)
Supplement: Supplementary file 2 — Source Data [file 41467_2023_36261_MOESM2_ESM.zip › Source data for Figure 4b and Supplementary Figure 11/Gas Products (Supplementry Figure 11b)/BF1-2-13.pdf]

批次:　　　　13
实验单位:
计算方法:　　外标法
采样开始:　　2022-11-18 11:12:57
分析周期:　　18.00　min　　　　　　斜率/峰宽:　100.0/1.0
谱图文件名:　BF1-2-13.src

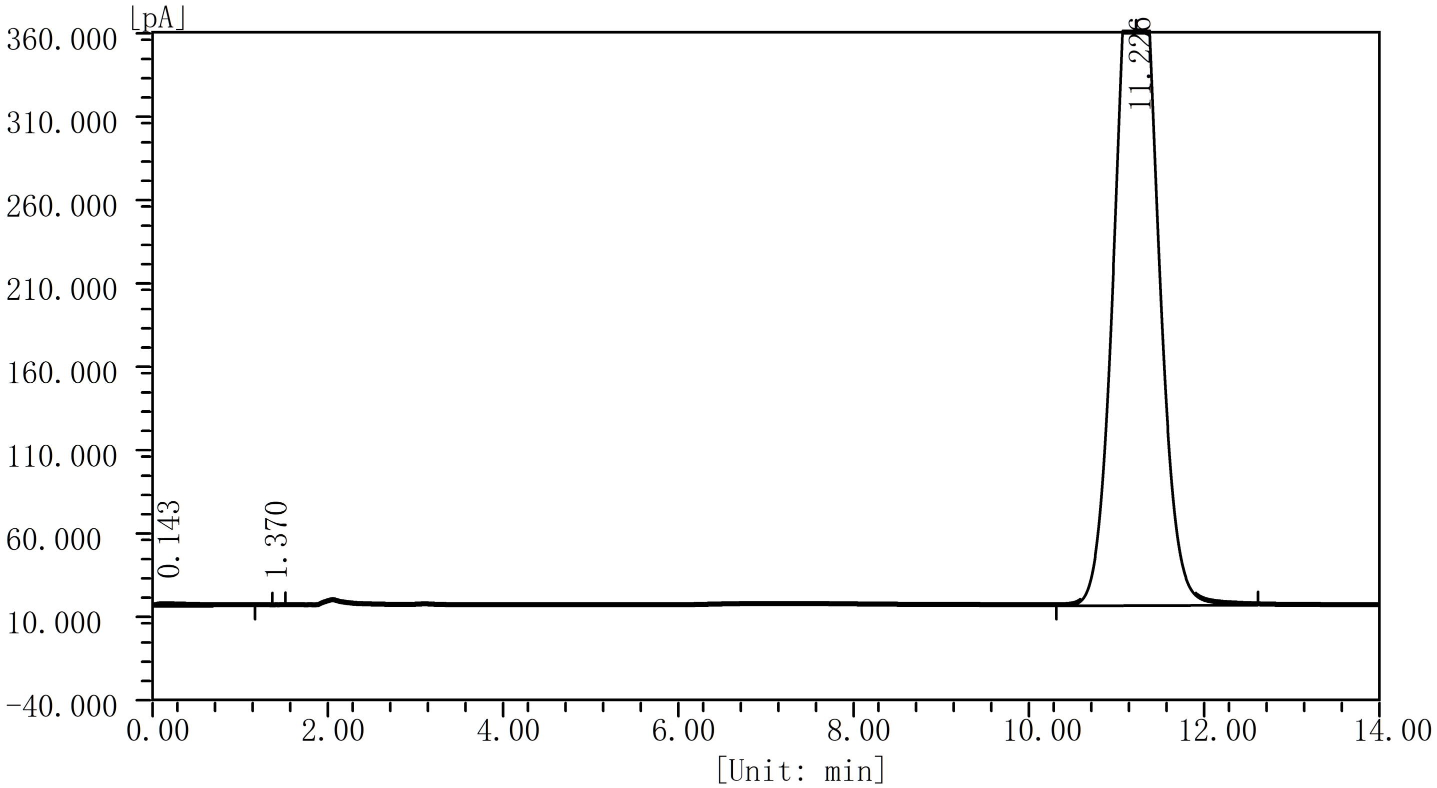


# 分析结果

| 峰序 | 组分名 | 保留时间 [min] | 半峰宽 [min] | 峰高 [uV] | 峰面积 [uV*s] | 峰面积 [%] | 含量 [%] | 峰类型 |
|---|---|---|---|---|---|---|---|---|
| 1 | | 0.143 | 0.456 | 801.9 | 20840.3 | 0.0000 | 0.0000 | BV |
| 2 | | 1.370 | 0.211 | 20.4 | 215.5 | 0.0000 | 0.0000 | BB |
| 3 | | 11.226 | 0.485 | 457433.0 | 4266785. | 0.0000 | 0.0000 | BB |
| | | | 总计: | 458255.3 | 4287841. | 0.0000 | 0.0000 | |
