# Supplementary material for: A hydrophobic Cu/Cu2O sheet catalyst for selective electroreduction of CO to ethanol
Source: Nat Commun. 2023 Jan 31;14:501. doi: 10.1038/s41467-023-36261-1 (PMC9889799; doi:10.1038/s41467-023-36261-1)
Supplement: Supplementary file 2 — Source Data [file 41467_2023_36261_MOESM2_ESM.zip › Source data for Figure 4b and Supplementary Figure 11/Gas Products (Supplementry Figure 11b)/BF1-2-22.pdf]

批次:　　　　22
实验单位:
计算方法:　　外标法
采样开始:　　2022-11-18 15:31:10
分析周期:　　18.00　min　　　　　斜率/峰宽:　100.0/1.0
谱图文件名:　BF1-2-22.src

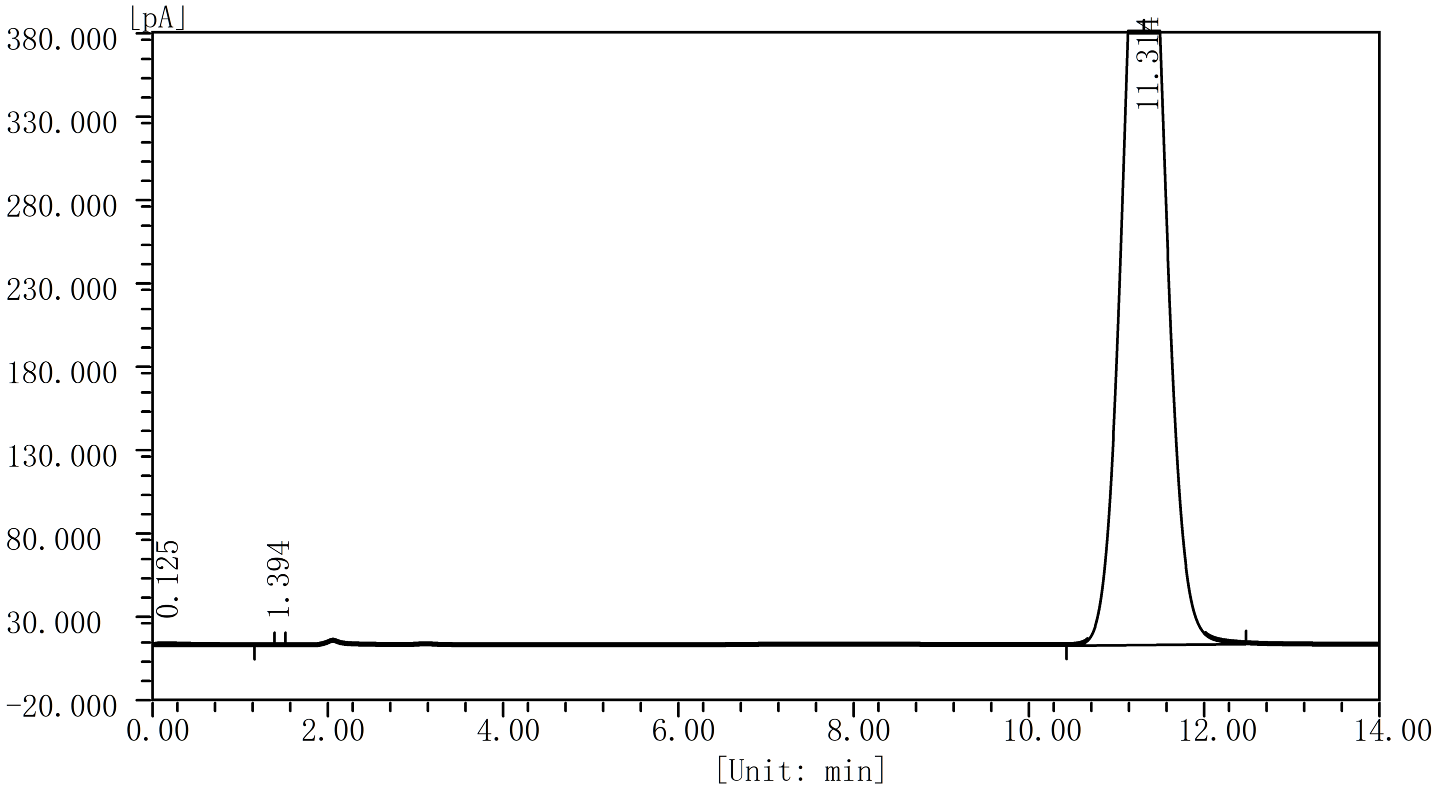

分析结果

峰序	组分名	保留时间 [min]	半峰宽 [min]	峰高 [uV]	峰面积 [uV*s]	峰面积 [%]	含量 [%]	峰类型
1		0.125	0.383	565.7	15167.6	0.0000	0.0000	BV
2		1.394	0.232	18.5	96.1	0.0000	0.0000	BB
3		11.314	0.489	542528.0	7048574.	0.0000	0.0000	BB
			总计:	543112.2	7063838.	0.0000	0.0000	
