# Supplementary material for: A hydrophobic Cu/Cu2O sheet catalyst for selective electroreduction of CO to ethanol
Source: Nat Commun. 2023 Jan 31;14:501. doi: 10.1038/s41467-023-36261-1 (PMC9889799; doi:10.1038/s41467-023-36261-1)
Supplement: Supplementary file 2 — Source Data [file 41467_2023_36261_MOESM2_ESM.zip › Source data for Figure 4b and Supplementary Figure 11/Gas Products (Supplementry Figure 11b)/BF1-2-24.pdf]

批次:　　　　24
实验单位:
计算方法:　　外标法
采样开始:　　2022-11-15 09:35:53
分析周期:　　18.00　min　　　　　斜率/峰宽:　100.0/1.0
谱图文件名:　BF1-2-24.src

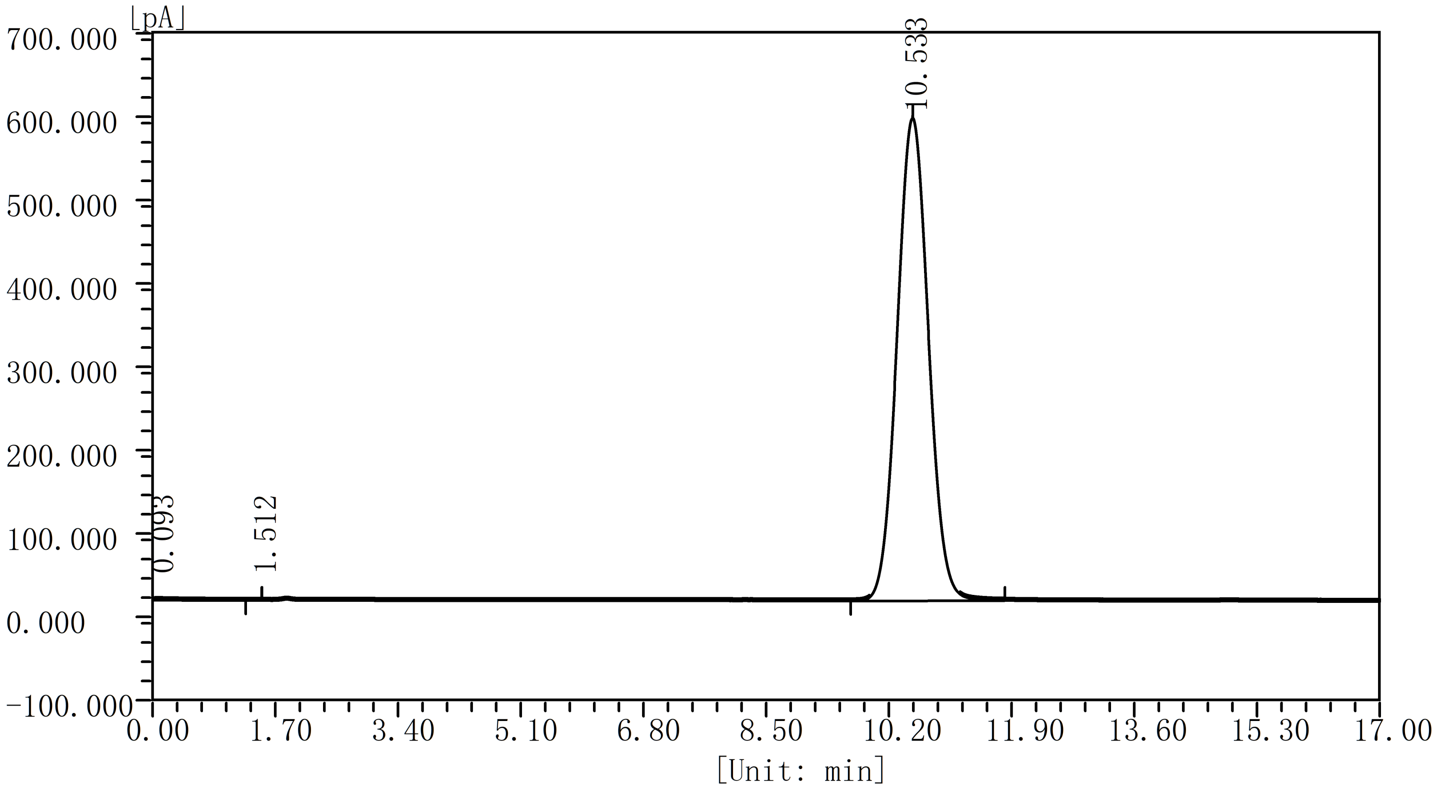

分析结果

峰序	组分名	保留时间 [min]	半峰宽 [min]	峰高 [uV]	峰面积 [uV*s]	峰面积 [%]	含量 [%]	峰类型
1		0.093	0.202	330.6	5850.9	0.0000	0.0000	BB
2		1.512	0.013	2.4	36.8	0.0000	0.0000	BB
3		10.533	0.469	579213.	57460498.	0.0000	0.0000	BB
			总计:	579546.	67466384.	0.0000	0.0000	
