# Supplementary material for: A hydrophobic Cu/Cu2O sheet catalyst for selective electroreduction of CO to ethanol
Source: Nat Commun. 2023 Jan 31;14:501. doi: 10.1038/s41467-023-36261-1 (PMC9889799; doi:10.1038/s41467-023-36261-1)
Supplement: Supplementary file 2 — Source Data [file 41467_2023_36261_MOESM2_ESM.zip › Source data for Figure 4b and Supplementary Figure 11/Gas Products (Supplementry Figure 11b)/BF1-2-25.pdf]

批次:　　　　25
实验单位:
计算方法:　　外标法
采样开始:　　2022-11-19 08:49:14
分析周期:　　18.00　min　　　　　　斜率/峰宽:　100.0/1.0
谱图文件名:　BF1-2-25.src

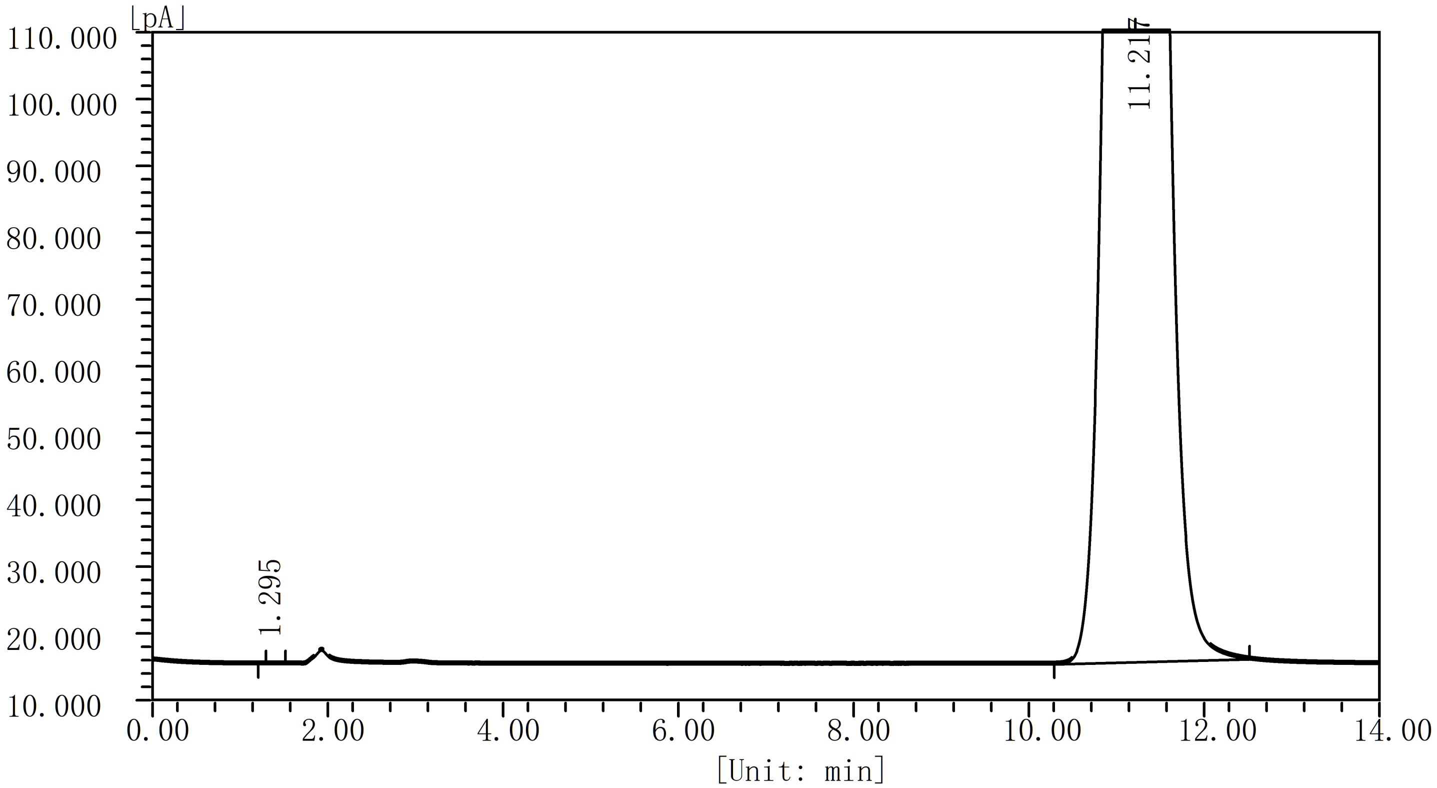

分析结果

峰序	组分名	保留时间 [min]	半峰宽 [min]	峰高 [uV]	峰面积 [uV*s]	峰面积 [%]	含量 [%]	峰类型
1		1.295	0.118	6.6	30.3	0.0000	0.0000	BB
2		11.217	0.492	510419.	46146046.	0.0000	0.0000	BB
			总计:	510426.	06146077.	0.0000	0.0000	
