# Supplementary material for: A hydrophobic Cu/Cu2O sheet catalyst for selective electroreduction of CO to ethanol
Source: Nat Commun. 2023 Jan 31;14:501. doi: 10.1038/s41467-023-36261-1 (PMC9889799; doi:10.1038/s41467-023-36261-1)
Supplement: Supplementary file 2 — Source Data [file 41467_2023_36261_MOESM2_ESM.zip › Source data for Figure 4b and Supplementary Figure 11/Gas Products (Supplementry Figure 11b)/BT2-2-12.pdf]

批次: 12
实验单位:
计算方法: 外标法
采样开始: 2022-11-16 22:08:43
分析周期: 18.00 min 斜率/峰宽: 100.0/1.0
谱图文件名: BT2-2-12.src

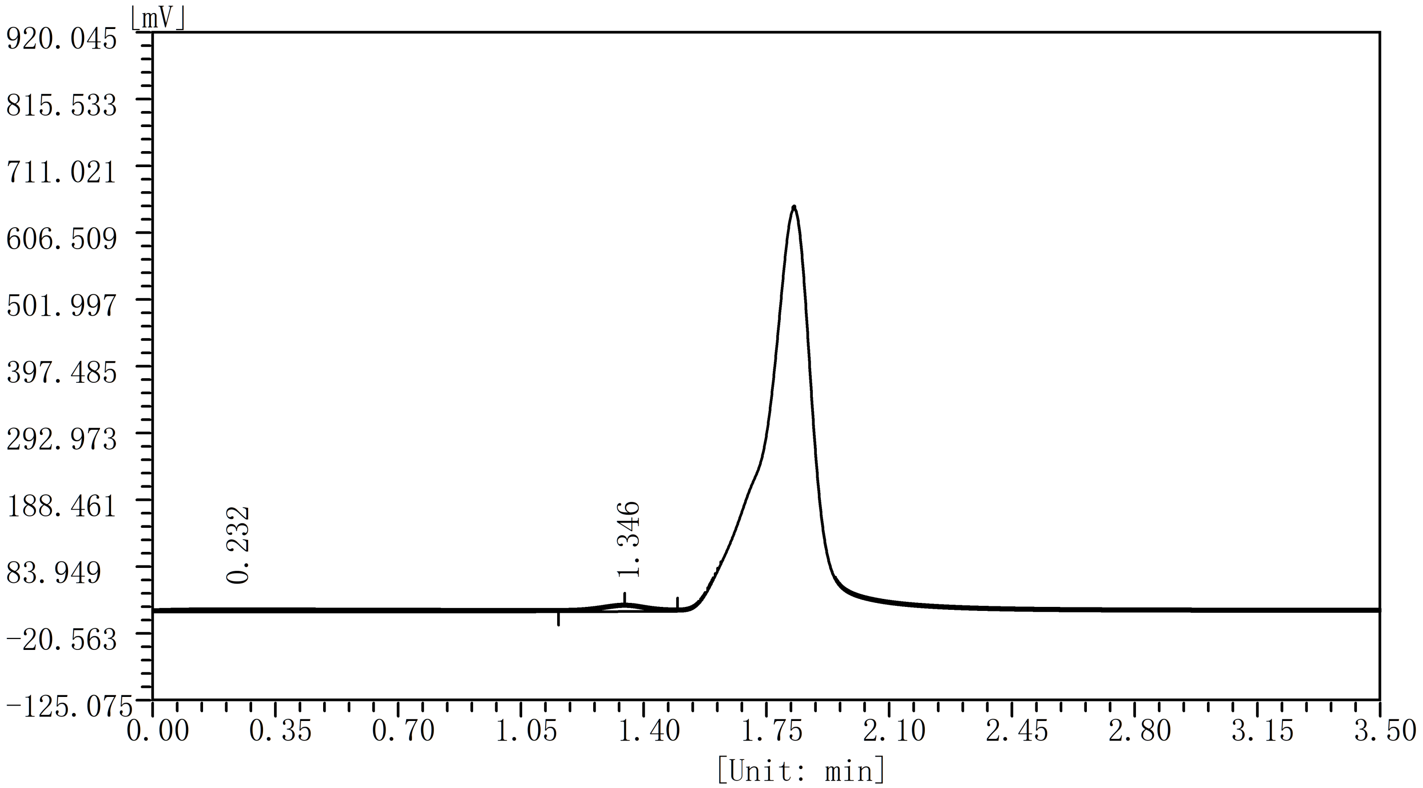

分析结果

峰序	组分名	保留时间 [min]	半峰宽 [min]	峰高 [uV]	峰面积 [uV*s]	峰面积 [%]	含量 [%]	峰类型
1		0.232	0.742	1263.5	54789.0	0.0000	0.0000	BV
2	H2	1.346	0.128	7866.1	63966.9	100.0000	0.0460	BB
			总计:	9129.6	118756.0	100.0000	0.0460	
