# Supplementary material for: A hydrophobic Cu/Cu2O sheet catalyst for selective electroreduction of CO to ethanol
Source: Nat Commun. 2023 Jan 31;14:501. doi: 10.1038/s41467-023-36261-1 (PMC9889799; doi:10.1038/s41467-023-36261-1)
Supplement: Supplementary file 2 — Source Data [file 41467_2023_36261_MOESM2_ESM.zip › Source data for Figure 4b and Supplementary Figure 11/Gas Products( Figure 4b)/BF1-3-21.pdf]

批次:　　　　21
实验单位:
计算方法:　　外标法
采样开始:　　2022-11-21 17:52:48
分析周期:　　18.00 min　　　　　斜率/峰宽: 100.0/1.0
谱图文件名:　BF1-3-21.src

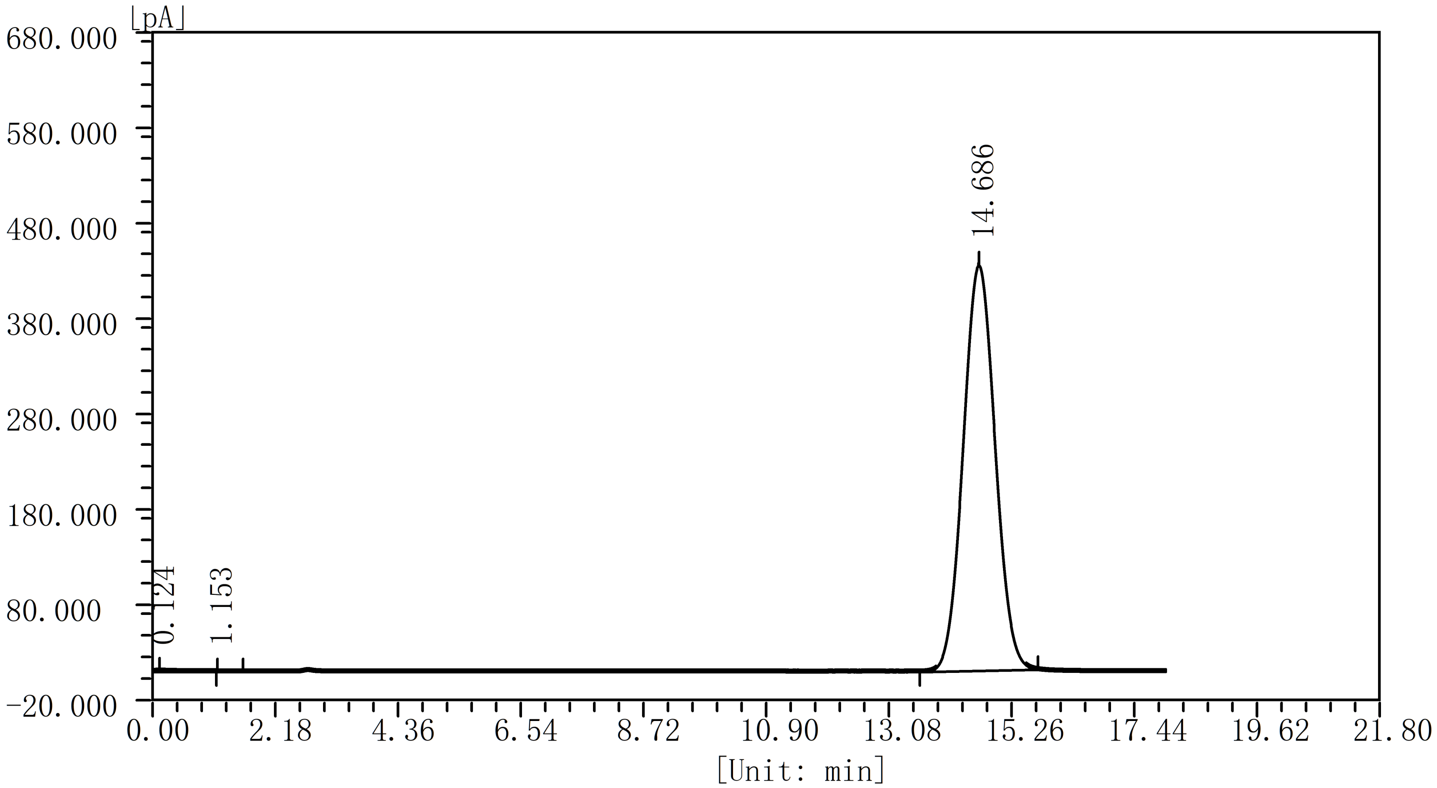

分析结果

峰序	组分名	保留时间 [min]	半峰宽 [min]	峰高 [uV]	峰面积 [uV*s]	峰面积 [%]	含量 [%]	峰类型
1		0.124	0.238	492.5	10919.8	0.0000	0.0000	BB
2		1.153	0.002	0.1	0.5	0.0000	0.0000	BB
3		14.686	0.617	425309.5	6796151.	0.0000	0.0000	BB
		总计:		425802.2	6807072.	0.0000	0.0000	
