# Supplementary material for: A hydrophobic Cu/Cu2O sheet catalyst for selective electroreduction of CO to ethanol
Source: Nat Commun. 2023 Jan 31;14:501. doi: 10.1038/s41467-023-36261-1 (PMC9889799; doi:10.1038/s41467-023-36261-1)
Supplement: Supplementary file 2 — Source Data [file 41467_2023_36261_MOESM2_ESM.zip › Source data for Figure 4b and Supplementary Figure 11/Gas Products( Figure 4b)/BF1-3-25.pdf]

批次:　　　　25
实验单位:
计算方法:　　外标法
采样开始:　　2022-11-21 19:09:24
分析周期:　　18.00　min　　　　　斜率/峰宽:　100.0/1.0
谱图文件名:　BF1-3-25.src

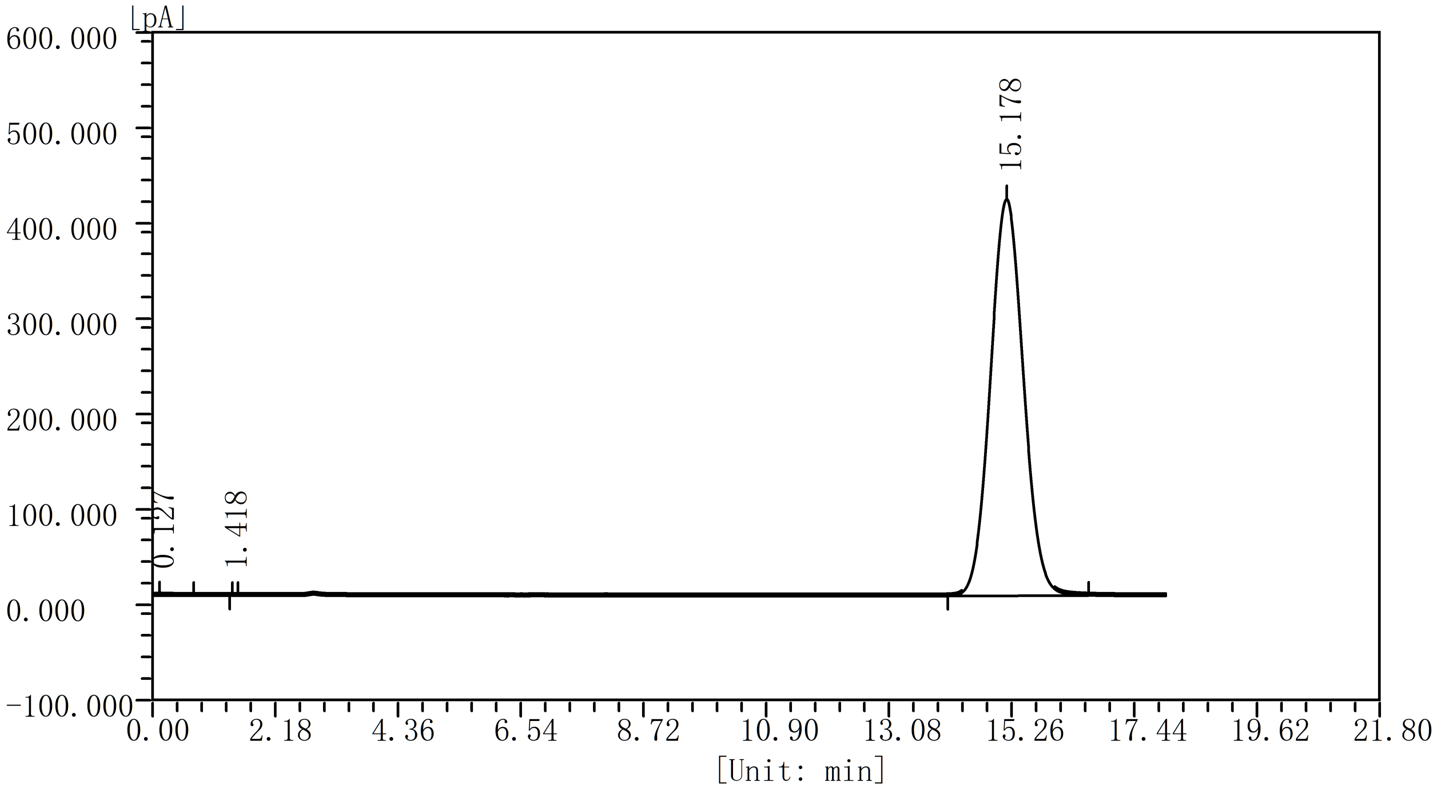

分析结果

峰序	组分名	保留时间 [min]	半峰宽 [min]	峰高 [uV]	峰面积 [uV*s]	峰面积 [%]	含量 [%]	峰类型
1		0.127	0.288	521.7	10822.3	0.0000	0.0000	BV
2		1.418	0.109	6.2	29.1	0.0000	0.0000	BB
3		15.178	0.635	415989.6	6959131.	0.0000	0.0000	BB
			总计:	416517.4	6969982.	0.0000	0.0000	
