# Supplementary material for: A hydrophobic Cu/Cu2O sheet catalyst for selective electroreduction of CO to ethanol
Source: Nat Commun. 2023 Jan 31;14:501. doi: 10.1038/s41467-023-36261-1 (PMC9889799; doi:10.1038/s41467-023-36261-1)
Supplement: Supplementary file 2 — Source Data [file 41467_2023_36261_MOESM2_ESM.zip › Source data for Figure 4b and Supplementary Figure 11/Gas Products( Figure 4b)/BF1-3-9.pdf]

批次: 9
实验单位:
计算方法: 外标法
采样开始: 2022-11-19 13:00:55
分析周期: 18.00 min 斜率/峰宽: 100.0/1.0
谱图文件名: BF1-3-9.src

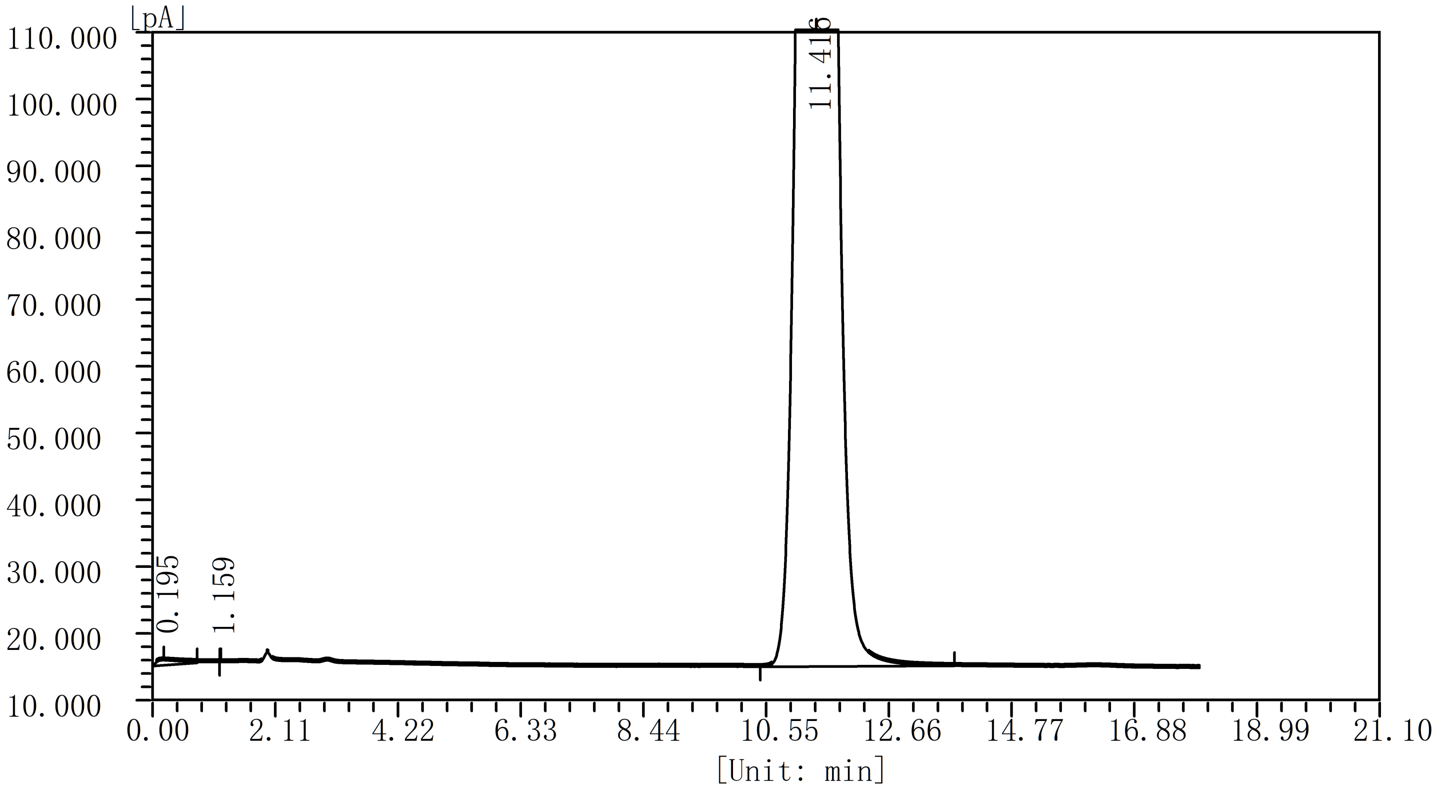

分析结果

峰序	组分名	保留时间 [min]	半峰宽 [min]	峰高 [uV]	峰面积 [uV*s]	峰面积 [%]	含量 [%]	峰类型
1		0.195	0.713	758.1	21887.9	0.0000	0.0000	BV
2		1.159	0.011	2.0	0.3	0.0000	0.0000	BB
3		11.416	0.504	419119.7	3635651.	0.0000	0.0000	BB
			总计:	419879.8	3657540.	0.0000	0.0000	
