# Supplementary material for: A hydrophobic Cu/Cu2O sheet catalyst for selective electroreduction of CO to ethanol
Source: Nat Commun. 2023 Jan 31;14:501. doi: 10.1038/s41467-023-36261-1 (PMC9889799; doi:10.1038/s41467-023-36261-1)
Supplement: Supplementary file 2 — Source Data [file 41467_2023_36261_MOESM2_ESM.zip › Source data for Figure 4b and Supplementary Figure 11/Gas Products( Figure 4b)/BT2-3-13.pdf]

批次:　　　　13
实验单位:
计算方法:　　外标法
采样开始:　　2022-11-19 16:24:46
分析周期:　　18.00　min　　　　　斜率/峰宽:　100.0/1.0
谱图文件名:　BT2-3-13.src

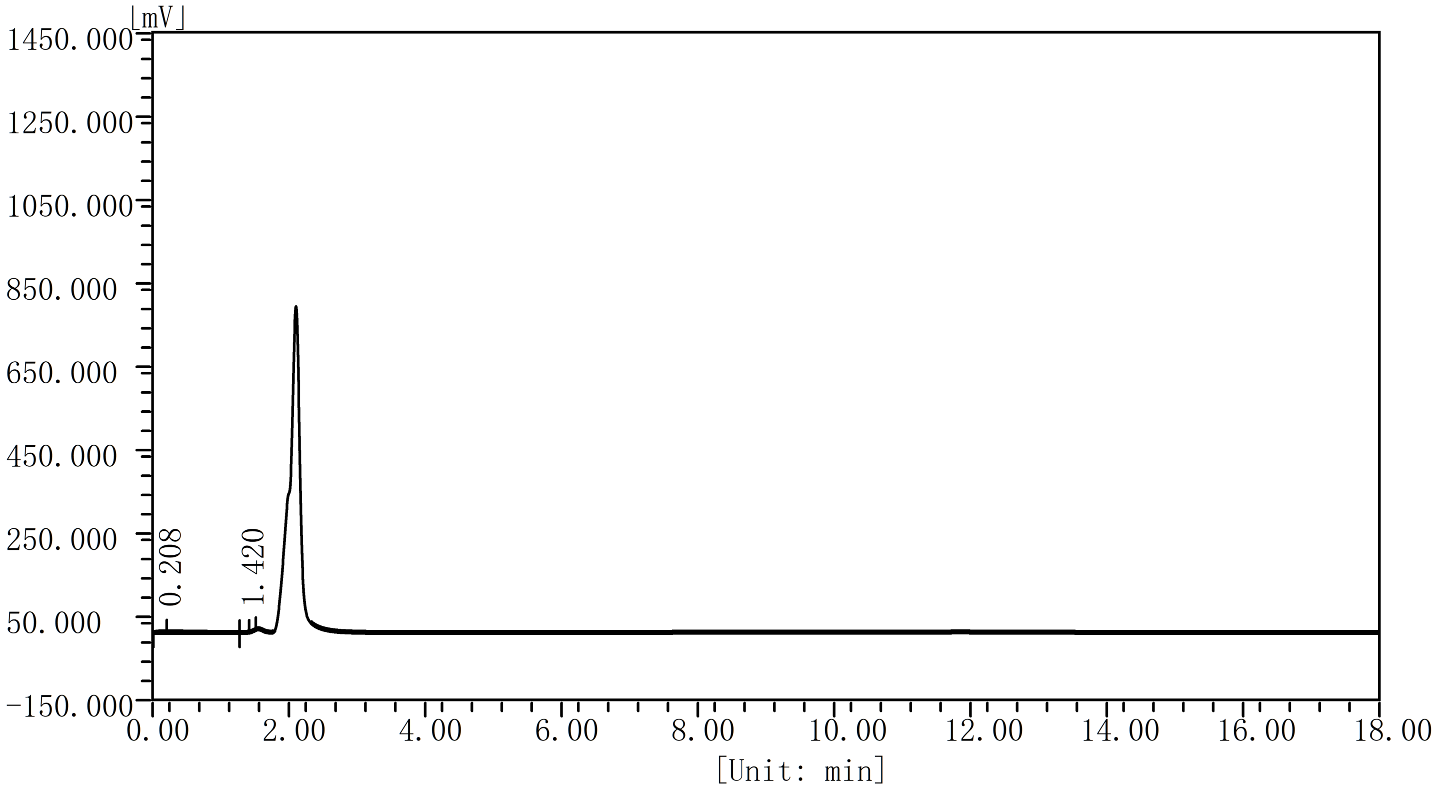

分析结果

峰序	组分名	保留时间<br>[min]	半峰宽<br>[min]	峰高<br>[uV]	峰面积<br>[uV*s]	峰面积<br>[%]	含量<br>[%]	峰类型
1		0.208	0.530	1439.6	49980.1	0.0000	0.0000	BV
2		1.420	0.179	3527.8	29977.2	0.0000	0.0000	VB
			总计:	4967.4	79957.3	0.0000	0.0000	
