# Supplementary material for: A hydrophobic Cu/Cu2O sheet catalyst for selective electroreduction of CO to ethanol
Source: Nat Commun. 2023 Jan 31;14:501. doi: 10.1038/s41467-023-36261-1 (PMC9889799; doi:10.1038/s41467-023-36261-1)
Supplement: Supplementary file 2 — Source Data [file 41467_2023_36261_MOESM2_ESM.zip › Source data for Figure 4b and Supplementary Figure 11/Gas Products( Figure 4b)/BT2-3-22.pdf]

批次: 22
实验单位:
计算方法: 外标法
采样开始: 2022-11-21 18:11:59
分析周期: 18.00 min 斜率/峰宽: 100.0/1.0
谱图文件名: BT2-3-22.src

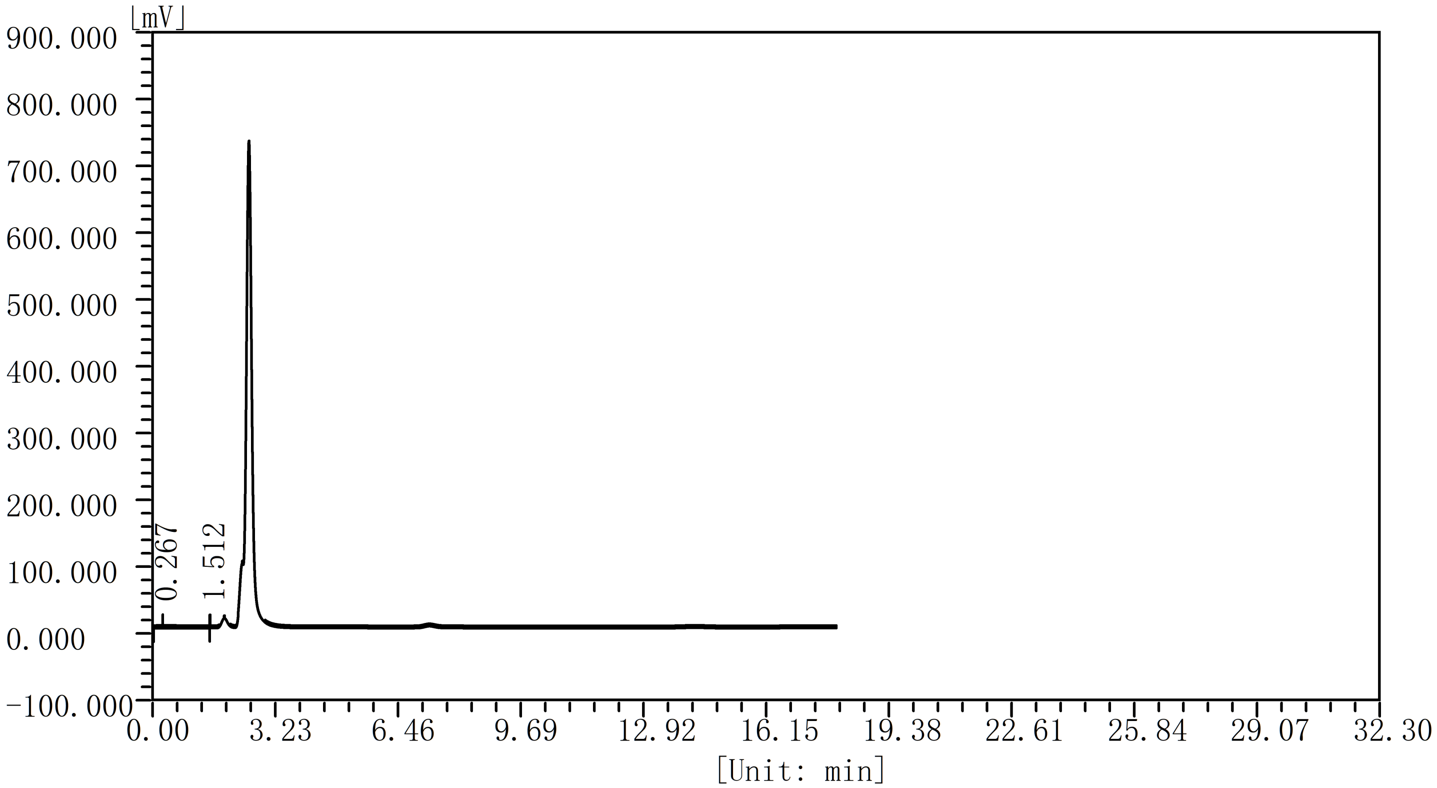


# 分析结果

| 峰序 | 组分名 | 保留时间 [min] | 半峰宽 [min] | 峰高 [uV] | 峰面积 [uV*s] | 峰面积 [%] | 含量 [%] | 峰类型 |
|---|---|---|---|---|---|---|---|---|
| 1 | | 0.267 | 0.633 | 793.1 | 30231.6 | 0.0000 | 0.0000 | BB |
| 2 | | 1.512 | 0.006 | 0.0 | 0.0 | 0.0000 | 0.0000 | BB |
| | | | 总计: | 793.1 | 30231.6 | 0.0000 | 0.0000 | |
